# Carbon Vacancies Steer the Activity in Dual Ni Carbon Nitride Photocatalysis

**DOI:** 10.1002/advs.202303781

**Published:** 2023-07-06

**Authors:** Miriam Marchi, Edoardo Raciti, Sai Manoj Gali, Federica Piccirilli, Hendrik Vondracek, Arianna Actis, Enrico Salvadori, Cristian Rosso, Alejandro Criado, Carmine D'Agostino, Luke Forster, Daniel Lee, Alexandre C. Foucher, Rajeev Kumar Rai, David Beljonne, Eric A. Stach, Mario Chiesa, Roberto Lazzaroni, Giacomo Filippini, Maurizio Prato, Michele Melchionna, Paolo Fornasiero

**Affiliations:** ^1^ Department of Chemical and Pharmaceutical Sciences, Center for Energy, Environment and Transport “Giacomo Ciamician” INSTM UdR Trieste University of Trieste Via Licio Giorgieri 1 Trieste 34127 Italy; ^2^ Laboratory for Chemistry of Novel Materials Materials Research Institute University of Mons‐UMONS Mons 7000 Belgium; ^3^ Elettra Sincrotrone Trieste Strada Statale 14 km 163.5 in Area Science Park Basovizza Trieste 34149 Italy; ^4^ Department of Chemistry and NIS Centre University of Torino Via Pietro Giuria 7 Torino 10125 Italy; ^5^ Centro Interdisciplinar de Química e Bioloxía–CICA Universidade da Coruña Rúa As Carballeiras A Coruña 15071 Spain; ^6^ Department of Chemical Engineering The University of Manchester Oxford Road Manchester M13 9PL UK; ^7^ Department of Civil, Chemical, Environmental and Material Engineering (DICAM) Alma Mater Studiorum University of Bologna Via Terracini, 28 Bologna 40131 Italy; ^8^ Department of Materials Science and Engineering University of Pennsylvania Philadelphia PA 19104‐6272 USA; ^9^ Center for Cooperative Research in Biomaterials (CIC biomaGUNE) Basque Research and Technology Alliance (BRTA) Paseo de Miramón 194 Donostia‐San Sebastián 20014 Spain; ^10^ Ikerbasque Basque Foundation for Science Bilbao 48013 Spain; ^11^ ICCOM‐CNR Unit of Trieste via L. Giorgieri 1 Trieste 34127 Italy

**Keywords:** carbon nitride, dual photocatalysis, nickel, organic synthesis

## Abstract

The manipulation of carbon nitride (CN) structures is one main avenue to enhance the activity of CN‐based photocatalysts. Increasing the efficiency of photocatalytic heterogeneous materials is a critical step toward the realistic implementation of sustainable schemes for organic synthesis. However, limited knowledge of the structure/activity relationship in relation to subtle structural variations prevents a fully rational design of new photocatalytic materials, limiting practical applications. Here, the CN structure is engineered by means of a microwave treatment, and the structure of the material is shaped around its suitable functionality for Ni dual photocatalysis, with a resulting boosting of the reaction efficiency toward many C—X (X = N, S, O) couplings. The combination of advanced characterization techniques and first‐principle simulations reveals that this enhanced reactivity is due to the formation of carbon vacancies that evolve into triazole and imine N species able to suitably bind Ni complexes and harness highly efficient dual catalysis. The cost‐effective microwave treatment proposed here appears as a versatile and sustainable approach to the design of CN‐based photocatalysts for a wide range of industrially relevant organic synthetic reactions.

## Graphical abstract

Engineered defects are achieved by means of microwave irradiation of graphitic carbon nitride. The protocol allows formation of carbon vacancies which critically evolve into enrichment of the carbon nitride with triazole and imine moieties. Such moieties are suitable binding sites for homogeneous Ni complexes, allowing superior dual semiheterogeneous photoredox catalysis for many C—N, C—O, and C—S coupling reactions.

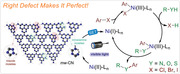

## 1 Introduction

Semiheterogeneous “dual photoredox catalysis” for organic synthesis merges the cost‐effectiveness of visible light‐absorbing semiconductors (SCs) with the efficiency of homogeneous transition metal complexes (TMCs).^[^ ^1^ ^]^ Within this framework, the in situ combination of graphitic carbon nitride (*g*‐CN) as the SC, and nickel as the TMC is attracting increasing popularity,^[^ ^2^ ^]^ due to the high abundance of the constituting elements. Dual *g*‐CN/Ni photoredox catalysis is a flexible strategy to accomplish challenging transformations, including C—C and C—X (X = nonmetal heteroatom) couplings, between complex molecules.^[^ ^2^, ^3^ ^]^ Nevertheless, this catalytic approach implies a great intricacy of action, which in most cases remains largely unknown, as the dual mechanism is governed by the elusive interaction between the heterogeneous SC and the homogeneous Ni complex. As an example, preparation of esters by means of C—O cross‐coupling involving a carbon nitride photocatalyst was reported to require the use of Ni complexes with a specific ligand environment.^[^ ^3e^ ^]^ Moreover, a common problem with dual photoredox catalysis involving homogeneous Ni species is the gradual deactivation of the catalyst due to excessive reduction to inactive Ni(0). This problem was investigated by Gisbertz et al., who indicated that formation of Ni black was the main reason for low activity of photoredox/nickel‐catalyzed C—N cross‐couplings.^[^ ^3a^ ^]^ It becomes therefore evident that the applicative potential of this strategy is still in its blooming stage, making imperative a fully rational development of *g*‐CN/Ni dual photocatalytic systems that maximize activity overcoming the current limitations. One possibility is to prepare a fully heterogeneous photocatalyst incorporating Ni single atoms within the *g*‐CN framework, as was recently reported by Vijeta et al.^[^ ^3g^ ^]^ Single atom catalysts (SACs) are indeed one of the most rapidly emerging classes of catalysts, although their synthesis and stability often pose serious hurdles.^[^ ^4^ ^]^ In fact, they can suffer from leaching, which prevents continuous use of the catalyst, from typical low loadings of the single metal atom, causing low activity, or the concomitant formation of small clusters during synthesis, which creates uncertainty on the identity of the truly active site.^[^ ^5^ ^]^ Most importantly for organic synthesis, the use of SACs may be restricted to specific types of reactions given the fixed coordination environment of the metal atom throughout the catalytic cycle,^[^ ^6^ ^]^ while semiheterogeneous systems are potentially more flexible as the metal species performs most of the catalysis in solution so that the coordination environment can be adjusted more easily. Hence, utilization of SAC–CN photocatalysts must also hinge on controlled engineering of the CN structure so to suitably anchor Ni atoms while maintaining high activity and be of flexible use in a variety of catalytic reactions. Although it is expected that CN–SAC synthesis will be in the long run perfected to make it adaptable to the particular reaction, the possibility of relying on a semiheterogeneous analog where the Ni environment is defined in solution phase rather than on the CN structure represents a highly valuable alternative to SAC. Notably, *g*‐CN represents a versatile platform to explore the introduction of task‐driven functionalities, whereby bottom‐up and top‐down methods can be used to modify the structure of CN according to the specific function. Therefore, knowledge of the required Ni coordination environment of the catalytic Ni species has itself benefits for aiding SAC design and synthesis. Most applications have focused on energy‐related processes,^[^ ^7^ ^]^ while photocatalytic organic synthesis has remained a relatively unbeaten track, in particular for dual photoredox catalysis. For this latter application, an important advancement is to construct a CN material with suitable moieties for the binding and releasing of Ni intermediates during the dual photocatalytic cycle.

Here, we develop a facile yet effective approach based on postsynthetic microwave (MW) irradiation to forge a new modified CN derivative with tailored structural features. The use of MW has been hitherto employed only in the bottom‐up synthesis of carbon nitride, to assist the precursor polymerization and achieve CN material with particular optical and structural properties.^[^ ^8^ ^]^ However, it has never been applied as a controlled postsynthetic step to tailor the desired CN structures. It emerges that MW‐assisted synthesis leads to more poorly crystalline materials with a too defective structure that can negatively affect activity, whereas in our postsynthetic protocol, the extent of carbon vacancies created by the MW treatment is purposely moderate and tuned for the task. While the generation of C‐vacancies has been the subject of several reports, our methodology is a function‐driven method that is of specific utility for the Ni dual photocatalytic reaction. In fact, the presence of a protic solvent during treatment of the already formed *g*‐CN and the controlled MW irradiation generates carbon vacancies that evolve into triazole and imine units, which are able to readily coordinate the Ni centers. The result is an immediate and clear advantage in terms of photocatalytic efficiency of the dual catalytic system. Therefore, it sharply distinguishes itself from other more invasive and/or less cost‐ and time‐effective methods based on high‐temperature treatments,^[^ ^9^ ^]^ steam etching,^[^ ^10^ ^]^ or He^+^‐ion irradiation.^[^ ^11^ ^]^ The fundamental aspects that govern the interaction and electron transfer dynamics between the new mw‐CN heterogeneous photocatalyst and the Ni species were unraveled by means of various advanced experimental techniques. Moreover, as also confirmed by theoretical calculations, the coordination energy is adjusted in order to fulfil the stringent requirements of a dual semiheterogeneous photocatalytic mechanism. As a result, the catalytic system triggers several C—N, C—O, and C—S coupling reactions with high efficiency. While such three coupling reactions have been individually reported with CN/Ni dual photocatalysis,^[^ ^3a,e^ ^]^ the present mw‐CN explores the whole landscape of C—X (X = N, O, S) showing an excellent versatility, and overcoming in many cases the activity of other benchmark CN photocatalysts. For example, the production over the CN surface area for the model C—N coupling reaction between pyrrolidine and 4‐bromobenzoate proceeds with up to one order of magnitude higher rate than that of benchmark photocatalysts.^[^ ^1c^ ^]^ Notably, C—X coupling reactions are of crucial importance for the preparation of active pharmaceutical ingredients (APIs),^[^ ^12^ ^]^ therefore justifying the urgency in the development of new economically viable catalytic approaches. In a broader context, the present results will be also of important guidance for the engineering of *g*‐CN surfaces that can harness single‐atom‐based photocatalysis for organic synthesis.

## 2 Results and Discussion

### 2.1 Material Synthesis and Characterization

**Figure 1:**
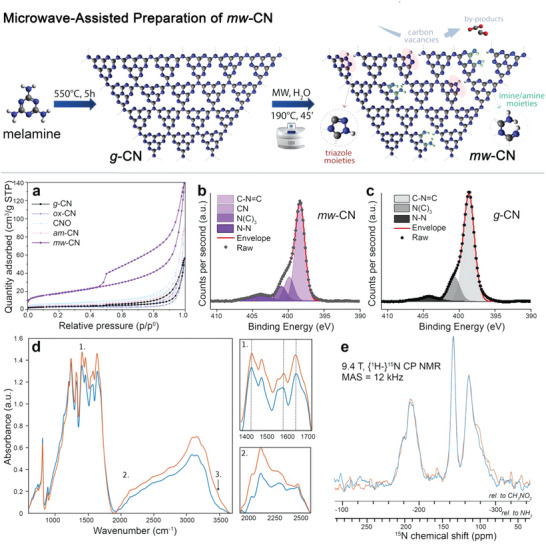
Synthetic scheme and materials characterization. Top: sketch of the synthetic procedure for mw‐CN; evolution of CO or CO_2_ following C‐vacancies creation is only a tentative hypothesis. a) N_2_ physisorption isotherms at liquid nitrogen temperature of mw‐CN, *g*‐CN, and other reference CN materials. b,c) N1s High‐resolution X‐ray photoelectron spectroscopy spectra for *g*‐CN and mw‐CN with peak deconvolution shown. d) FTIR absorption spectra of *g*‐CN (blue) and mw‐CN (orange) in KBr pellets 10% w/w. e) {^1^H}^15^N CPMAS NMR spectra of g‐CN (blue) and mw‐CN (orange), recorded at 9.4 T using a MAS frequency of 12 kHz at ambient temperature. The peak assignment follows ref. [24].

As evident from recent analyses, *g*‐CN shines as the most rapidly emerging photocatalyst,^[^ ^13^ ^]^ although it suffers from typically very low surface area, requiring the use of ever more sophisticated synthetic approaches.^[^ ^14^ ^]^ High surface area is generally accepted as a strong prerequisite for efficient (photo)catalysis, although other factors such as charge mobility and fast charge recombination may be the overriding reason for low activity,^[^ ^15^ ^]^ calling for a task‐specific tuning of defect distribution.^[^ ^16^ ^]^ Although less explored than N‐vacancies,^[^ ^17^ ^]^ the formation of C‐vacancies within the carbon nitride structure has been previously achieved by means of various methods based on high‐temperature treatments or chemical processes and utilized for energy or environmental‐related processes. For example, Dong et al. exploited the presence of cyanuric acid during melamine polycondensation to introduce abundant carbon vacancies, which were critical to improve charge carrier separation and diffusion. The resulting photocatalysts showed enhanced photoreduction activity of NO to N_2_.^[^ ^9b^ ^]^ Thermal treatment at 510 °C under NH_3_ atmosphere was reported to prepare “holey” graphitic carbon nitride, where C‐vacancies were responsible for extending the charge lifetime and led to higher H_2_ evolution performances.^[^ ^9c^ ^]^ Use of steam etching approaches led to removal of C atoms with development of —NH_2_ groups on the carbon nitride surface, and the photocatalyst could successfully activate the photoreduction of CO_2_ to CO.^[^ ^10^ ^]^ Formation of —NH_2_ moieties was also observed by Li et al. who reported the formation of C‐vacancies by thermal treatment. The as‐formed —NH_2_ groups were instrumental to provide binding sites for O_2_ which could be reduced to H_2_O_2_.^[^ ^9a^ ^]^ Herein, we induce C‐vacancies by a low‐energy and industrially appealing microwave treatment of the pristine *g*‐CN in environmentally compatible water medium (**Figure** **1**). This strategy leads to a different type of defective carbon nitride (mw‐CN) where the formation of C‐vacancies is more moderate but crucially results in evolution of the structure into formation of triazole and imine moieties, which can be fruitfully exploited to anchor Ni complexes for dual photoredox catalysis and trigger many C—X coupling reactions. C‐vacancies formation occurs with concomitant enhancement of the material's surface area and mesopore density, as compared to the conventional thermally generated *g*‐CN. N_2_ physisorption analysis shows the hysteresis loop of type IV isotherms, typical of mesoporous materials (Figure 1a), which is much more pronounced than that found with other methods of modification of CN, such as mild oxidation (ox‐CN), strong oxidation (CNO),^[^ ^18^ ^]^ and amorphization (am‐CN).^[^ ^19^ ^]^ Moreover, mw‐CN has a bimodal pore distribution, with one maximum at smaller diameters (40 nm) than the reference CNs, as well as a contribution from even smaller mesopores (Figure S1, Supporting Information). This more porous structure makes up for a larger cumulative pore volume (0.22 cm^3^ g^−1^), and in turn a Brunauer–Emmett–Teller (BET) surface area of 61.6 m^2^ g^−1^, sixfold higher than that of the pristine *g*‐CN (**Table** **1**). Interestingly, pulse‐field gradient nuclear magnetic resonance (NMR) diffusion measurements show an improved pore connectivity in mw‐CN (Figure S9 and Tables S6 and S7, Supporting Information). The change in texture is accompanied by only small changes of the optical absorption properties, with the bandgaps calculated from Tauc plot analysis of the ultraviolet–visible diffuse reflectance spectroscopy having similar values for *g*‐CN and mw‐CN, thus not compromising the redox ability of the catalyst (Figure S1 (Supporting Information) and Table 1).

**Table 1:** Textural properties, bandgap, and XPS data

Sample	BET surface area [m^2^ g^−1^]	Cumulative pore volume [cm^3^ g^−1^]	Diameter pore maximum [nm]	Bandgap [eV]	C at %	N at %	O at %	C/N
*g*‐CN	10.2	0.09	56	2.64	42.3	57.7	–	0.73
am‐CN	14.8	0.14	108	2.49	43.1	56.9	–	0.76
ox‐CN	7.4	0.09	57	2.68	41.9	52.7	5.4	0.79
CNO	21.8	0.15	58	2.93	39.8	52.8	7.4	0.75
mw‐CN	61.6	0.22	40 and 7 (bimodal distribution)	2.66	40.2	57.1	2.6	0.70

From X‐ray photoelectron spectroscopy (XPS), we note that in the mw‐CN, the deconvolution of the N1s shows, together with the two characteristic CN peaks at 398.3 eV (C=N—C) and 401.0 eV (N(C)_3_),^[^ ^19b^ ^]^ an additional contribution at 399.9 eV (Figure 1b,c), which is generally assigned to N—H moieties.^[^ ^20^ ^]^ Concomitantly, the overall C at% in mw‐CN is appreciably lower, with the C/N ratio dropping to 0.70, the lowest of all the measured CN derivatives (Table 1). A consistent interpretation is that the MW process generates C‐vacancies within the heptazine framework. On one hand, C abstraction from *g*‐CN by steam etching was shown to proceed with release of CO, CO_2_, and H_2_, leading to formation of —NH_2_ groups on the surface.^[^ ^10^ ^]^ In accordance, we tentatively propose the evolution of CO and CO_2_ during MW treatment. On the other hand, introduction of C‐vacancies into graphitic CN, achieved under high‐temperature treatment of the solid under various types of atmosphere,^[^ ^9^, ^21^ ^]^ was reported to proceed with the formation of unpaired electrons on nitrogen sites.^[^ ^22^ ^]^ Possibly, the two radical species could abstract a hydrogen atom from the solvent leading to formation of imine and amine groups. Alternatively, the two nearby radicals on the N atoms may also couple to form a N—N bond leading to a triazole moiety, and this would explain the higher intensity observed for the 404.6 eV component in the XPS spectrum, which has been attributed to N—N bonding.^[^ ^23^ ^]^ Table S1 (Supporting Information) reports on the increase of the percent of the N—N component, which reaches 9.6 at%, higher than the N—N content present in all other investigated CN catalysts where the value spans from 5.4% to 6.3%. In agreement with this hypothesis, Q‐band electron paramagnetic resonance (EPR) spectra of mw‐CN and *g*‐CN indicate a comparable amount of native *S* = 1/2 paramagnetic defects (Figure S14a, Supporting Information), implying that no additional radicals are introduced within the mw‐CN. Moreover, the difference between *g*‐CN and mw‐CN in terms of number of paramagnetic defects is also not significant under irradiation, suggesting the idea that the increase in performance is not associated to a better photoelectric efficiency of the mw‐CN but rather in its structural functionalities in relation to Ni binding.

**Figure 2:**
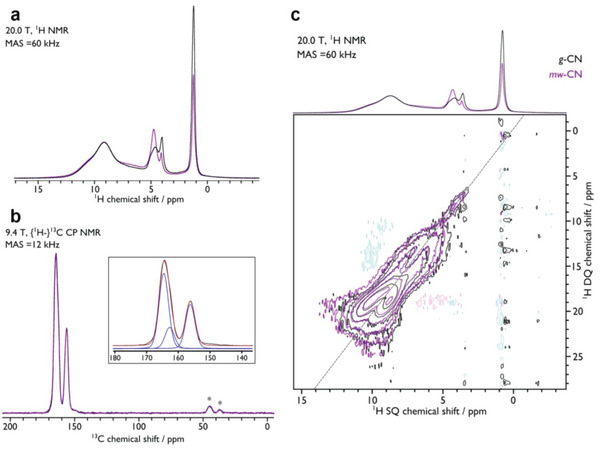
a) ^1^H Hahn‐echo MAS NMR spectra of *g*‐CN (black) and mw‐CN (purple), recorded at 20.0 T using a MAS frequency of 60 kHz at ambient temperature. b) {^1^H–}^13^C CPMAS NMR spectra of *g*‐CN (black) and mw‐CN (purple), recorded at 9.4 T using a MAS frequency of 12 kHz at ambient temperature. The inset shows the peak deconvolution. c) 2D ^1^H MAS NMR homonuclear Double quantum–single quantum (DQ–SQ) dipolar correlation spectra of *g*‐CN (black) and mw‐CN (purple), recorded at 20.0 T using the *S*_2_ recoupling sequence and a MAS frequency of 60 kHz at ambient temperature. The dashed line highlights the DQ diagonal and the corresponding 1D spectra are reproduced from (a) above for comparison. The 2D DQ–SQ spectrum in (c) aids the assignment of the ^1^H resonances, which are consistent with those for similar polymeric carbon nitride.^[^ ^24a^ ^]^ The absence of a correlation peak with the resonance at *δ*{^1^H SQ} = 1.3 ppm indicates that this must correspond to discrete protons that are >2 Å from another and as such corresponds to lone H on bridging N (—NH—). The autocorrelation peaks at *δ*{^1^H SQ} = 4–5 ppm, which lack cross‐correlation peaks, correspond to nonhydrogen‐bonded NH_2_ groups. The correlation peaks at *δ*{^1^H SQ} = 6–8 ppm correspond to H on bridging N that are in proximity to other protons, possibly indicating weak H‐bonding, while those at *δ*{^1^H SQ} = 8–11 ppm correspond to protons involved in strong H‐bonding, either from NH_2_ or H on bridging N.

Moreover, electron nuclear double resonance (ENDOR) experiments reveal that the local proton environments of such defects show no substantial difference for the two samples (Figure S13b, Supporting Information), and that the average electron–proton distance is of ≈4.3 Å, compatible with a structure based on the tri‐*s*‐triazine unit assembly.^[^ ^25^ ^]^ The ^15^N solid‐state nuclear magnetic resonance (ssNMR) spectra (Figure 1e), however, do show a difference in the terminal —NH_2_ region (region between −270 and −300 ppm relative to CH_3_NO_2_). This region is related to heterogeneous —NH_2_ environments, partly due to twisting of the tri‐*s*‐triazine chains to facilitate a porous structure.^[^ ^24a^ ^]^ mw‐CN exhibits stronger signal intensity in this region, and thus a higher proportion of these —NH_2_ environments compared to *g*‐CN. This is corroborated with high‐field (20 T) fast magic angle sample spinning ^1^H NMR spectra (**Figure** **2**a), which show increased accessible NH protons in mw‐CN. All data converge toward the introduction of N—H moieties and ordered pore formation. However, an exact visualization of the pore order within the material is beyond NMR possibilities, as well as of high challenge with other techniques due to the large morphologic inhomogeneity of CN.

This is further verified by Fourier transform infrared (FTIR) spectroscopy with nanoscale resolution, where differences between *g*‐CN and mw‐CN are particularly remarkable in the 1400–1700 cm^−1^ range (Figure 1d), associated with various vibration modes of the heptazine skeleton.^[^ ^26^ ^]^ An enhancement of the signal over the full range is found for mw‐CN in comparison to *g*‐CN. In particular, the peak at about 1406 cm^−1^ blueshifts by 4 cm^−1^. The blueshift of heterocycle vibrational modes can be associated with the formation of carbon vacancies.^[^ ^27^ ^]^ The peak at about 1575 cm^−1^ presents an intensity enhancement in mw‐CN with respect to *g*‐CN, particularly evident when compared with the adjacent peak at 1545 cm^−1^. In the range 2800–3600 cm^−1^, the region associated with O—H and N—H stretching,^[^ ^28^ ^]^ a similar intensity enhancement for mw‐CN is found. The other remarkable feature is the enhancement of the absorbance in the region 2000–2300 cm^−1^, suggesting the formation of a denser population of —NH_2_ in the mw‐CN. We also investigated these structural alterations by means of imaging nano‐IR of three selected signals at 1638, 1400, and 1575 cm^−1^. As shown in the absorption maps (Figure S11, Supporting Information), those three functional signals show a larger absorption across the mw‐CN material. The statistical analysis, performed through the observation of height distribution plots, revealed that the absorption is more intense for mw‐CN.

Raman and X‐ray diffraction analysis confirm that formation of C‐vacancies does not affect the crystallinity of the material, with peaks retaining the same width and intensity as those of *g*‐CN, and in contrast with am‐CN, where a broadening of the characteristic signals is observed (Figure S1, Supporting Information).

### 2.2 Photocatalytic Experiments

Afterward, the mw‐CN photocatalyst was investigated toward several C—N, C—O, and C—S coupling reactions. The photocatalytic tests were first performed on the model C—N coupling reaction between pyrrolidine and methyl 4‐bromobenzoate (**Table** **2**), under low‐intensity visible light irradiation (*λ* = 450 nm). The loading of the different CN photocatalysts was set at low value (2.5 mg mL^−1^) in order to allow a meaningful comparison of the activity by the different screened catalysts (Table S2, Supporting Information). At this loading, *g*‐CN presents an activity of 48%, while with ox‐CN, am‐CN, and CNO, the performance is considerably decreased (Entries 1–4, Table 2). These results rule out a mere surface area or bandgap effect (both CNO and am‐CN have larger surface areas than *g*‐CN, while am‐CN has a narrower bandgap), or oxygen functional groups effect. Remarkably, mw‐CN surpasses all the other SC reaching an activity of 87% (Entry 5, Table 2), and raising to 97% if a higher intensity Kessil lamp (200 W m^−2^) is employed (Entry 6, Table 2). A comparison of the rate of production (expressed in mmol g_cat_^−1^ h^−1^) by mw‐CN with that of the benchmark mpg‐CN reveals that the new material is fivefold more efficient (3.0 mmol g_cat_^−1^ h^−1^ of for mw‐CN vs 0.67 mmol g_cat_^−1^ h^−1^ for mpg‐CN), indicating a larger intrinsic activity. As recommended by recent new guidelines,^[^ ^13^ ^]^ the comparison was also made in terms of activity per photocatalyst surface area, where (considering the higher surface area of mpg‐CN in comparison with mw‐CN) the rate of production of our photocatalyst exceeds that of mpg‐CN by about one order of magnitude (4.87 × 10^−2^ mmol m_cat_^2^ h^−1^ for mw‐CN and 4.79 × 10^−3^ mmol m_cat_^2^ h^−1^ for mpg‐CN).^[^ ^1c^ ^]^ This further confirms that the higher activity mostly depends on the catalyst structure. The dual photocatalytic nature of the reaction is checked by control experiments carried out without light (Entry 7, Table 2), without Ni source (Entry 8, Table 2), or without SC (Entry 9, Table 2), all giving negligible activity. A control experiment was also performed at open air conditions (Entry 10, Table 2) and no activity was observed.

**Table 2:** Comparison of activity between the various CN photocatalysts for the model C—N coupling reaction depicted in the scheme

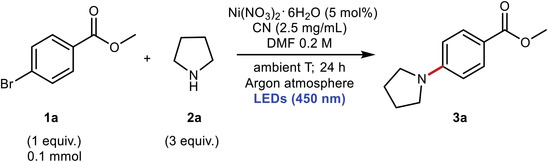			
Entry	CN photocatalyst	Deviation	Yield 3a [%]
1	*g*‐CN	None	48 ± 5
2	am‐CN	None	7 ± 5
3	ox‐CN	None	24 ± 2
4	CNO	None	5 ± 2
5	mw‐CN	None	87 ± 8
6	mw‐CN	Kessil lamp (456 nm)	97 ± 3
7	mw‐CN	In the dark	0
8	mw‐CN	No [Ni] catalyst	0
9	None	None	0
10	mw‐CN	In air	0

The stability of the mw‐CN/Ni catalytic system was compared to that of the *g*‐CN/Ni, by checking the catalytic activity after recycling of the catalyst. The former undergoes a partial deactivation, with the observed yield for the model reaction dropping from 97% to 50%, meaning a loss of activity < 50%. On the other hand, the activity of the recycled *g*‐CN/Ni goes from 48% to 12%, which is a loss of about 75%, therefore highlighting the better stability of the mw‐CN photocatalyst.

### 2.3 Mechanistic Investigations

**Figure 3:**
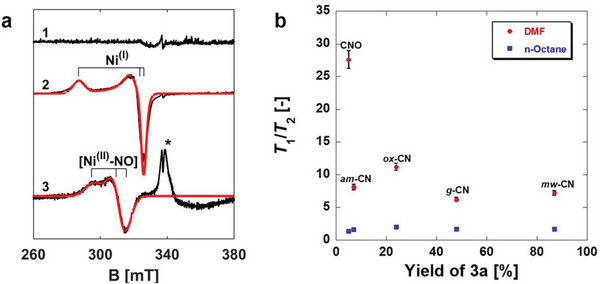
EPR and NMR spectroscopic analysis. a) Normalized CW‐EPR spectra of 1) dehydrated sample, 2) reduced sample, and 3) dehydrated sample in the presence of 10 mbar NO. All spectra were recorded at −196 °C. Computer simulations of the spectra are reported in red and the corresponding spin‐Hamiltonian parameters are listed in Table S6 (Supporting Information). The asterisk in spectrum 3 indicates the signature of physisorbed NO molecules. b) *T*_1_/*T*_2_ of DMF (gray triangles) and *n*‐octane (red triangles) imbibed within the pores of the various CN photocatalysts, as determined using low‐field NMR relaxation measurements as a function of the yield of 3a. NMR relaxation measurements were recorded at 20 °C and atmospheric pressure. The error of all *T*_1_/*T*_2_ values is ≈4%.

The enhanced surface area and the large mesopore distribution imply both a higher density of photoexcited charge carriers and a facile diffusion of the Ni TMC to the surface of the semiconductor. The structure of the Ni complex on the mw‐CN was studied by a combination of scanning transmission electron microscopy (STEM), EPR, and density functional theory (DFT) and pointed toward additional important aspects. Ni was bound to mw‐CN (and *g*‐CN for comparison) under catalytic mixture conditions in the dark. Noteworthy, inductively coupled plasma mass spectrometry (ICP‐MS) analyses of these samples reveal that when the Ni content is calculated over the bulk materials, the value is very small (525 ppm for mw‐CN and 152 ppm for *g*‐CN). This confirms that most of the Ni coordinates on the surface of the CN materials (see the EPR discussion below). High‐angle annular dark‐field scanning transmission electron microscopy (HAADF‐STEM) and electron energy loss spectroscopy (EELS) of isolated mw‐CN and *g*‐CN materials confirm the morphology and crystallinity retention after Ni complexation (Figure S4, Supporting Information). In EPR experiments (**Figure** **3**) designed to probe the presence and chemical reducibility of Ni^2+^ species, the sample was reduced under H_2_ atmosphere (30 mbar) at 300 °C for 30 min. After this treatment, the characteristic EPR signal of isolated single Ni^+^ species featuring *S* = 1/2 state with dominant occupancy of the metal 3d(*x* ^2^−*y* ^2^) orbital was observed (spectrum 2 in Figure 3a), consistent with a pseudo‐square‐planar coordination.^[^ ^29^ ^]^ Quantitative analysis indicates that under these conditions, 3.8% of the total Ni in the sample is reducible. The quantification was based on XPS measurements of Ni content in mw‐CN, where the total Ni content was of 2.8 at%. Furthermore, to probe the chemical accessibility of Ni^2+^ species, the sample was contacted with NO (10 mbar). The characteristic EPR spectrum of the [Ni^2+^–NO] adduct (*S* = 1/2, spectrum 3 in Figure 3a)^[^ ^29^, ^30^ ^]^ demonstrates the presence of chemically accessible Ni^2+^ species at the surface.

Based on the XPS, ssNMR, and EPR data, DFT quantum‐chemical calculations were performed on model complexes to shed light on the structure and electronic properties of the active species (the modeling protocol is described in the Supporting Information). First, the Ni^2+^ ion was complexed by four *N*,*N*‐dimethylformamide (DMF) solvent molecules in a square planar configuration, and the capacity of one pyrrolidine molecule (the model amine used in our photocatalytic tests) to displace one DMF ligand was assessed. The complex with one pyrrolidine and three DMF molecules around the Ni center is more stable by about 9 kcal mol^−1^ than its [Ni(DMF)_4_]^2+^ counterpart (Table S8, Supporting Information).

**Figure 4:**
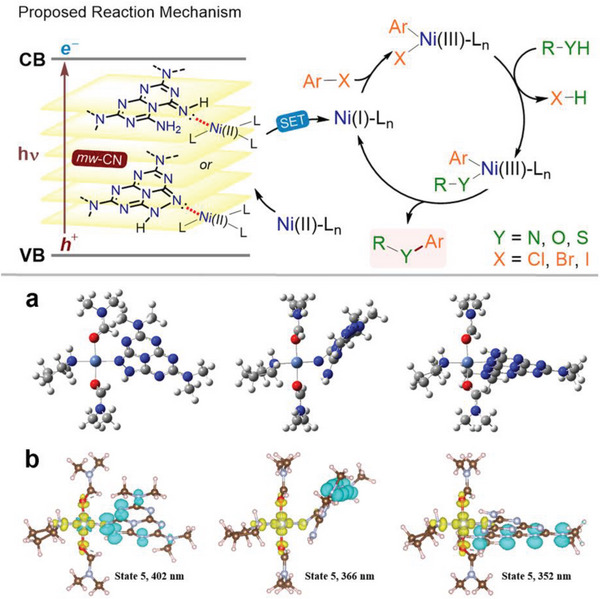
Dual photoredox catalysis mechanism and (time‐dependent) DFT calculations. Top panel: sketch of the proposed mechanism. a) DFT‐optimized geometries of Ni complexes where mw‐CN is bound via the tertiary nitrogen of the triazole ring (left), the imine nitrogen (center), and a tertiary nitrogen of pristine heptazine (right). b) Attachment/detachment densities for selected excited states in the Ni complexes where mw‐CN is bound via the tertiary nitrogen of the triazole ring (left), the imine nitrogen (center), and a tertiary nitrogen of pristine heptazine (right); the distribution of the hole (electron) density is represented in cyan (yellow).

DFT calculations were then used to assess the binding of mw‐CN to nickel. Three heptazine‐based model systems were selected to represent various defects experimentally identified in mw‐CN and pristine *g*‐CN (Figure S16, Supporting Information) and those species were used as ligands for the Ni ion, replacing a second DMF molecule (**Figure** **4**a). The computed complexation energies (Table S9, Supporting Information) indicate that the imine nitrogen more strongly coordinates the metal ion than a DMF molecule does and that the tertiary N atom of the triazole ring also tends to strongly coordinate it, which implies that the Ni complex in solution is prone to bind to those sites at the surface of mw‐CN. By contrast, the secondary nitrogen of triazole and the —NH_2_ group are weaker ligands. It is also noteworthy that the tertiary nitrogen atoms of pristine heptazine show a clear affinity toward nickel (the complexation energy is similar to that of the imine nitrogen), suggesting that the Ni complex can also bind to the CN edges. The importance of those sites has also been reported recently for proton‐coupled electron transfer processes at the CN surface.^[^ ^31^ ^]^

Based on these results, we hypothesize that the microwave treatment has a twofold beneficial effect on the photocatalytic activity: on one hand, the treatment increases the specific area of the material, as shown in Table 1. Therefore, the density of active tertiary nitrogens on heptazine units along the edges of the CN sheets is also increasing. On the other hand, the treatment generates novel active sites (imine and triazole nitrogens) competent for efficient Ni bonding and photoinduced electron transfer. Such defects are present only in very small concentration (if any) in the pristine material and their density increases upon microwave treatment, as can be visualized on the XPS spectra of Figure 1b,c where the contribution of the N—N groups (i.e., the triazole defects) at 404 eV is much larger in the microwave‐treated material.

These calculations indicate that the nitrogen sites at the surface of the mw‐CN are able to displace the solvent used (DMF) from the Ni ion, which is expected to favor the adsorption of the Ni species on the CN surface. The role of inhibition by the reaction solvent adsorption was also addressed by means of low‐field NMR relaxation measurements using DMF, and a control guest molecule, *n*‐octane, imbibed within the pores of the CN photocatalysts studied. Low‐field NMR relaxation measurements are a quick, effective, nondestructive probe of liquid–solid surface interactions where a high *T*_1_/*T*_2_ ratio is indicative of a high relative surface interaction strength.^[^ ^32^ ^]^ Such measurements can be a powerful tool for unraveling the role of surface interactions in a whole host of chemical processes^[^ ^33^ ^]^ including heterogeneous catalysis using carbon nitride photocatalysts.^[^ ^19b^ ^]^ The obtained plots, fittings, relaxation times, and calculated ratios can be seen in Figures S6 and S7 and Tables S4 and S5 (Supporting Information) and are expressed as a function of the overall reaction product yield in Figure 3b. DMF has a higher surface interaction with the photocatalyst with the lowest yield (CNO, presumably via hydrogen bonds between N and O atoms in DMF and —OH and —NH groups on the photocatalyst surface) whereas the other photocatalysts studied have a similar strength of interaction with the reaction solvent. By contrast, *n‐*octane interacts with each photocatalyst with similar strengths as expected for an alkane with no oxygenated functional groups with which to interact with surface groups on the photocatalyst.

To rationalize the photocatalytic activity, the excited states of Ni/CN complexes were modeled with time‐dependent DFT (TD‐DFT). Besides strong bands in the UV range attributed to the absorption of carbon nitride^[^ ^34^ ^]^ and vanishingly small bands expected in the 600–800 nm range due to spin‐forbidden “d–d transitions,”^[^ ^35^ ^]^ (see details in Figures S18 and S20 in the Supporting Information), the complexes show a number of singlet electronic excitations at the UV–visible boundary. This energy range is reasonably close to that of the light used for the photocatalytic experiments, considering the fact that the calculations are performed on molecular models that are smaller, hence less delocalized, than actual (defective) carbon nitride sheets. The nature of the electronic transitions, as analyzed with TD‐DFT attachment/detachment densities (Figure 4), clearly shows that these have a predominant charge transfer (CT) character from the carbon nitride species to the nickel center, in particular to the d(*x* ^2^−*y* ^2^) orbital, consistent with the EPR data. Such CT excitations are observed in both the defective structures and pristine heptazine, consistent with the fair photocatalytic activity of pristine *g*‐CN. We propose that these electronic excited states act as gateways to the photoinduced formation of Ni^+^ complexes, which subsequently enter the catalytic cycle.

Importantly, the scheme in Figure 4 implies that the complexes unbind the semiconductor after the initial Photoinduced electrin transfer reaction, which is supported by ground‐state DFT calculations performed on the complexes when imposing the nickel atom to be in the +1 oxidation state. The calculations indeed indicate that all Ni^+^ complexes, regardless of the nature of the carbon nitride ligand, are unstable (the complexation energy is positive; see Table S10 in the Supporting Information). This suggests that the nickel center dissociates from carbon nitride after the photoinduced charge transfer and that the following steps in the reaction mechanism take place in solution.

### 2.4 Reaction Scope

**Figure 5:**
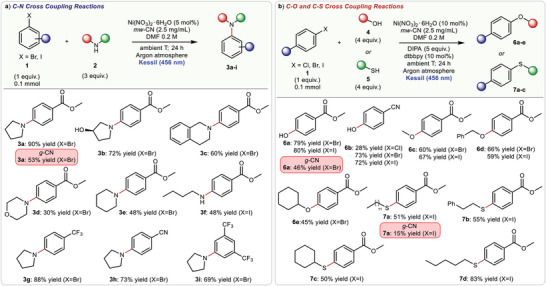
Evaluation of the scope of a) C—N, b) C—O and C—S cross‐coupling reactions.

We then evaluated the synthetic potential of the photocatalytic C—N coupling strategy by reacting differently substituted aryl halides **1** with amino derivatives **2** using mw‐CN (2.5 mg mL^−1^) as photocatalyst. As shown in **Figure** **5**, a variety of secondary amines were well tolerated under the reaction conditions, providing the desired products **3a**–**e** in good to high isolated yield (up to 90%). We also envisaged to use this photocatalytic protocol for the preparation of an API precursor.^[^ ^36^ ^]^ More specifically, we focused our attention on tetracaine, a widely used local anesthetic, which bears a butylamino aryl core that can be produced by coupling methyl 4‐iodobenzoate with butylamine. Although the coupling reaction with primary aliphatic amines is challenging and generally requires harsher conditions, the desired product **3f** was isolated in 48% yield at ambient temperature when employing our protocol.

We also found that the system is amenable to the use of other aryl halides adorned with electron‐withdrawing groups as precursors. The corresponding products **3g**–**i** were obtained in good isolated yields (69–88%). Importantly, we found out that mw‐CN is also capable of driving C—O and C—S coupling reactions under mild operative conditions and visible‐light irradiation. In particular, water has been effectively used as starting material to afford phenol derivatives **6a**,**b** (up to 80% yield). Methanol, benzyl alcohol, and cyclohexanol were also suitable substrates, providing the corresponding ethers **6c**–**e** in good yields (up to 66%). Finally, we demonstrated that mw‐CN can trigger C—S coupling reactions between thiol derivatives and aryl iodides to yield the corresponding adducts **7a**–**d**. In all cases, we obtained good yields (up to 83%). Importantly, the comparison with *g*‐CN for the model C—O or C—S coupling confirmed the much higher ability of mw‐CN to drive the photocatalytic reaction (Figure 5).

## 3 Conclusion

In conclusion, we propose a novel approach that relies on a facile microwave irradiation to prepare a multifunctional CN derivative (mw‐CN) with opportune N moieties that evolve from initial formation of C‐vacancies. Such N moieties, identified as triazole and imine units, can readily bind Ni centers, leading to very efficient and highly versatile CN–Ni dual photoredox catalytic systems for C—N, C—O, and C—S cross‐couplings.^[^ ^37^ ^]^ This approach brings together the realms of homogeneous and heterogeneous catalysis: the strong binding of the Ni^2+^ complex on the CN surface is followed by an efficient photoinduced electron transfer from the semiconductor to the complex. After the electron transfer, the release of the Ni^+^ species occurs, establishing a tuned surface–solution shuttle mechanism that is critical for the photocatalytic efficiency. The approach is versatile, as proved here by the high yields obtained for a wide range of reactions between substituted aryl halides and heteroatom (N, O, S) organic derivatives. Despite the very encouraging catalytic performances, the work's main focus goes beyond the mere enhancement of activity, but aims at better understanding the detailed substrate upon which the mechanism of Ni/CN dual photoredox catalysis is prompted. This paves the way to the design of novel dual photocatalytic systems where the suitable interaction with transition metals will considerably advance the field of metal/CN dual photocatalysis for sustainable organic synthesis toward drug developments. In particular, the application of the proposed microwave irradiation will become the pioneer platform for developing a new class of defected and functionalized CN structures, where the suitable interaction with transition metals will considerably advance the field of metal/CN dual photocatalysis. Moreover, the work offers an opportunity for future exploitation in SAC–CN fully heterogeneous photocatalysis, whereby the characteristics of the carbon nitride can be tailored by changing conditions of preparation and so accommodate Ni (or other transition metals) with a task‐driven coordination environment. This will expand the flexibility of the photocatalytic systems and trigger other photocatalytic coupling reactions, where the photocatalytic materials will be rationally designed and tailored for the specific purpose.

## 4 Experimental Section

### Materials

Commercial reagents and solvents were purchased Sigma‐Aldrich, Fluka, Alfa Aesar, Fluorochem, VWR and used as received, without further purification, unless otherwise stated.

### Methods

The microwave synthesis was performed on a CEM Discover‐SP. The solution NMR spectra were recorded on Varian 400 spectrometer (^1^H: 400 MHz; ^19^F NMR: 376 MHz; ^13^C: 100.5 MHz). The photochemical reactions were setup under an argon atmosphere in Schlenk tubes. Chromatographic purification of products was accomplished using flash chromatography on silica gel (SiO_2_, 0.04–0.063 mm, 60 Å) purchased from Machery‐Nagel, with the indicated solvent system according to the standard techniques or using a Biotage Isolera automated flash chromatography system with cartridges packed with SiO_2_ (0.04–0.063 mm, 60 Å). Thin‐layer chromatography (TLC) analysis was performed on precoated Merck TLC plates (silica gel 60 GF254, 0.25 mm). Visualization of the developed chromatography was performed by checking UV absorbance (254 nm) as well as with potassium permanganate or ninhydrin stain solution. Organic solutions were concentrated under reduced pressure on a Büchi rotary evaporator. The irradiation was performed using: a) Light emitting diode strips as light source at 450 nm, and b) Kessil lamp PR160L‐456 (50 W) purchased from Kessil webpage: https://www.kessil.com/science/PR160L.php as light source at 456 nm. Synthesis grade and anhydrous solvents were used as purchased.

Raman spectra were acquired using an Invia Renishaw spectrometer equipped with a diode laser at 785 nm. Diffuse reflectance UV–vis spectroscopy was performed with a Thermo Scientific Evolution 600 spectrophotometer, equipped with a diffuse reflectance accessory Praying‐Mantis sampling kit (Harrick Scientific Products, USA). High‐resolution mass spectra were obtained using Bruker micrOTOF‐Q (Electrospray Ionization Time‐of‐Flight). XPS for all materials was performed with a SPECS Sage HR 100 spectrometer with a non‐monochromatic X‐ray source of magnesium with a K*α* line of 1253.6 eV energy and 250 W. XPS data were fitted using CasaXPS software. N_2_ physisorption was performed with a Micrometrics ASAP 2020 analyzer at liquid nitrogen temperature. All the nanomaterials were degassed at 150 °C for 12 h at 10 µmHg. The specific surface area was calculated applying the BET method equation. Pore size distributions were determined to the adsorption branch of the isotherms with Barrett‐Joyner‐Halenda method equation.

IR absorbance spectra were acquired in transmission mode with a Vertex89v spectrometer equipped with a KBr beamsplitter, a deuterated triglycine sulfate detector, and a thermal source (Glow bar). Data were collected in the spectral range 600–4000 cm^−1^ with a resolution of 4 cm^−1^.

ICP/MS analysis was performed with the iCAP‐Q ICP‐MS equipment (Thermo Scientific, Bremen, Germany) equipped with an autosampler ASX‐500 (CETAC Technologies, Omaha, USA). The ICP/MS acquisition parameters were optimized, a 15 min equilibration and prior tuning were performed. Sample and calibrate dilutions were prepared just prior to analysis. In addition, the instrument had carried out the measurement in triplicate on all the samples, obtaining a mean value ± standard deviation. The quantification of Ni was carried out using the Qtegra v2.6 software (ThemoFisher, Bremen, Germany); monitoring the isotope ^60^Ni and ^115^In was an internal standard.

The procedure used for the sample preparation for analysis by ICP‐MS was based on acid digestion using a microwave digester, the Speedwave microwave digestion System from Berghof. Samples were dissolved in a mixture of concentrated HNO_3_ (750 µL) and HCl (250 µL). Then, digested with the method ruthenium alloys.

For the quantification of the different samples in each element, different calibration curves were developed from a CMS5 reference solution (Inorganic Ventures, Lakewood, CA, USA). The different dilutions were carried out in diluted aqua regia (2% HNO_3_/0.5% HCl) in order to obtain a medium with a similar composition of the digested samples and using correctly calibrated material. On the other hand, to improve linearity and more accurately determine detection and quantification limits, as well as minimize possible instrumental error, an internal standard (indium 50 µg L^−1^) was added to calibrations, samples, and blanks.

After analysis of the calibration solutions by ICP‐MS, the comparison between the intensity obtained from each sample and their theoretical concentrations had shown a linear range between 1 and 50 µg L^−1^ (*R* ^2^ > 0.999).

scattering‐type Scanning Near‐field Optical Microscopy (s‐SNOM) analyses were performed, thanks to the use of a Neaspec instrument (Attocube systems AG) equipped with imaging and spectroscopy modules. Measurements were performed by using a Naespec tip (arrow type, PtIr coated, 20 nm wide), in tapping mode with driving frequency of 277 kHz. Free amplitude was set to 108 nm with approaching point at 80%. These settings ensured a penetration of the radiation up to 100 nm below the sample surface. A difference frequency generation laser operating in the range 600–2100 cm^−1^ was used. Point spectra on areas 20 nm wide were acquired between 1300 and 1700 cm^−1^ and averaged, ^2^ *^n^* d‐harmonic of the imaginary part of the signal was used to reconstruct nano‐FTIR absorption (data shown in the upper panel of Figure S11 in the Supporting Information), s‐SNOM imaging was performed, thanks to PsHet detection module mounted on s‐SNOM instrument, allowing to decouple intensity and phase of the signal at a specific illumination frequency. A tunable Quantum cascade laser laser, operating in the 600–1800 cm^−1^ range, was used for the measurements at a power of 1 mW. Gwyddion software was used for image processing and statistical analysis.

Solid‐state NMR spectra were recorded using two regimes. Moderate‐field experiments employed a Bruker 9.4 T (400 MHz ^1^H Larmor frequency) AVANCE III spectrometer equipped with a 4 mm HFX magic angle spinning (MAS) probe. Experiments were acquired at ambient temperature using a MAS frequency of 12 kHz. ^1^H‐pulses of 100 kHz were used and ^13^C or ^15^N spin‐locking at ≈50 or 31 kHz was applied for 2 or 3 ms, respectively, with the corresponding ramped (70–100%) ^1^H spin‐locking matched for optimum cross‐polarization (CP) experiments with 100 kHz of SPINAL‐64^[^ ^38^ ^]^ heteronuclear ^1^H decoupling used throughout signal acquisition. The ^1^H *T*_1_ was measured with a saturation recovery experiment to be 3.7 and 2.3 s for the *g*‐CN and mw‐CN samples, respectively. 8192 transients were coadded for the {^1^H–}^13^C CPMAS NMR experiments and 27 048 (*g*‐CN) and 34 176 (mw‐CN) transients were coadded for the {^1^H–}^15^N CPMAS NMR experiments. Samples were packed into 4 mm o.d. zirconia rotors and sealed with a Kel‐F rotor cap. High‐field NMR spectra, recorded at the UK High‐Field Solid‐State NMR Facility, employed a Bruker 20.0 T (850 MHz ^1^H Larmor frequency) AVANCE NEO spectrometer equipped with a 1.3 mm HXY MAS probe, and was used in double resonance mode. Experiments were acquired at ambient temperature using a MAS frequency of 60 kHz. ^1^H pulses of 100 kHz were used, except during the *S*_2_ recoupling sequence^[^ ^39^ ^]^ where 30 kHz pulses were required. One full loop of *S*_2_ recoupling was used to reintroduce the homonuclear dipolar interaction between ^1^H‐spins during both excitation and reconversion periods, giving a total mixing time of 133 µs. For the 2D homonuclear DQ–SQ *S*_2_ dipolar correlation experiments, 16 transients were acquired for each of 32 complex (STATES‐time proportional phase incrementation) rotor‐synchronized *t*_1_ increments (2 rotor periods). Samples were packed into 1.3 mm o.d. zirconia rotors under inert conditions and sealed with a Vespel rotor cap. Spectral simulations were performed in the solid lineshape analysis module v2.2.4 in Bruker TopSpin v4.0.9. The ^1^H and ^13^C NMR chemical shifts were referenced to neat tetramethylsilane externally, and the ^15^N NMR chemical shifts were referenced to both liquid NH_3_ and CH_3_NO_2_ (90% in CDCl_3_) externally.

X‐band (microwave frequency 9.45 GHz) continuous wave (CW) EPR experiments were performed on a Bruker EMX spectrometer equipped with an ER 4122 SHQ cylindrical cavity. A modulation frequency of 100 kHz and a modulation amplitude of 0.5 mT were used. All measurements were performed at 77 K. Computer simulation was performed using the EasySpin package running in Matlab.^[^ ^40^ ^]^ Q‐band (microwave frequency ≈ 33.8 GHz) CW and pulse EPR experiments were performed on a Bruker ELEXYS 580 EPR spectrometer equipped with a Bruker EN 5107D2 resonator, an Oxford Instruments CF935 liquid helium cryostat, and an ITC 503 temperature control unit. The magnetic field was measured by means of a Bruker ER035 M NMR gaussmeter. CW EPR spectra were recorded at room temperature with a modulation frequency of 50 kHz and a modulation amplitude of 0.1 mT Mims ENDOR^[^ ^41^ ^]^ spectra were recorded at −223 °C at the field position correspondent to the maximum of the echo intensity (1204.6 mT). The pulse sequence (Figure S13, Supporting Information) was *π*/2–*τ*–*π*/2–*π*_Radio frequency (RF)_–*π*/2–*τ*–echo with *t*_*π*/2_ = 16 ns, *τ* = 216 ns, *t*_*π*RF_ = 14 µs and additional waiting times of 1 µs were used before and after the *π*_RF_ pulse. The spectra were recorded in a 5 MHz window centered at the ^1^H Larmor frequency, with a 0.02 MHz spectral resolution. The total amount of nickel in the sample was estimated from XPS data as atoms g^−1^, whereas the amount of Ni^+^ was determined by double integration of the corresponding EPR signal through the Xenon software (Bruker) as spin g^−1^. The ratio of the two values yielded the percentage of reducible nickel under the experimental conditions reported.

HAADF‐STEM, energy‐dispersive X‐ray spectroscopy (EDS), and EELS were performed with a JEOL NEORM operating at 200 kV with a convergence angle of 27 mrad. The sample was prepared by dropping the powder sample on a lacey‐carbon‐coated Cu grid. For STEM imaging and EDS, the probe current was 150 pA and the camera length was 4 cm. EDS signals were collected with two JEOL EDS detectors located above the sample. For EELS, the probe current was 500 pA, and the camera length was 2 cm to maximize the signal‐to‐noise ratio. A K2 Summit camera provided by Gatan Inc. was used to collect the EELS spectra, using a 5 mm collection aperture on the Gatan Imaging Filter. The imaging and spectroscopic data were acquired and processed using Digital Micrograph software provided by Gatan, Inc.

### Preparation of the Nanomaterials

All nanomaterials were prepared using commercial reagents and solvents purchased Sigma‐Aldrich and used as received, without further purifications.

*g‐*CN—10 g of melamine was heated in muffle furnace up to 550 °C for 300 min in a covered alumina crucible with a ramping time of 5 °C min^−1^. The final product was milled in a mortar to obtain a uniform powder.

ox‐CN—0.75 g of pristine *g*‐CN was dispersed in 300 mL of a 4 m solution of nitric acid and sonicated for 5 h. The solid was filtered and washed with 500 mL of MilliQ water for 2 times and 250 mL of methanol.

am‐CN—0.75 g of *g*‐CN was heated in a boat‐shaped alumina crucible at 620 °C for 120 min with a ramping time of 2 °C min^−1^ under argon flow.

CNO—1 g of *g*‐CN powder was added into a mixture of concentrated sulfuric acid and nitric acid with a volume ratio of 1:2. After being stirred overnight, the mixture was poured into 500 mL distilled water and collected by filtration. The product was washed with distilled water for several times and dried at room temperature.

mw‐CN—200 mg of *g*‐CN was heated at 190 °C for 45 min by microwave irradiation. The sample was collected by filtration and dried at 80 °C overnight.

### Statistical Analysis

The photocatalytic experiments detailed in Table 2, Section 2.2, and in Table S2 (Supporting Information) were the results of five independent replicates. The reported errors were reported as standard deviation on the mean value. The error bars depicted in Figure 3b regarding the NMR analysis represented the standard relative error, calculated by the ratio of the standard deviation on the mean value, and it was calculated on four independent measurement replicates. The errors associated to the *T*_1_/*T*_2_ values, on the self‐diffusion coefficients and on tortuosity values reported in Table S4–S6 (Supporting Information) were also standard relative error calculated on four independent measurement replicates. Errors for the spin‐Hamiltonian parameters of Table S7 (Supporting Information) were estimated through spectral simulations by varying one parameter at the time to determine the error interval.

## Conflict of Interest

The authors declare no conflict of interest.

## Supporting information

Supporting Information

## Data Availability

The data that support the findings of this study are available from the corresponding author upon reasonable request.
