# Supplementary material for: Carbon Vacancies Steer the Activity in Dual Ni Carbon Nitride Photocatalysis
Source: Adv Sci (Weinh). 2023 Jul 6;10(26):2303781. doi: 10.1002/advs.202303781 (PMC10502671; doi:10.1002/advs.202303781)
Supplement: Supplementary file 1 — Supporting Information [file ADVS-10-2303781-s001.pdf]

ADVANCED SCIENCE Open Access

Supporting Information

for *Adv. Sci.*, DOI 10.1002/advs.202303781

Carbon Vacancies Steer the Activity in Dual Ni Carbon Nitride Photocatalysis

Miriam Marchi, Edoardo Raciti, Sai Manoj Gali, Federica Piccirilli, Hendrik Vondracek, Arianna Actis, Enrico Salvadori, Cristian Rosso, Alejandro Criado, Carmine D'Agostino, Luke Forster, Daniel Lee, Alexandre C. Foucher, Rajeev Kumar Rai, David Beljonne, Eric A. Stach, Mario Chiesa, Roberto Lazzaroni, Giacomo Filippini, Maurizio Prato, Michele Melchionna* and Paolo Fornasiero**

Supporting Information

Carbon vacancies steer the activity in dual Ni carbon nitride photocatalysis

Miriam Marchi, Edoardo Raciti, Sai Manoj Gali, Federica Piccirilli, Hendrik Vondracek, Arianna Actis, Enrico Salvadori, Cristian Rosso, Alejandro Criado, Carmine D'Agostino, Luke Forster, Daniel Lee, Alexandre C. Foucher, Rajeev Kumar Rai, David Beljonne, Eric A. Stach, Mario Chiesa, Roberto Lazzaroni, Giacomo Filippini, Maurizio Prato, Michele Melchionna*, Paolo Fornasiero**

Supplementary characterization of nanomaterials

The characterization of all nanomaterials is reported in Figure S1 (see also main text).

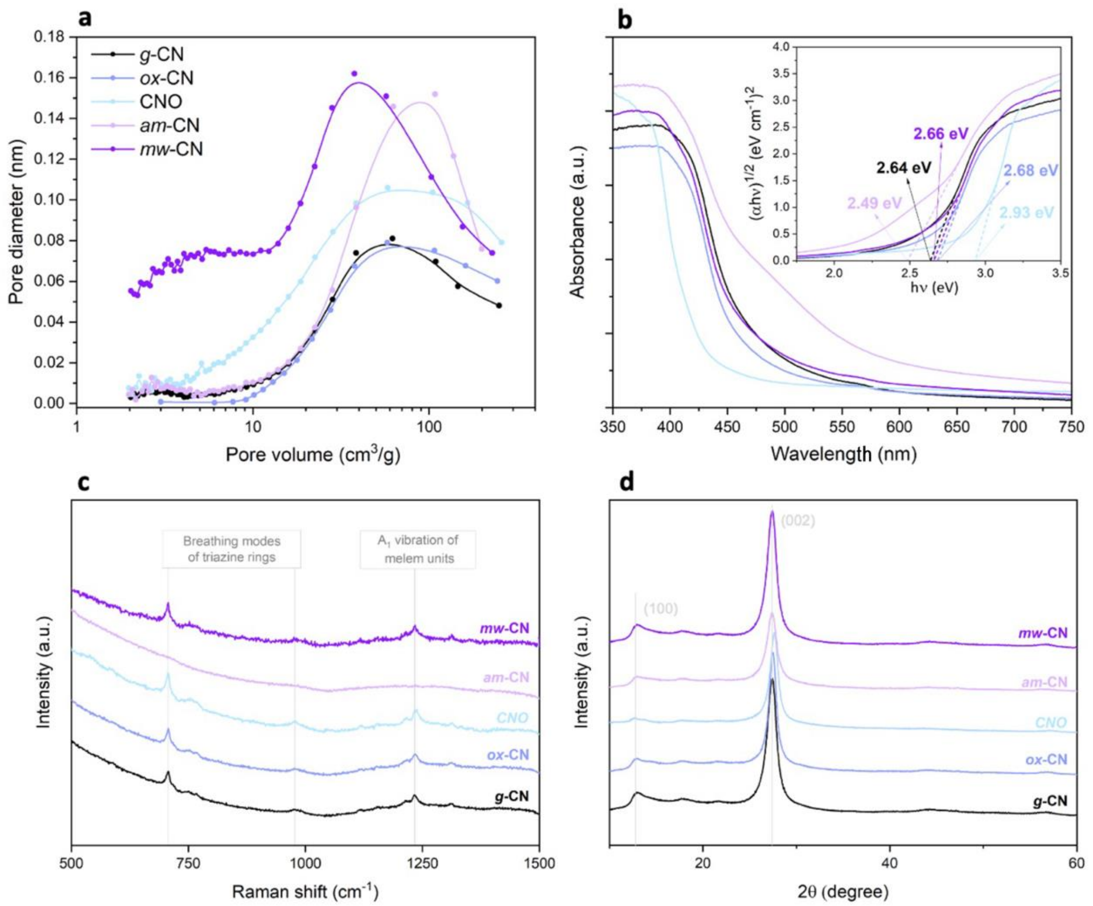

Figure S1. a) BJH pre size BJH pore size distribution; b) UV-Vis DRS spectra and the corresponding band gap (inset) for all samples; c) Raman spectra; d) XRD diffractogram of the four materials.

Table S1. XPS peak assignments, positions and relative areas of *mw*-CN, *g*-CN, CNO, *am*-CN, *ox*-CN.

Sample	Component	B E (eV)	Area (%)
mw-CN	N-N	404.0	9.6
	$N(C)_3$	401.0	9.6
	CN	399.9	16.1
	C=N-C	398.4	64.7
g-CN	N-N	404.4	6.4
	$N(C)_3$	400.8	17.6
	C=N-C	398.7	76.0
CNO	N-N	404.9	6.3
	$N(C)_3$	400.6	19.1
	C=N-C	398.6	74.6
am-CN	N-N	404.4	5.4

	$N(C)_3$	400.9	18.1
	C=N-C	398.8	76.5
ox-CN	N-N	404.9	6.3
	$N(C)_3$	400.6	19.1
	C=N-C	398.6	74.6

Screening of the reaction conditions for C-N, C-O, C-S cross-coupling reactions

The screening of the reaction conditions (Table S2) has been carried out under blue light irradiation (450 nm) with LED strips (Figure S2 (a)).

Table S2. Optimization studies and control experiments. Reactions were performed on 0.1 mmol scale. [a] Yield determined by ^{1}H-NMR spectroscopy using 1-fluoro-2-nitrobenzene as internal standard.

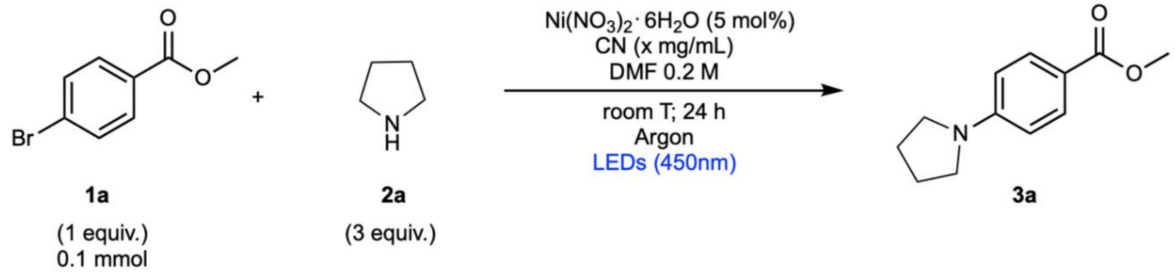

Entry	CN (mg/mL)	Deviation	$Yield_{3a}$ (%) [a]
1	*g*-CN (5)	None	> 99
2	*g*-CN (5)	Dark	0
3	*g*-CN (5)	Air	0
4	None	No *g*-CN	0
5	*g*-CN (5)	No Ni catalyst	0
6	*g*-CN (5)	0.5 mmol	> 99
7	*g*-CN (5)	525 nm	0
8	*g*-CN (5)	Ni catalyst (2.5 mol%)	58 ± 3
9	*g*-CN (5)	Pyrrolidine (2 equiv.)	64 ± 3
10	*g*-CN (2.5)	None	48 ± 5
11	*am*-CN (2.5)	None	7 ± 5
12	CNO (2.5)	None	5 ± 2

13	*ox*-CN (2.5)	None	24 ± 2
14	*mw*-CN (2.5)	None	87 ± 8
15	*mw*-CN (2.5)	Kessil (456 nm)	97 ± 3

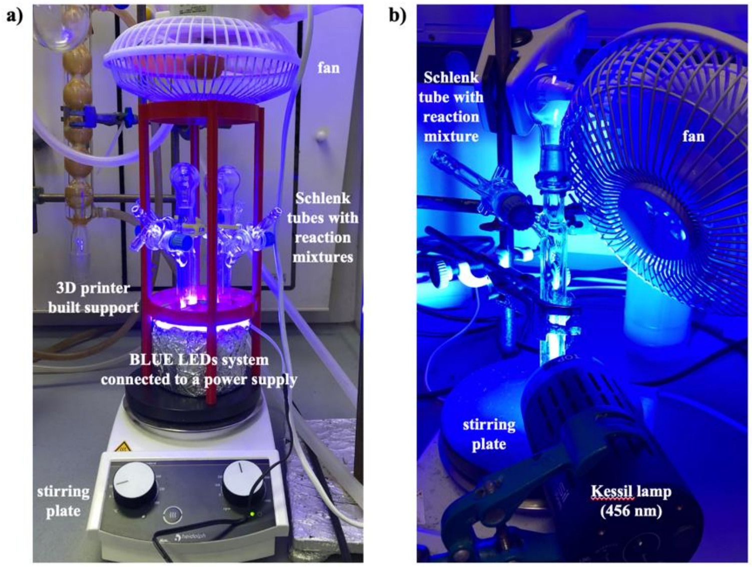

Figure S2. Batch photochemical set-up: (a) LED strips set- up; (b) Kessil lamp set-up.

General procedure for the Ni dual photocatalytic C-N coupling reactions

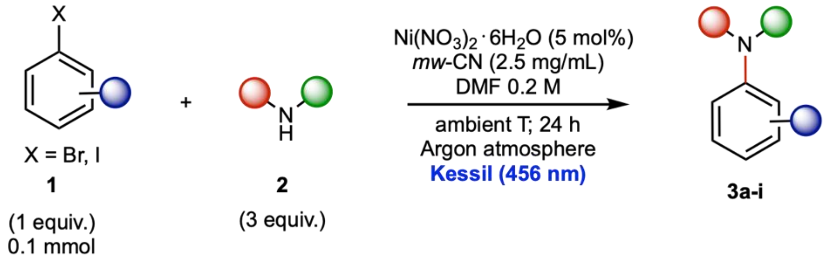

A 10 mL Schlenk tube was charged with the appropriate aryl halide **1** (0.1 mmol, 1 equiv.), the corresponding amine **2** (0.3 mmol, 3 equiv.), the *mw*-CN photocatalyst (2.5 mg/mL), nickel(II) nitrate hexahydrate (5% mol), and DMF ($[\mathbf{1}]_0$ = 0.2 M). The reaction mixture was thoroughly degassed via 4 cycles of freeze-pump-thaw, and the vessel was refilled with argon, sealed with parafilm, and irradiated with blue light (λ = 456 nm, Figure S2 (b)). The temperature was kept at around 30°C by using a fan. Stirring was maintained for 24 hours, then the irradiation was stopped.

The reaction mixture was diluted with aqueous LiCl (5% wt/v) and extracted with ethyl acetate (5 x 10 mL). The combined organic layers were dried over sodium sulfate and the volatiles were removed in vacuo. The residue was purified by column chromatography to give the corresponding aniline products **3** in the stated yield.

Product Characterization Data

Methyl 4-(pyrrolidin-1-yl)benzoate (3a)

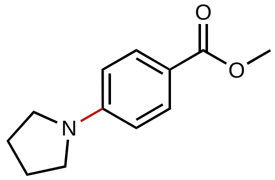

3a was synthesized according to the general procedure **C.1** from methyl 4-bromobenzoate (21.5 mg, 0.1 mmol, 1 equiv.) and pyrrolidine (25.0 µl, 0.3 mmol, 3 equiv.). Reaction time: 24 hours. The final product **3a** was obtained with 90% yield.

^{1}H-NMR (400 MHz, $CDCl_3$) δ 7.90 (d, J = 9.0 Hz, 2H), 6.53 (d, J = 9.0 Hz, 2H), 3.85 (s, 3H), 3.43 – 3.32 (m, 4H), 2.06 – 1.97 (m, 4H)); **^{13}C-NMR (101 MHz, $CDCl_3$)** δ 167.73 (s), 150.85 (s), 131.53 (s), 116.76 (s), 111.03 (s), 51.59 (s), 47.89 (s), 25.58 (s); **HRMS (ESI, positive mode)** calculated for $C_{12}H_{15}NO_2$ $[M+Na]^+$: 228.0994, found: 228.0995.

Methyl (*R*)-4-(3-hydroxypyrrolidin-1-yl)benzoate (3b)

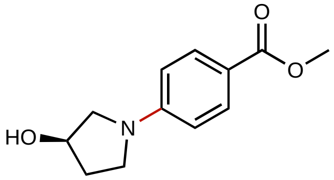

3b was synthesized according to the general procedure **C.1** from methyl 4-bromobenzoate (21.5 mg, 0.1 mmol, 1 equiv.), (R)-3-pyrrolidinol (37.07 mg, 0.3 mmol, 3 equiv.) and DABCO (44.87 mg, 0.4 mmol, 4 equiv.). Reaction time: 24 hours. The final product **3b** was obtained with 72% yield.

^{1}H-NMR (400 MHz, $CDCl_3$) δ 7.89 (d, *J* = 7.4 Hz, 2H), 6.49 (d, *J* = 7.0 Hz, 2H), 4.63 (s, 1H), 3.85 (s, 3H), 3.55 (m, *J* = 8.1, 7.6 Hz, 2H), 3.48 – 3.39 (m, 1H), 3.37 – 3.30 (m, 1H), 2.29 – 1.85 (m, 3H); **^{13}C-NMR (101 MHz, $CDCl_3$)** δ 167.81 (s), 150.91 (s), 131.53 (s), 116.96 (s), 110.93 (s), 71.14 (s), 56.15 (s), 51.64 (s), 45.57 (s), 34.20 (s); **HRMS (ESI, positive mode)** calculated for $C_{12}H_{15}NO_3$ $[M+Na]^+$: 244.0944, found: 244.0945.

Methyl 4-(3,4-dihydroisoquinolin-2(1H)-yl)benzoate (3c)

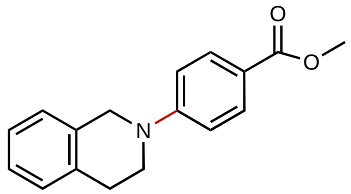

3c was synthesized according to the general procedure **C.1** from methyl 4-bromobenzoate (21.5 mg, 0.1 mmol, 1 equiv.), 1,2,3,4-tetrahydroisoquinoline (37.5 µl, 0.3 mmol, 3 equiv.). Reaction time: 24 hours. The final product **3c** was obtained with 60% yield.

^{1}H-NMR (400 MHz, $CDCl_3$) δ 7.95 (d, J = 9.1 Hz, 1H), 7.25 – 7.14 (m, 4H), 6.88 (d, J = 9.1 Hz, 1H), 4.52 (s, 2H), 3.87 (s, 3H), 3.66 (t, J = 5.9 Hz, 2H), 3.00 (t, J = 5.9 Hz, 2H); **^{13}C-NMR (101 MHz, $CDCl_3$)** δ 167.45 (s), 153.14 (s), 135.16 (s), 133.94 (s), 131.48 (s), 128.35 (s), 126.85 (s), 126.63 (s), 126.50 (s), 118.42 (s), 112.21 (s), 51.71 (s), 49.18 (s), 44.93 (s), 29.18 (s); **HRMS (ESI, positive mode)** calculated for $C_{17}H_{17}NO_2$ $[M+Na]^+$: 268.1332, found: 268.1330.

Methyl 4-morpholinobenzoate (3d)

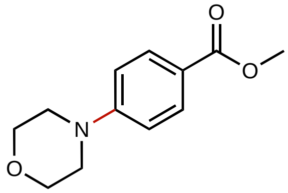

3d was synthesized according to the general procedure **C.1** from methyl 4-bromobenzoate (21.5 mg, 0.1 mmol, 1 equiv.) and morpholine (26.1 µl, 0.3 mmol, 3 equiv.). Reaction time: 24 hours. The final product **3d** was obtained with 30% yield.

^{1}H-NMR (400 MHz, $CDCl_3$) δ 7.94 (d, J = 9.1 Hz, 2H), 6.86 (d, J = 9.1 Hz, 2H), 3.89 – 3.82 (m, 7H), 3.28 (t, 4H); **^{13}C-NMR (101 MHz, $CDCl_3$)** δ 167.05 (s), 154.19 (s), 131.21 (s), 120.32 (s), 113.47 (s), 66.60 (s), 51.69 (s), 47.71 (s); **HRMS (ESI, positive mode)** calculated for $C_{12}H_{15}NO_3$ $[M+Na]^+$: 244.0944, found: 244.0944.

Methyl 4-(piperidin-1-yl)benzoate (3e)

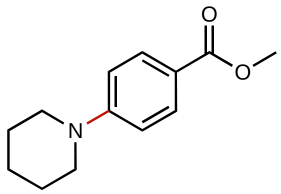

3e was synthesized according to the general procedure **C.1** from methyl 4-bromobenzoate (21.5 mg, 0.1 mmol, 1 equiv.) and piperidine (29.6 µl, 0.3 mmol, 3 equiv.). Reaction time: 24 hours. The final product **3e** was obtained with 48% yield.

^{1}H-NMR (400 MHz, $CDCl_3$) δ 7.89 (d, J = 9.2 Hz, 2H), 6.85 (d, J = 9.1 Hz, 2H), 3.85 (s, 3H), 3.36 – 3.28 (m, 4H), 1.66 (m, 6H); **^{13}C-NMR (101 MHz, $CDCl_3$)** δ 167.39 (s), 154.65 (s), 131.36 (s), 118.79 (s), 113.70 (s), 51.69 (s), 48.92 (s), 25.52 (s), 24.49 (s); **HRMS (ESI, positive mode)** calculated for $C_{13}H_{17}NO_2$ $[M+Na]^+$: 242.1151, found: 242.1151.

Methyl 4-(butylamino)benzoate (3f)

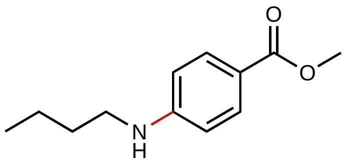

3f was synthesized according to the general procedure **C.1** from methyl 4-iodobenzoate (26.2 mg, 0.1 mmol, 1 equiv.) and butylamine (29.7 µl, 0.3 mmol, 3 equiv.). Reaction time: 24 hours. The final product **3f** was obtained with 48% yield.

^{1}H-NMR (400 MHz, CDCl$_3$) δ 7.85 (d, J = 8.9 Hz, 2H), 6.53 (d, J = 8.9 Hz, 2H), 4.09 (s, 1H), 3.84 (s, 2H), 3.16 (t, J = 7.0 Hz, 2H), 1.61 (m, J = 14.6, 7.3 Hz, 2H), 1.43 (m, J = 14.4, 7.3 Hz, 2H), 0.96 (t, J = 7.3 Hz, 3H); **^{13}C-NMR (101 MHz, CDCl$_3$)** δ 167.51 (s), 152.28 (s), 131.68 (s), 118.13 (s), 111.43 (s), 51.63 (s), 43.19 (s), 31.54 (s), 20.34 (s), 13.98 (s); **HRMS (ESI, positive mode)** calculated for $C_{12}H_{17}NO_2$ $[M+Na]^+$: 230.1151, found: 230.1152.

1-(4-(Trifluoromethyl)phenyl)pyrrolidine (3g)

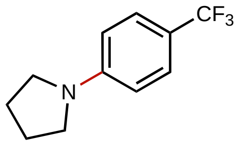

3g was synthesized according to the general procedure **C.1** from 4-bromobenzotrifluoride (14.0 µl, 0.1 mmol, 1 equiv.) and pyrrolidine (25.0 µl, 0.3 mmol, 3 equiv.). Reaction time: 24 hours. The final product **3g** was obtained with 88% yield.

^{1}H-NMR (400 MHz, CDCl$_3$) δ 7.44 (d, J = 8.5 Hz, 2H), 6.55 (d, J = 8.7 Hz, 2H), 3.36 – 3.29 (m, 4H), 2.07 – 2.00 (m, 4H); **^{19}F NMR (376 MHz, CDCl$_3$)** δ -60.62 (s, 3F). **^{13}C-NMR (101 MHz, CDCl$_3$)** δ 150.14 (s), 133.57 (s), 121.14 (s), 111.57 (s), 96.66 (s), 47.60 (s), 25.53 (s); **HRMS (ESI, positive mode)** calculated for $C_{11}H_{12}F_3N$ $[M+H]^+$: 216.0995, found: 216.0997.

4-Pyrrolidin-1-yl-benzonitrile (3h)

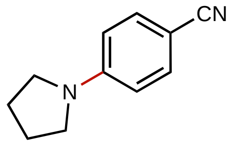

3h was synthesized according to the general procedure **C.1** from 4-bromobenzonitrile (18.2 mg, 0.1 mmol, 1 equiv.) and pyrrolidine (25.0 µl, 0.3 mmol, 3 equiv.). Reaction time: 24 hours. The final product **3h** was obtained with 73% yield.

^{1}H-NMR (400 MHz, CDCl$_3$) δ 7.44 (d, J = 9.0 Hz, 2H), 6.49 (d, J = 9.0 Hz, 2H), 3.36 – 3.29 (m, 4H), 2.08 – 2.01 (m, 4H); **^{13}C-NMR (101 MHz, CDCl$_3$)** δ 150.14 (s), 133.57 (s), 121.14 (s), 111.57 (s), 96.66 (s), 47.60 (s), 25.53; **HRMS (ESI, positive mode)** calculated for $C_{11}H_{12}N_2$ $[M+Na]^+$: 195.0893, found: 195.0890.

1-(3,5-Bis(trifluoromethyl)phenyl)pyrrolidine (3i)

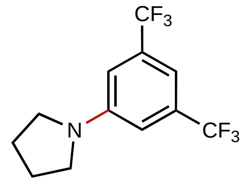

3i was synthesized according to the general procedure **C.1** from 1-bromo-3,5-bis(trifluoromethyl)benzene (17.3 µl, 0.1 mmol, 1 equiv.) and pyrrolidine (25.0 µl, 0.3 mmol, 3 equiv.). Reaction time: 24 hours. The final product **3i** was obtained with 69% yield.

^{1}H-NMR (400 MHz, $CDCl_3$) δ 7.08 (s, 1H), 6.85 (s, 2H), 3.38 – 3.29 (m, 4H), 2.10 – 2.03 (m, 4H); **^{19}F NMR (376 MHz, $CDCl_3$)** δ -63.09 (s, 6F); **^{13}C-NMR (101 MHz, $CDCl_3$)** δ 148.03 (s), 132.26 (d, J = 32.4 Hz), 123.99 (d, J = 272.6 Hz), 110.89 (s), 108.08 (s), 47.90 (s), 25.60 (s)); **HRMS (ESI, positive mode)** calculated for $C_{12}H_{11}F_6N$ $[M+H]^+$: 284.0868, found: 284.0866.

General procedure for the Ni dual photocatalytic C-O and C-S coupling reactions

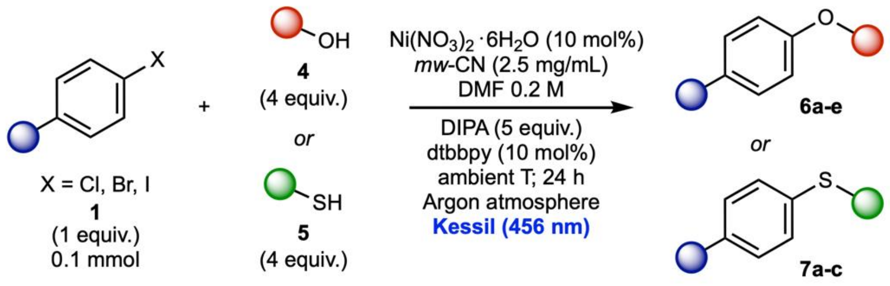

A 10 mL Schlenk tube was charged with the appropriate aryl halide **1** (0.1 mmol, 1 equiv.), the corresponding alcohol **4** or thiol **5** (0.4 mmol, 4 equiv.), the *mw*-CN photocatalyst (2.5 mg/mL), nickel(II) nitrate hexahydrate (10 mol%), diisopropyl amine (0.5 mmol, 5 equiv.), 4,4'-di-tert-butyl-2,2'-dipyridyl (dtbbpy, 0.01 mmol, 10 mol%), and DMF ($[\mathbf{1}]_0$ = 0.2 M). The reaction mixture was thoroughly degassed via 4 cycles of freeze-pump-thaw, and the vessel was refilled with argon, sealed with parafilm, and irradiated with blue light (λ = 456 nm, Figure S2 (b)). The temperature was kept at around 30°C by using a fan. Stirring was maintained for 24 hours, then the irradiation was stopped. The reaction mixture was diluted with aqueous LiCl (5% wt/v) and extracted with ethyl acetate (5 x 10 mL). The combined organic layers were dried over sodium sulfate and the volatiles were removed in vacuo. The residue was purified by column chromatography to give the corresponding aniline products **6** or **7** in the stated yield.

Product Characterization Data

Methyl 4-hydroxybenzoate (6a)

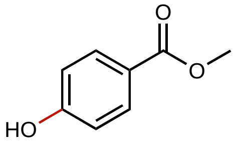

6a was synthesized according to the general procedure **C.2** from methyl 4-bromobenzoate (21.5 ml, 0.1 mmol, 1 equiv.) / methyl 4-iodobenzoate (26.2 mg, 0.1 mmol, 1 equiv.) and water (7.2 µl, 0.4 mmol, 4 equiv.). Reaction time: 24 hours. The final product **6a** was obtained with 79% and 80% yield respectively.

^{1}H-NMR (400 MHz, $CDCl_3$) δ 7.96 (s, 1H), 6.90 (s, 1H), 3.90 (s, 3H); **^{13}C-NMR (101 MHz, $CDCl_3$)** δ 167.66 (s), 160.47 (s), 132.12 (s), 122.37 (s), 115.45 (s), 52.28 (s); **HRMS (ESI, positive mode)** calculated for $C_8H_8O_3$ $[M+Na]^+$: 175.0367, found: 175.0366.

4-Cyanophenol (6b)

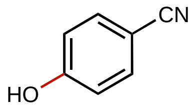

6b was synthesized according to the general procedure **C.2** from methyl 4-chlorobenzonitrile (13.8 mg, 0.1 mmol, 1 equiv.) / methyl 4-bromobenzonitrile (18.2 mg, 0.1 mmol, 1 equiv.) / methyl 4-iodobenzonitrile (22.9 mg, 0.1 mmol, 1 equiv.) and water (7.2 µl, 0.4 mmol, 4 equiv.). Reaction time: 24 hours. The final product **6b** was obtained with 28%, 73% and 72% yield respectively.

^{1}H-NMR (400 MHz, CDCl3) δ 7.55 (d, J = 8.8 Hz, 2H), 6.93 (d, J = 8.6 Hz, 2H); **^{13}C-NMR (101 MHz, CDCl3)** δ 160.11 (s), 134.31 (s), 119.24 (s), 116.44 (s), 103.22 (s); **HRMS (ESI, positive mode)** calculated for C_7H_5NO $[M+Na]^+$: 142.0263, found: 142.0261.

4-Methoxymethylbenzoate (6c)

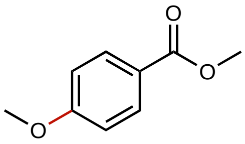

6c was synthesized according to the general procedure **C.2** from 4-bromobenzoate (21.5 mg, 0.1 mmol, 1 equiv.) / methyl 4-iodobenzonitrile (22.9 mg, 0.1 mmol, 1 equiv.) and methanol (16 µl, 0.4 mmol, 4 equiv.). Reaction time: 24 hours. The final product **6c** was obtained with 60% and 67% yield respectively.

^{1}H-NMR (400 MHz, $CDCl_3$) δ 8.00 (d, 2H), 6.92 (d, 2 H), 3.88 (s, 3H), 3.86 (s, 3H); **^{13}C-NMR (101 MHz, $CDCl_3$)** δ 167.01 (s), 163.47 (s), 131.74 (s), 122.76 (s), 113.74 (s), 55.58 (s), 52.02 (s); **HRMS (ESI, positive mode)** calculated for $C_9H_{10}O_3$ $[M+Na]^+$: 189.0522, found: 189.0497.

4-Benzyloxy-benzoic acid methyl ester (6d)

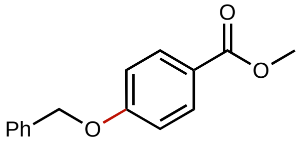

6d was synthesized according to the general procedure **C.2** from 4-bromobenzoate (21.5 mg, 0.1 mmol, 1 equiv.) / methyl 4-iodobenzoate (22.9 mg, 0.1 mmol, 1 equiv.) and benzyl alcohol (42 µl, 0.4 mmol, 4 equiv.). Reaction time: 24 hours. The final product **6d** was obtained with 66% and 59% yield respectively.

^{1}H-NMR (400 MHz, CDCl$_3$) δ 8.00 (d, *J* = 8.8 Hz, 2H), 7.45 – 7.32 (m, 5H), 7.00 (d, *J* = 8.8 Hz, 2H), 5.12 (s, 2H), 3.89 (s, 3H)); **^{13}C-NMR (101 MHz, CDCl$_3$)** δ 166.95 (s), 162.62 (s), 136.39 (s), 131.74 (s), 128.81 (s), 128.34 (s), 127.62 (s), 122.98 (s), 114.59 (s), 70.23 (s), 52.00 (s); **HRMS (ESI, positive mode)** calculated for $C_{13}H_{14}O_3$ $[M+Na]^+$: 265.0835, found: 265.0837.

Methyl 4-cyclohexyloxybenzoate (6e)

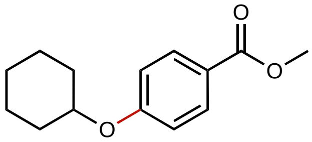

6e was synthesized according to the general procedure **C.2** from 4-bromobenzoate (21.5 mg, 0.1 mmol, 1 equiv.) and cyclohexanol (42 µl, 0.4 mmol, 4 equiv.). Reaction time: 24 hours. The final product **6e** was obtained with 45% yield.

^{1}H-NMR (400 MHz, CDCl$_3$) δ 7.96 (d, *J* = 9.0 Hz, 2H), 6.89 (d, *J* = 8.9 Hz, 2H), 4.36 – 4.30 (m, *J* = 8.9, 4.4 Hz, 1H), 3.87 (s, 3H), 2.03 – 1.94 (m, 2H), 1.86 – 1.77 (m, 2H), 1.63 – 1.48 (m, 3H), 1.46 – 1.23 (m, 3H); **^{13}C-NMR (101 MHz, CDCl$_3$)** δ 167.07 (s), 161.90 (s), 131.71 (s), 122.21 (s), 115.26 (s), 75.53 (s), 51.94 (s), 31.75 (s), 25.66 (s), 23.79 (s); **HRMS (ESI, positive mode)** calculated for $C_{14}H_{18}O_3$ $[M+Na]^+$: 257.1148, found: 257.1149.

Methyl 4-dodecylthiobenzoate (7a)

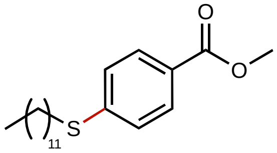

7a was synthesized according to the general procedure **C.2** from methyl 4-iodobenzoate (22.9 mg, 0.1 mmol, 1 equiv.) andb1-dodecanthiol (96 µl, 0.4 mmol, 4 equiv.). Reaction time: 24 hours. The final product **6e** was obtained with 51% yield.

^{1}H-NMR (400 MHz, CDCl$_3$) δ 7.92 (d, J= 8.5 Hz, 2H), 7.28 (d, J= 8.5 Hz, 2H), 3.89 (s, 3H), 2.99 – 2.96 (m, 2H), 1.72 – 1.66 (m, 2H), 1.47 99 – 1.41 (m, 2H), 1.31 – 1.26 (m, 18H), 0.88 (t, 3H); **^{13}C-NMR (101 MHz, CDCl$_3$)** δ 166.83 (s), 144.53 (s), 129.87 (s), 126.51 (s), 126.26 (s), 51.99

(s), 32.07 (s), 31.90 (s) 29.62 (s), 29.61 (s), 29.55 (s), 29.46 (s), 29.33 (s), 29.13 (s), 28.88 (s), 28.74 (s), 22.68 (s), 14.10 (s), 1.01 (s); **HRMS (ESI, positive mode)** calculated for $C_{20}H_{32}O_2S$ $[M+Na]^+$: 359.2015, found: 359.2015.

Methyl 4-(phenethylthio)benzoate (7b)

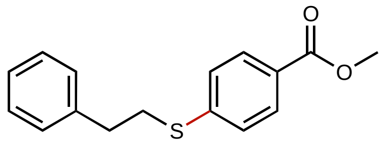

7b was synthesized according to the general procedure **C.2** from 4-iodobenzoate (22.9 mg, 0.1 mmol, 1 equiv.) and benzeneethanethiol (54 µl, 0.4 mmol, 4 equiv.). Reaction time: 24 hours. The final product **7b** was obtained with 55%.

^{1}H-NMR (400 MHz, $CDCl_3$) δ 7.94 (d, J = 8.4 Hz, 2H), 7.27 (m, J = 35.7, 8.4 Hz, 7H), 3.91 (s, 3H), 3.29 – 3.20 (t, 2H), 3.04 – 2.94 (t, 2H); **^{13}C-NMR (101 MHz, $CDCl_3$)** δ 166.91, 143.89, 139.88, 130.11, 128.75, 128.63, 126.99, 126.81, 126.72, 52.18, 35.32, 33.74; **HRMS (ESI, positive mode)** calculated for $C_{16}H_{16}O_2S$ $[M+Na]^+$: 295.0763, found: 295,0762.

Methyl 4-(cyclohexylthio)benzoate (7c)

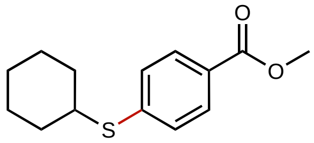

7c was synthesized according to the general procedure **C.2** from 4-iodobenzoate (22.9 mg, 0.1 mmol, 1 equiv.) and cyclohexanethiol (49 µl, 0.4 mmol, 4 equiv.). Reaction time: 24 hours. The final product **7c** was obtained with 50% yield.

^{1}H-NMR (400 MHz, $CDCl_3$) δ 7.92 (d, J = 8.5 Hz, 2H), 7.34 (d, J = 8.5 Hz, 2H), 3.89 (s, 3H), 3.32 – 3.18 (m, 1H), 2.12 – 1.99 (m, 2H), 1.81 – 1.79 (m, 2H), 1.71 – 1.62 (m, 1H), 1.46 – 1.30 (m, 5H); **^{13}C-NMR (101 MHz, $CDCl_3$)** δ 166.95 (s), 143.16 (s), 130.01 (s), 128.65 (s), 127.26 (s), 52.17 (s), 45.23 (s), 33.24 (s), 26.08 (s), 25.83 (s); **HRMS (ESI, positive mode)** calculated for $C_{14}H_{18}O_2S$ $[M+Na]^+$: 273.0919, found: 273.0920.

Methyl 4-(pentylthio)benzoate (7d)

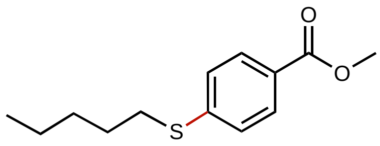

7d was synthesized according to the general procedure **C.2** from 4-iodobenzoate (22.9 mg, 0.1 mmol, 1 equiv.) and 1-pentanethiol (50 µl, 0.4 mmol, 4 equiv.). Reaction time: 24 hours. The final product **7d** was obtained with 83% yield.

^{1}H-NMR (400 MHz, $CDCl_3$) δ 7.89 (d, *J* = 8.6 Hz, 2H), 7.25 (d, *J* = 8.6 Hz, 2H), 3.87 (s, 3H), 2.97 – 2.92 (m, 2H), 1.67 (m, 2H), 1.41 (m, 2H), 1.32 (m, 2H), 0.88 (t, 3H)); **^{13}C-NMR (101 MHz, $CDCl_3$)** δ 166.96 (s), 144.65 (s), 130.01 (s), 126.65 (s), 126.40 (s), 52.13 (s), 32.17 (s), 31.17 (s), 28.57 (s), 22.35 (s), 14.05 (s).

Solution NMR spectra

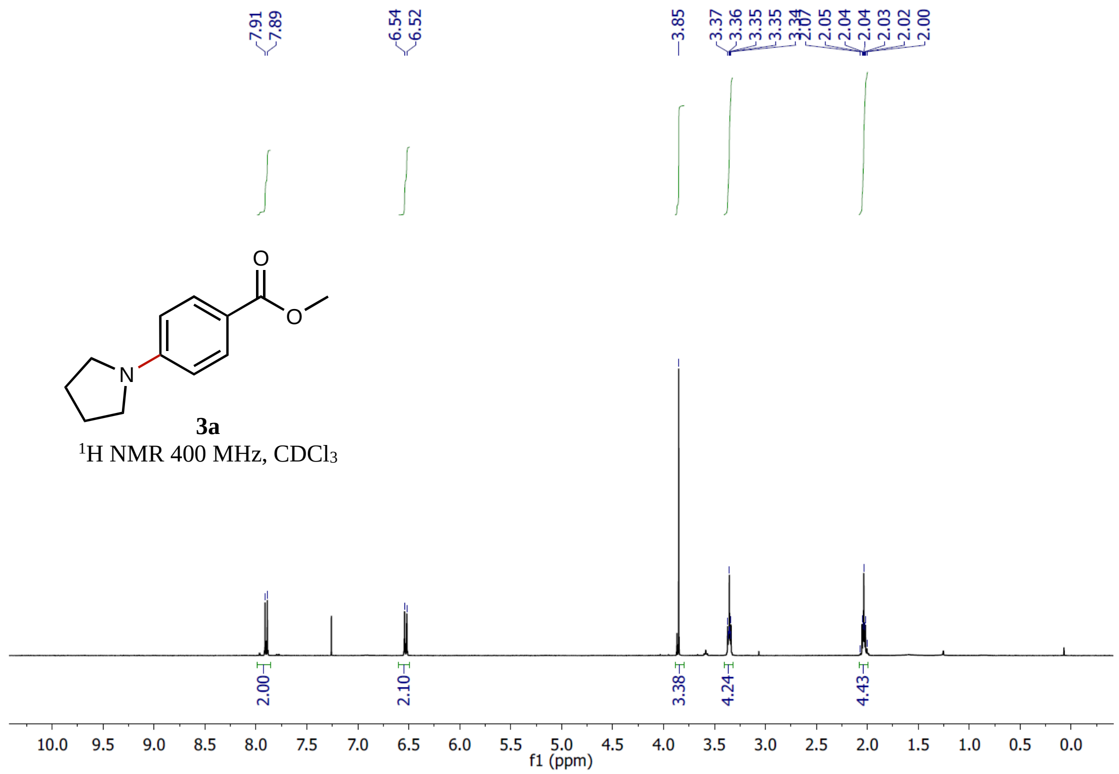

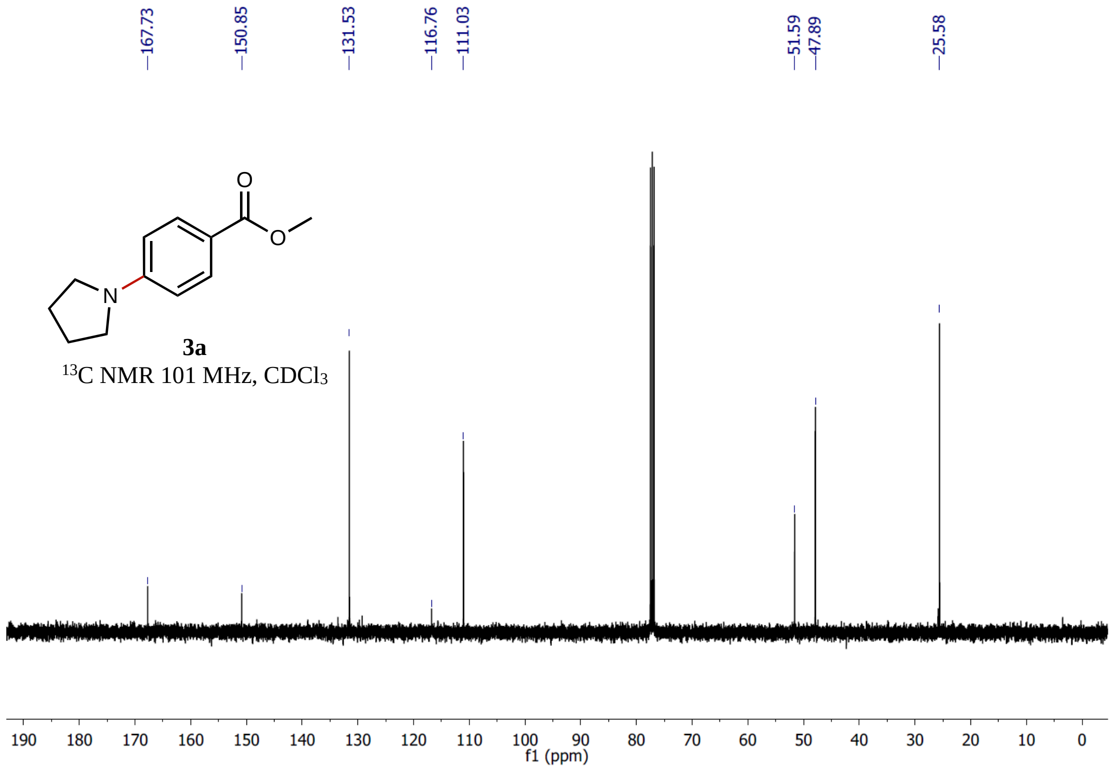
167.73
150.85
131.53
116.76
111.03
51.59
47.89
25.58
3a
^{13}C NMR 101 MHz, $CDCl_3$
f1 (ppm)

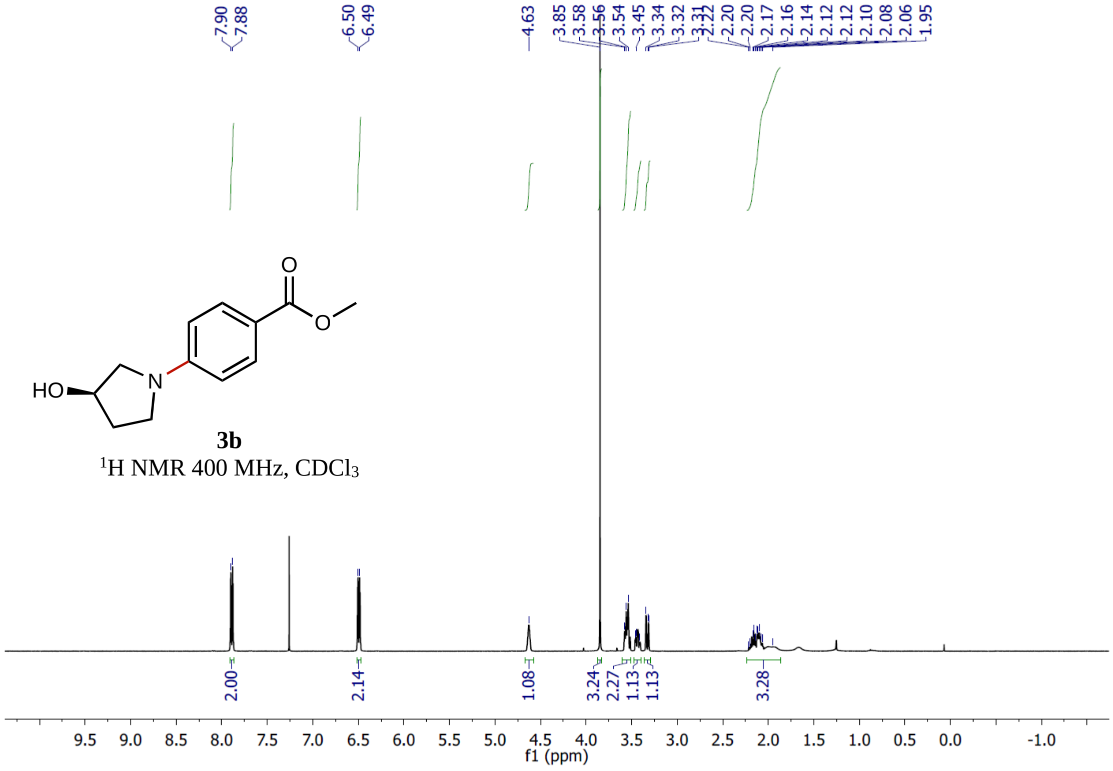
7.90
7.88
6.50
6.49
4.63
3.85
3.58
3.56
3.54
3.45
3.34
3.32
3.31
3.22
2.20
2.20
2.17
2.16
2.14
2.12
2.10
2.08
2.06
1.95
2.00
2.14
1.08
3.24
2.27
1.13
1.13
3.28
HO
3b
^{1}H NMR 400 MHz, $CDCl_3$
f1 (ppm)

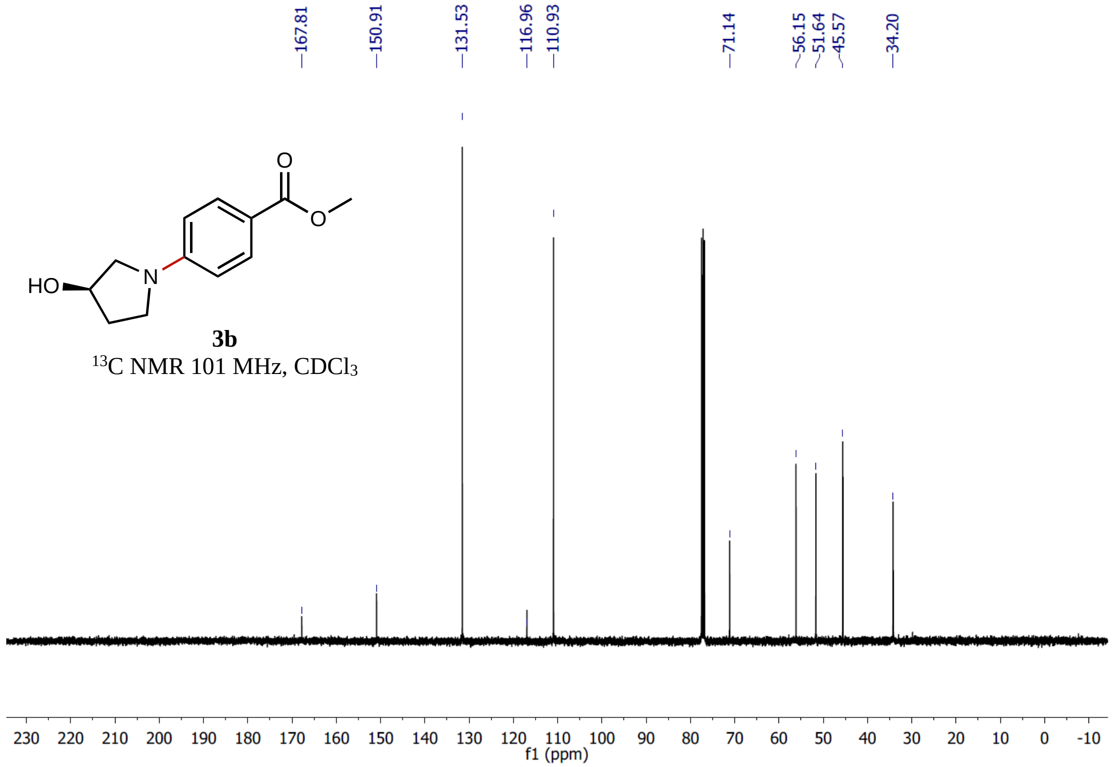

3b
^{13}C NMR 101 MHz, $CDCl_3$

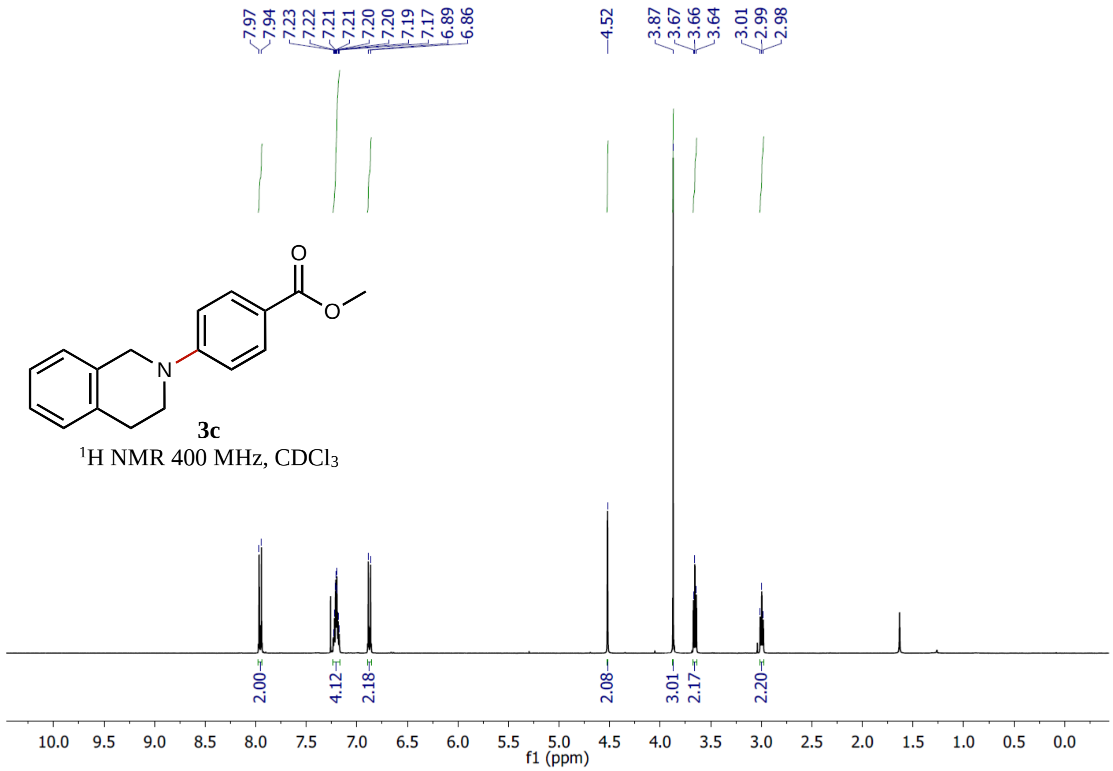

3c
^{1}H NMR 400 MHz, $CDCl_3$

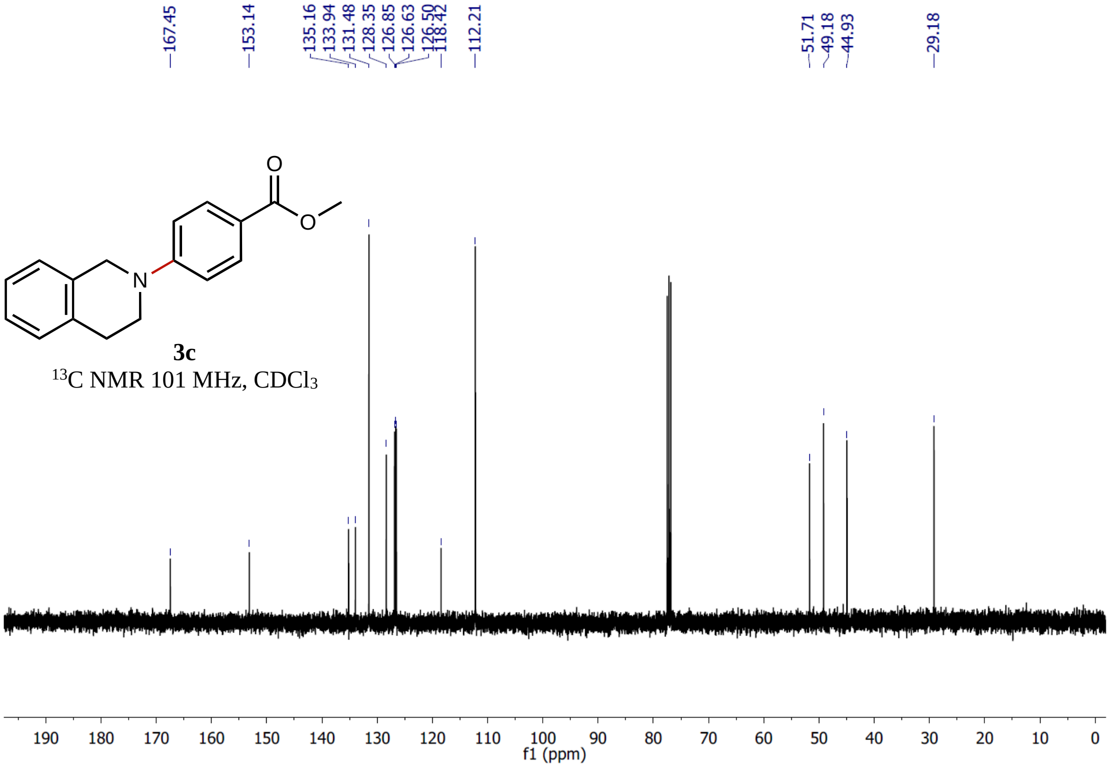

3c

^{13}C NMR 101 MHz, $CDCl_3$

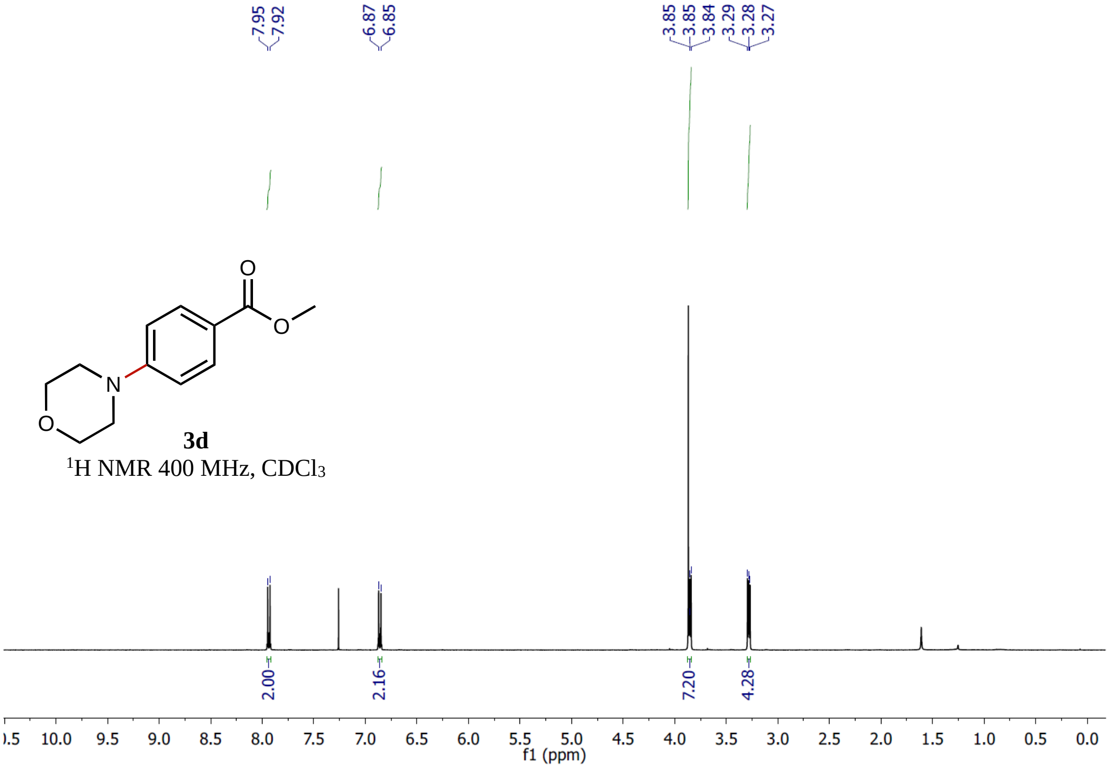

3d

^{1}H NMR 400 MHz, $CDCl_3$

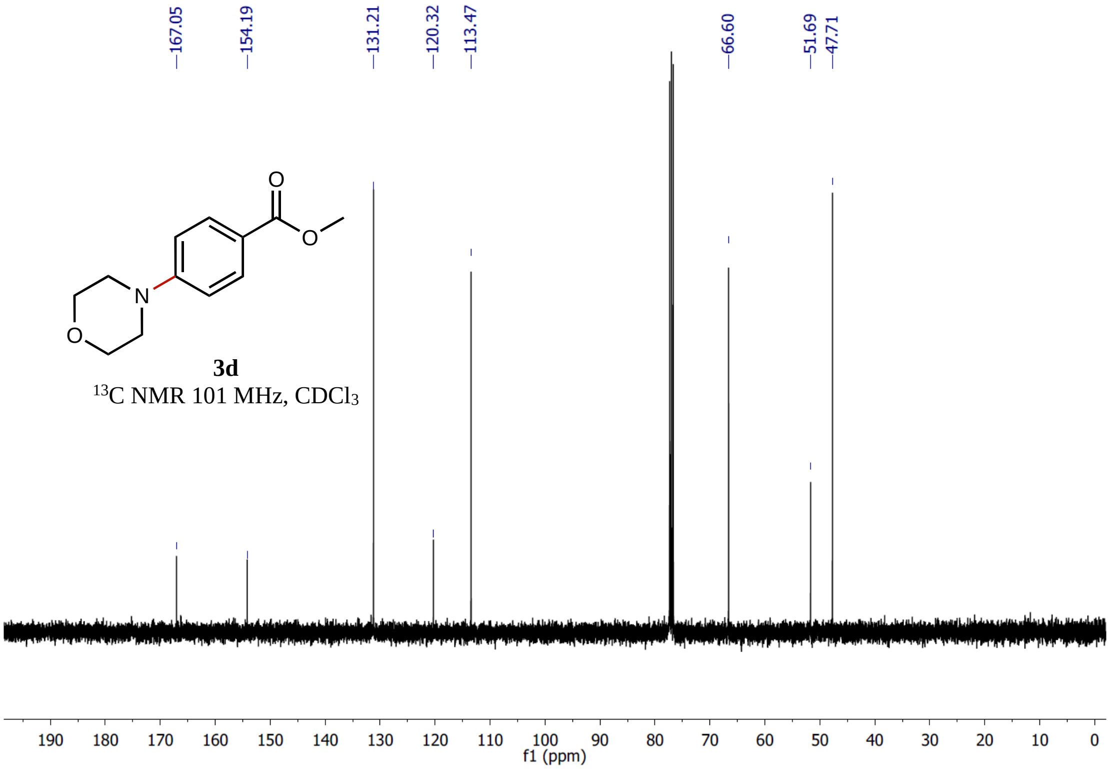

167.05
154.19
131.21
120.32
113.47
66.60
51.69
47.71
O
O
N
O
3d
13C NMR 101 MHz, CDCl3
190
180
170
160
150
140
130
120
110
100
90
80
70
60
50
40
30
20
10
0
f1 (ppm)

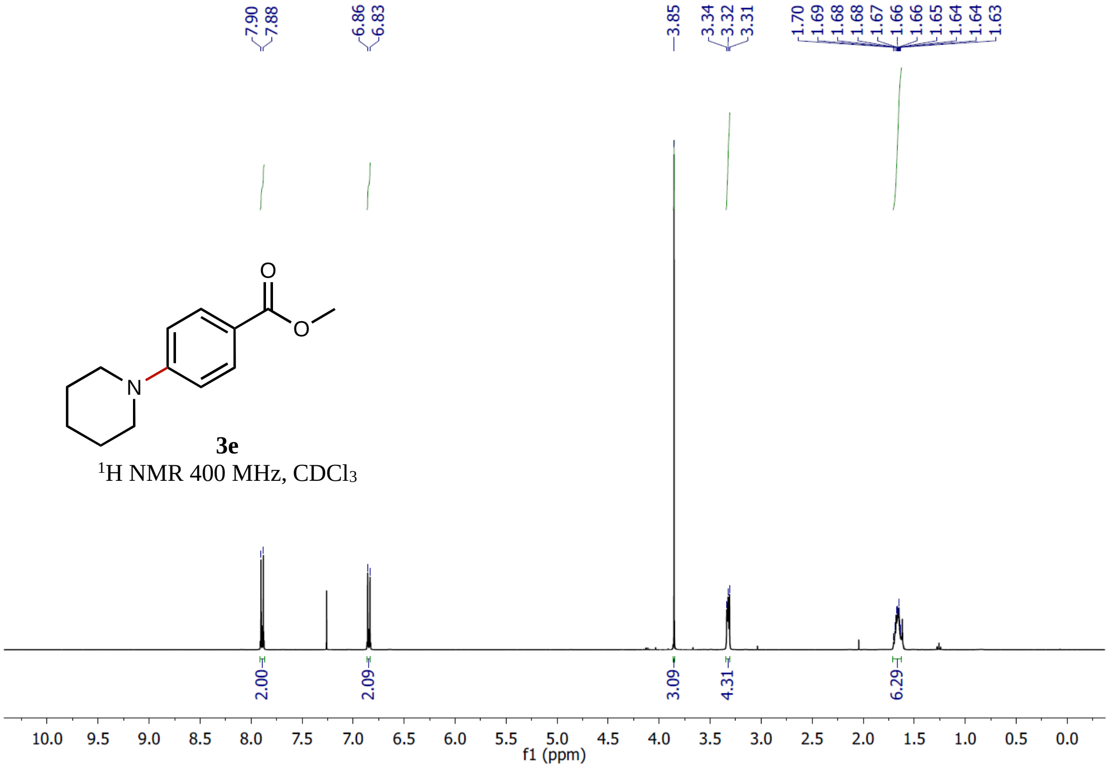

7.90
7.88
6.86
6.83
3.85
3.34
3.32
3.31
1.70
1.69
1.68
1.68
1.67
1.66
1.66
1.65
1.64
1.64
1.63
O
O
N
3e
1H NMR 400 MHz, CDCl3
2.00
2.09
3.09
4.31
6.29
10.0
9.5
9.0
8.5
8.0
7.5
7.0
6.5
6.0
5.5
5.0
4.5
4.0
3.5
3.0
2.5
2.0
1.5
1.0
0.5
0.0
f1 (ppm)

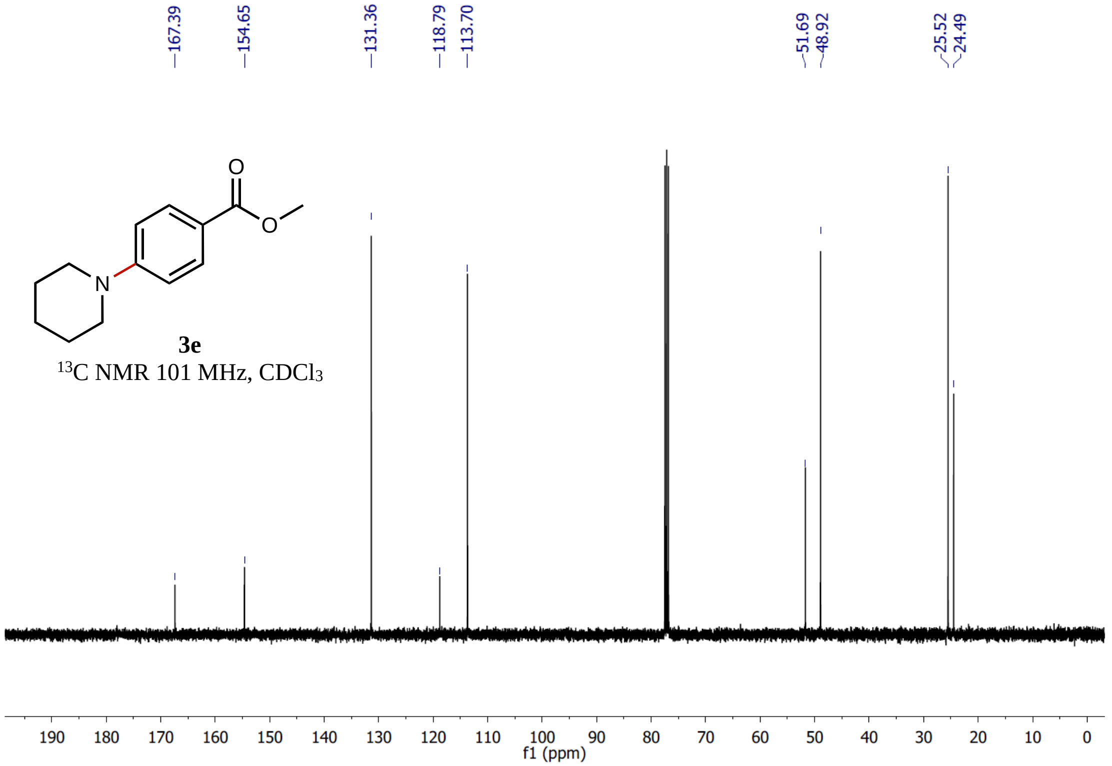

3e
^{13}C NMR 101 MHz, $CDCl_3$

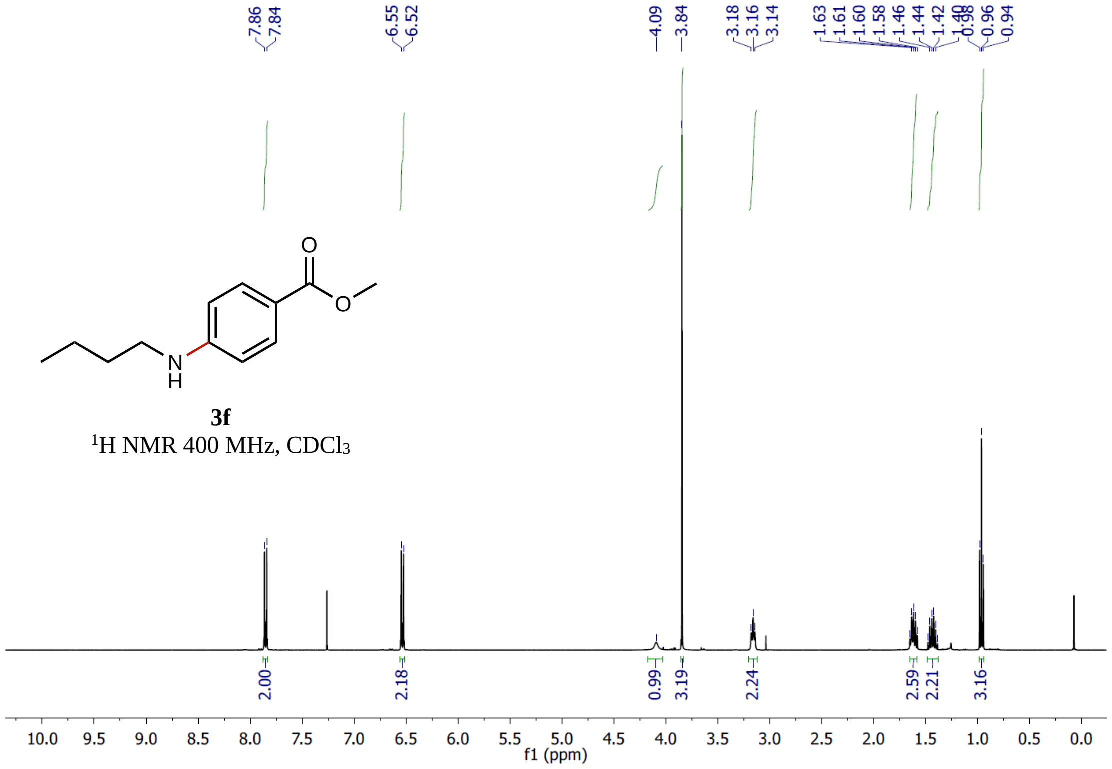

3f
^{1}H NMR 400 MHz, $CDCl_3$

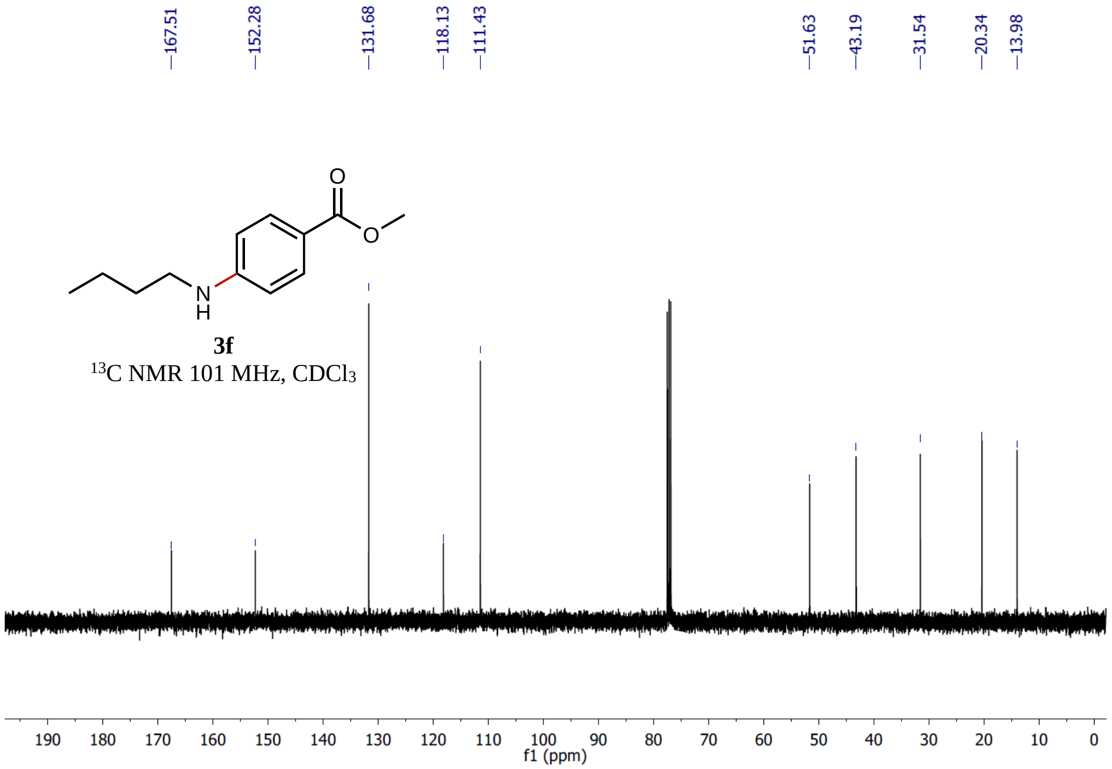

3f

^{13}C NMR 101 MHz, $CDCl_3$

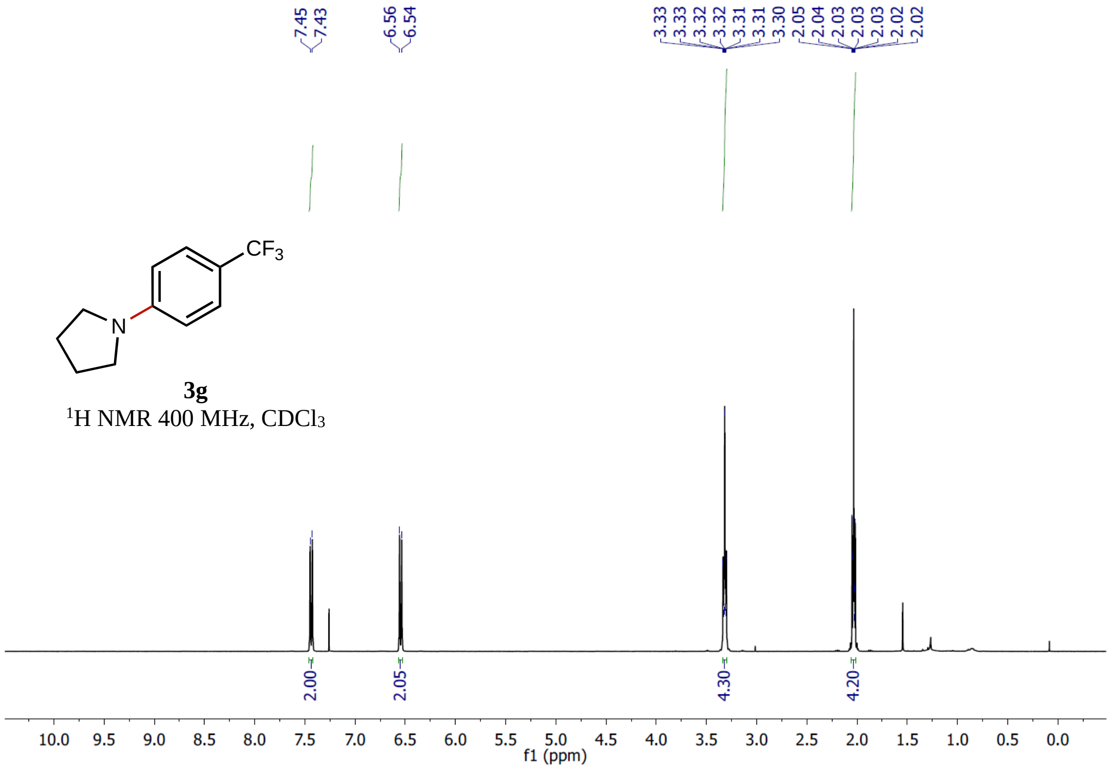

3g

^{1}H NMR 400 MHz, $CDCl_3$

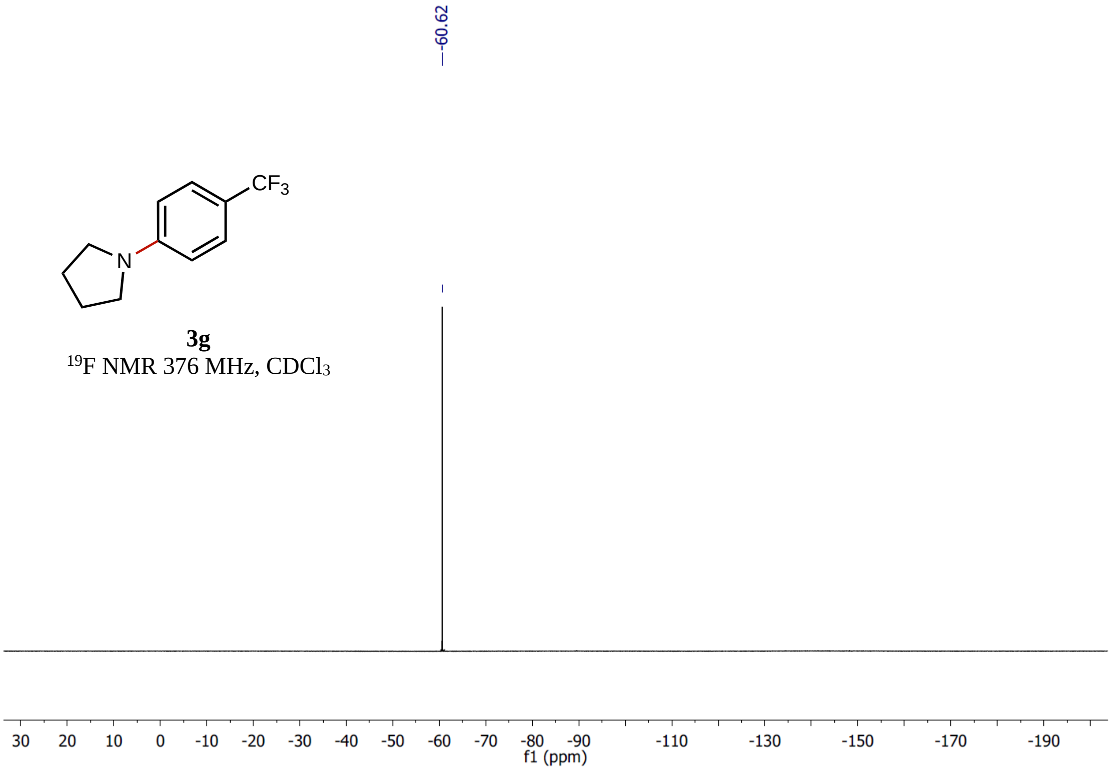

3g
^{19}F NMR 376 MHz, $CDCl_3$

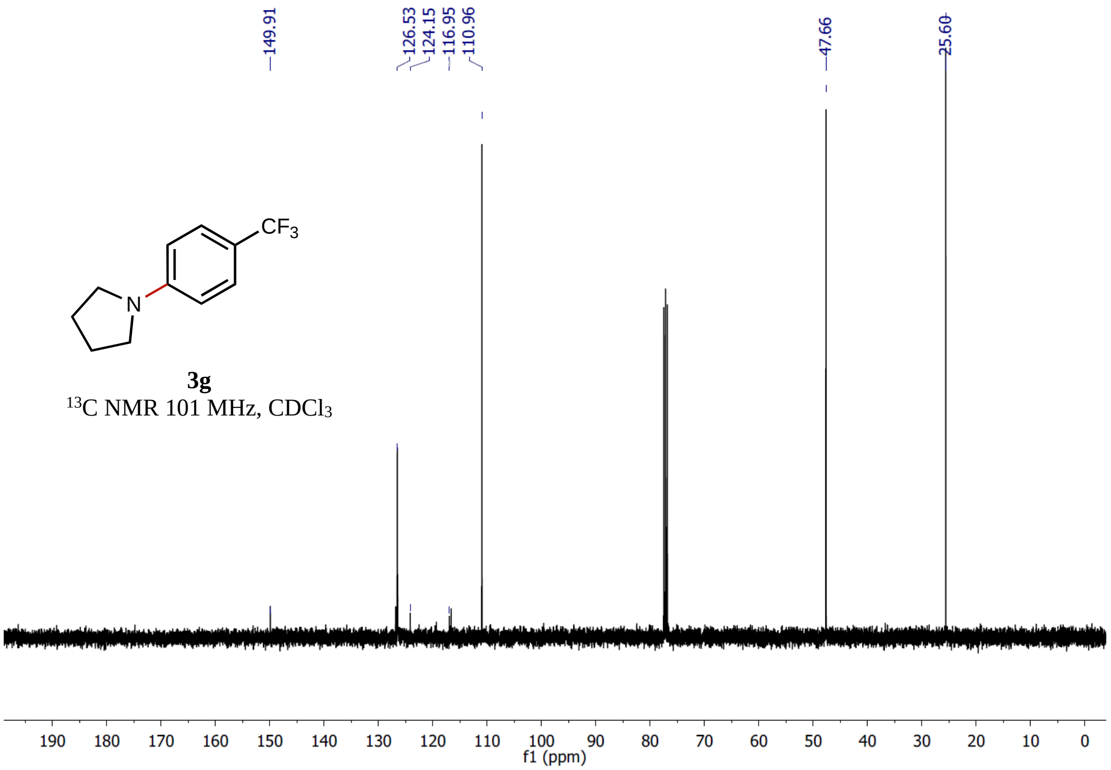

3g
^{13}C NMR 101 MHz, $CDCl_3$

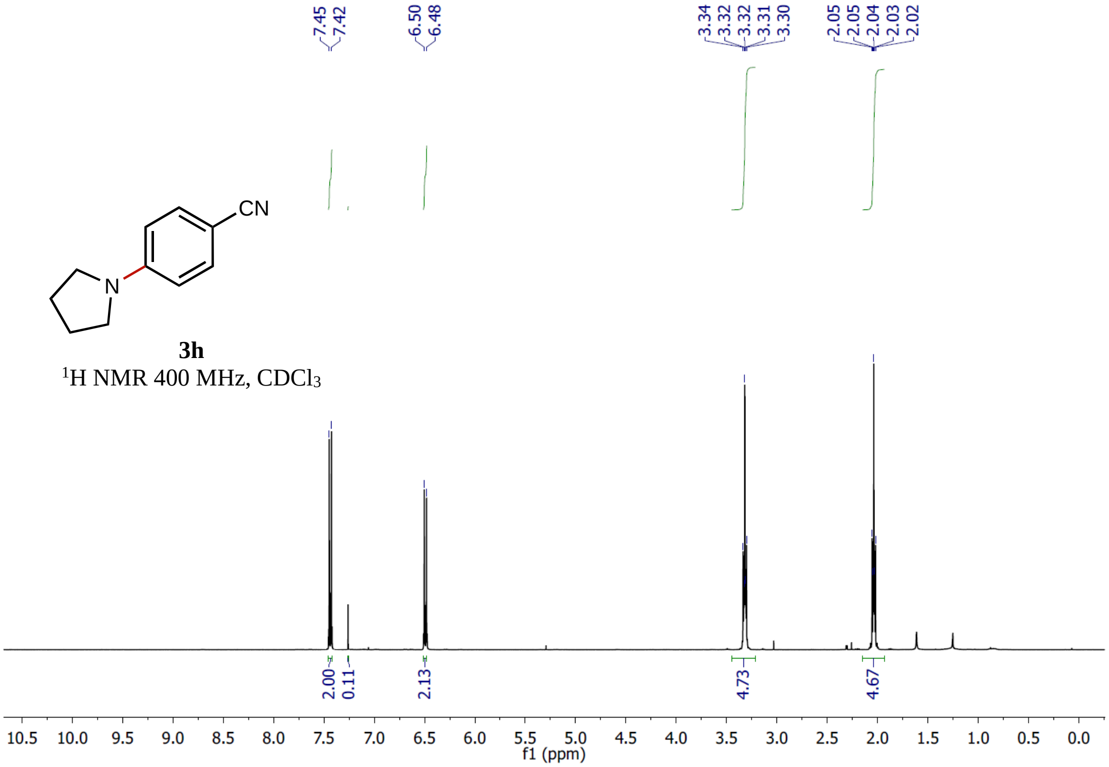

3h

^{1}H NMR 400 MHz, $CDCl_3$

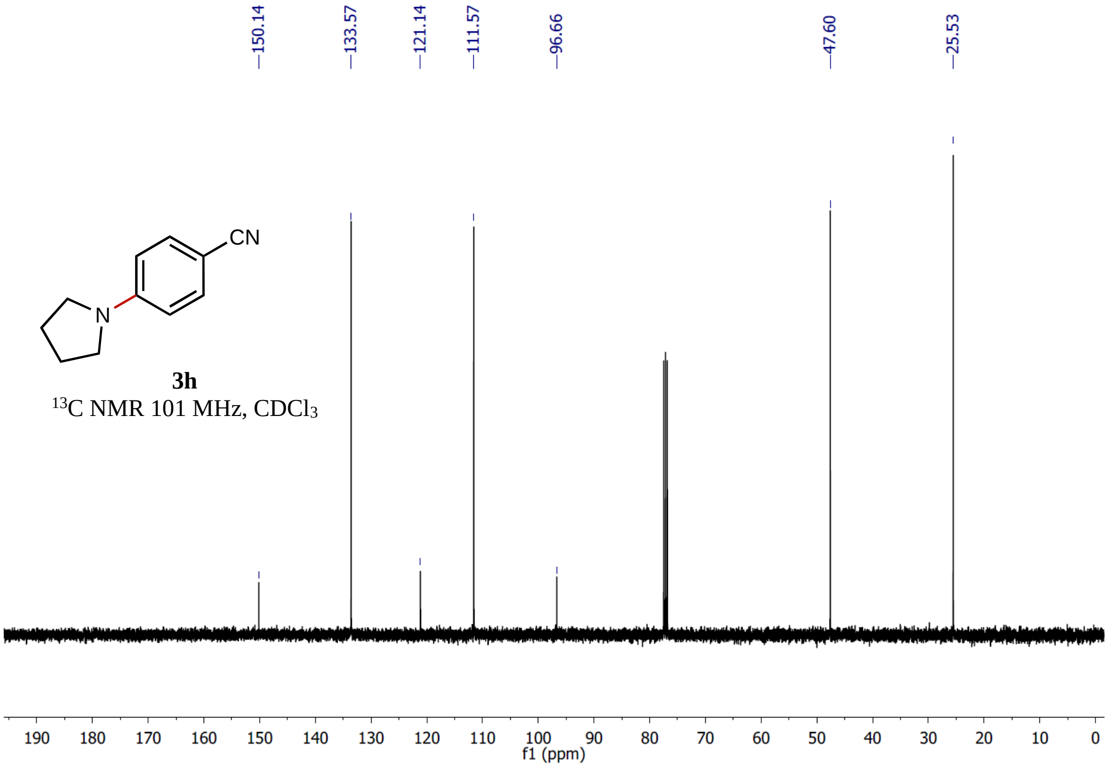

3h

^{13}C NMR 101 MHz, $CDCl_3$

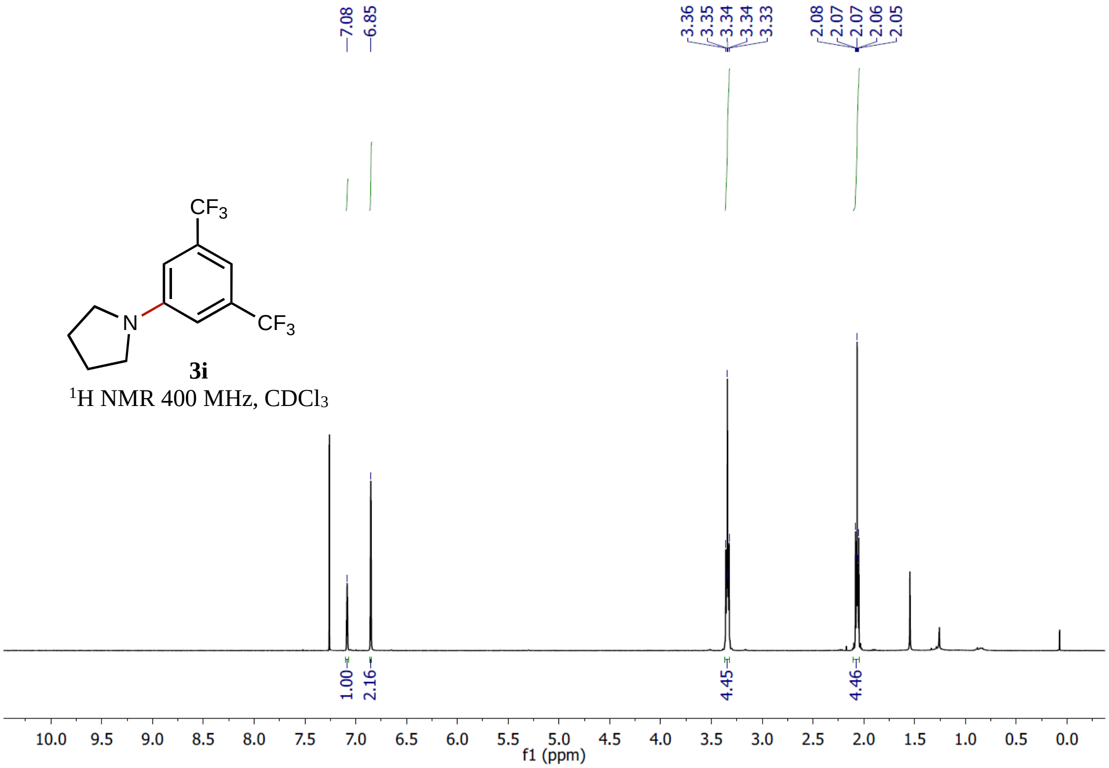
7.08
6.85
3.36
3.35
3.34
3.34
3.33
2.08
2.07
2.07
2.06
2.05
CF3
CF3
N
3i
1H NMR 400 MHz, CDCl3
1.00
2.16
4.45
4.46
10.0
9.5
9.0
8.5
8.0
7.5
7.0
6.5
6.0
5.5
5.0
4.5
4.0
3.5
3.0
2.5
2.0
1.5
1.0
0.5
0.0
f1 (ppm)

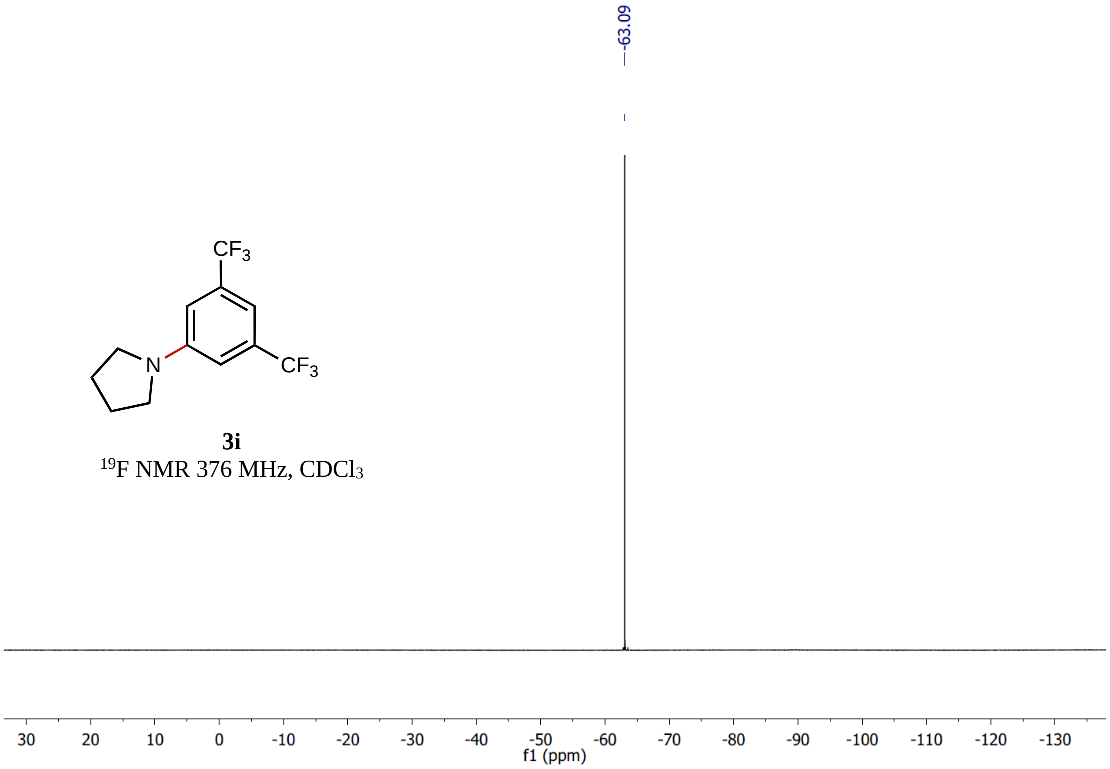
-63.09
CF3
CF3
N
3i
19F NMR 376 MHz, CDCl3
30
20
10
0
-10
-20
-30
-40
-50
-60
-70
-80
-90
-100
-110
-120
-130
f1 (ppm)

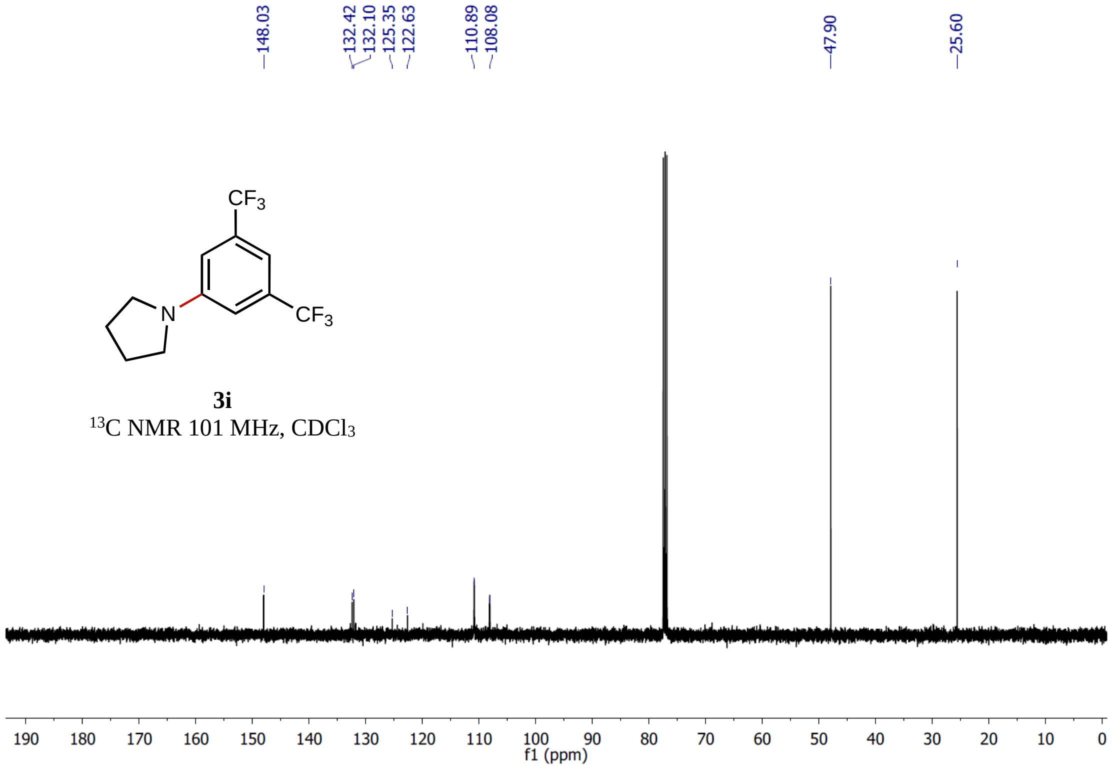

3i

^{13}C NMR 101 MHz, $CDCl_3$

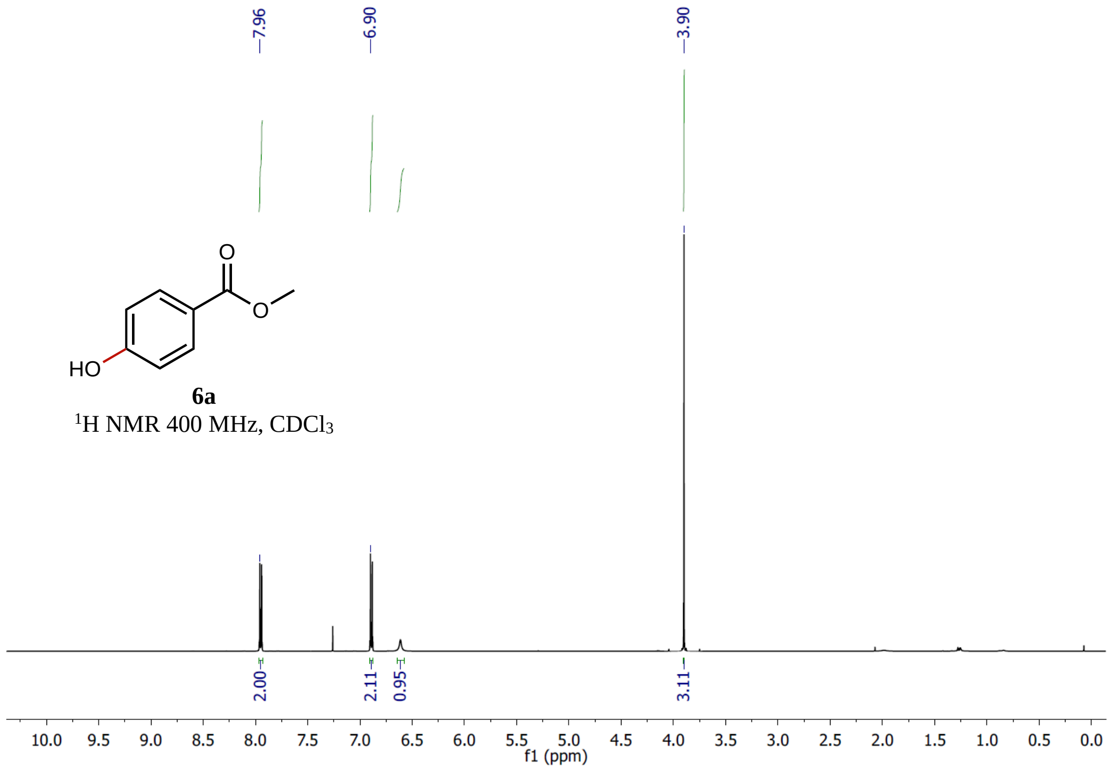

6a

^{1}H NMR 400 MHz, $CDCl_3$

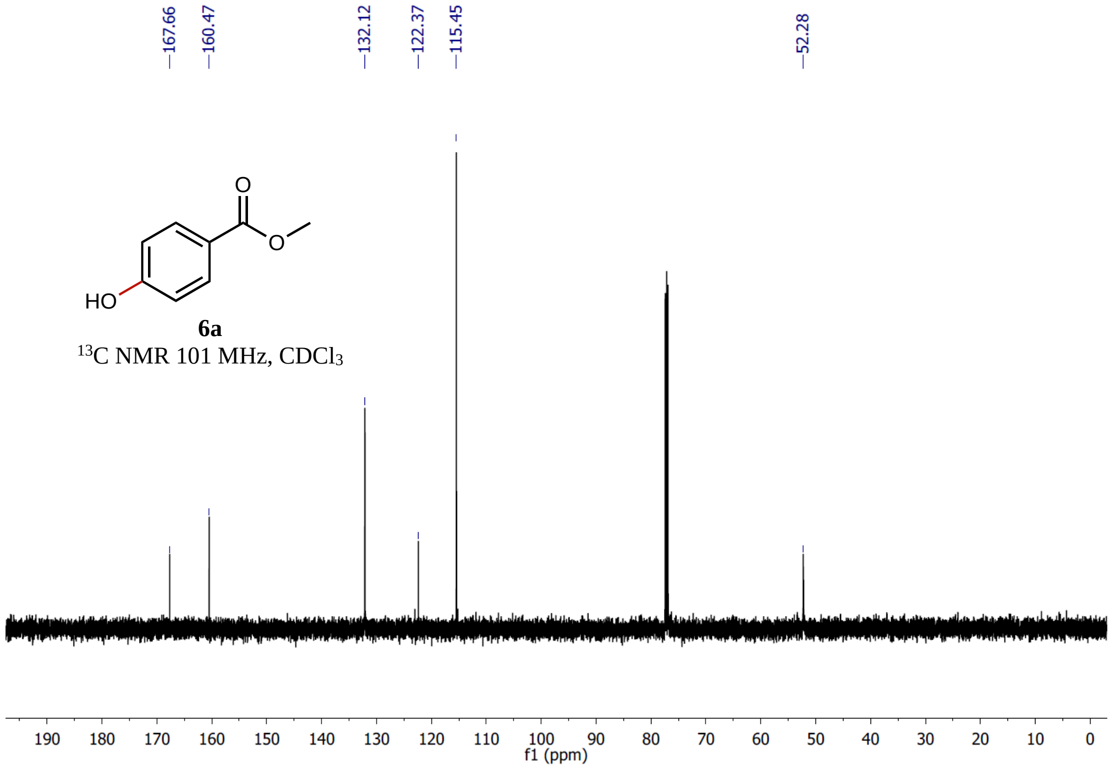

6a

^{13}C NMR 101 MHz, $CDCl_3$

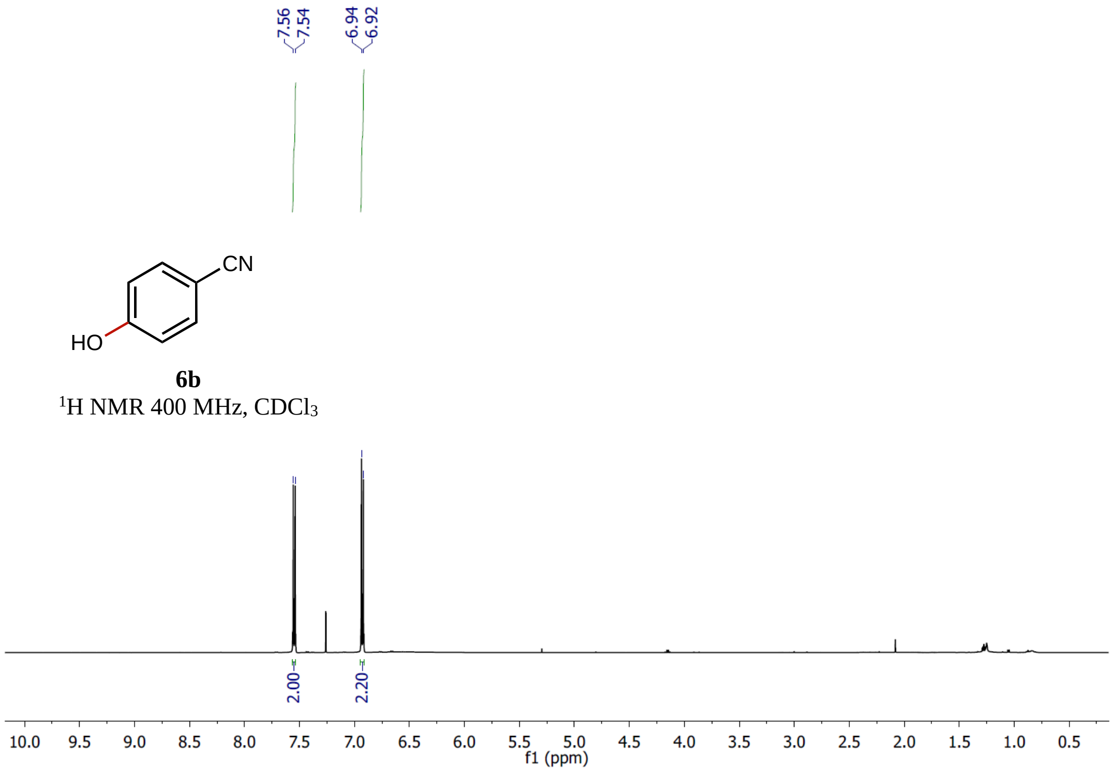

6b

^{1}H NMR 400 MHz, $CDCl_3$

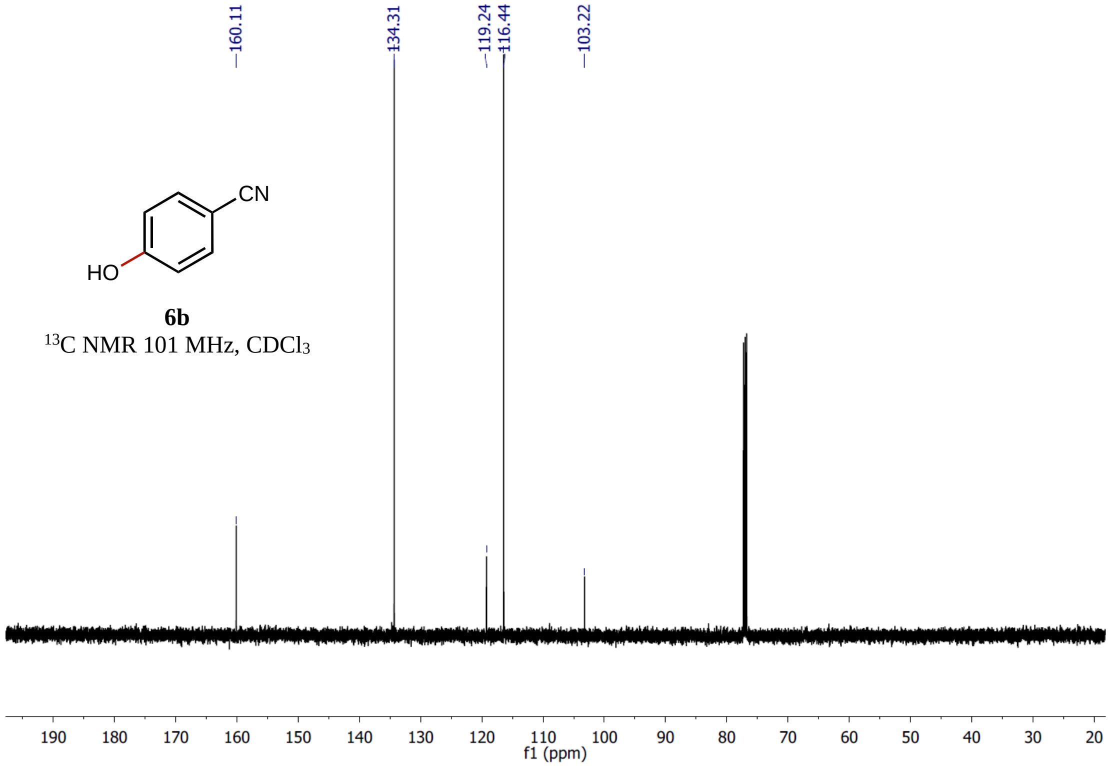

6b

^{13}C NMR 101 MHz, $CDCl_3$

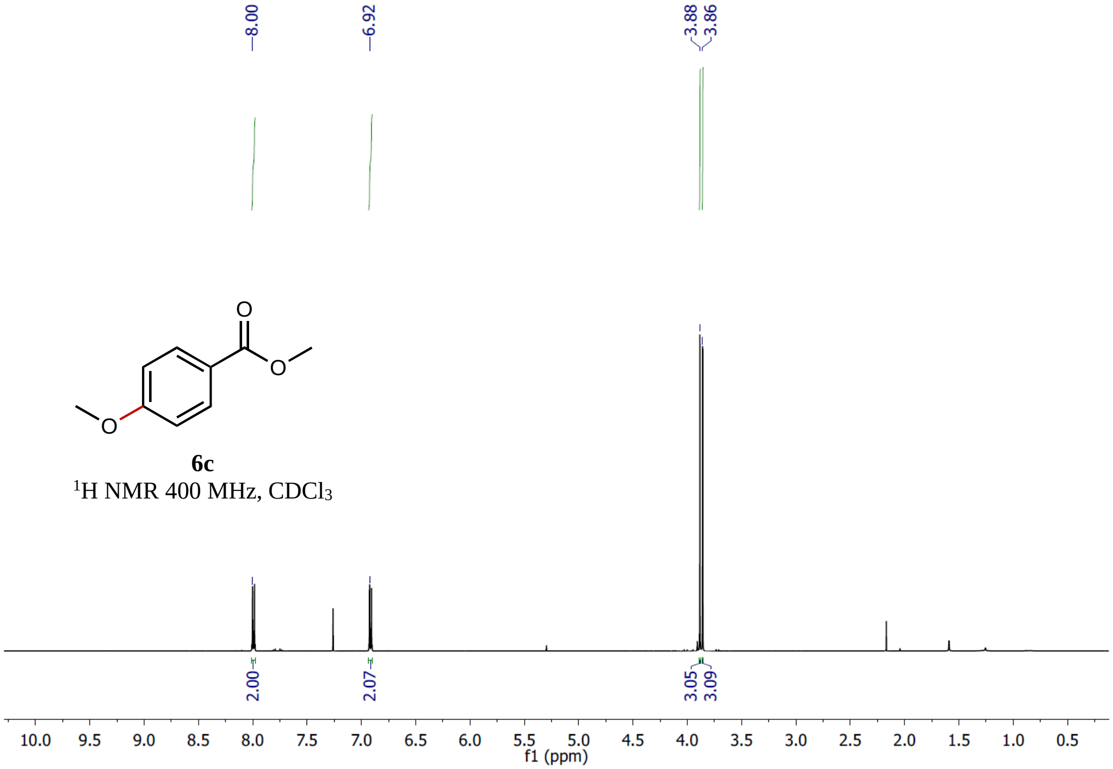

6c

^{1}H NMR 400 MHz, $CDCl_3$

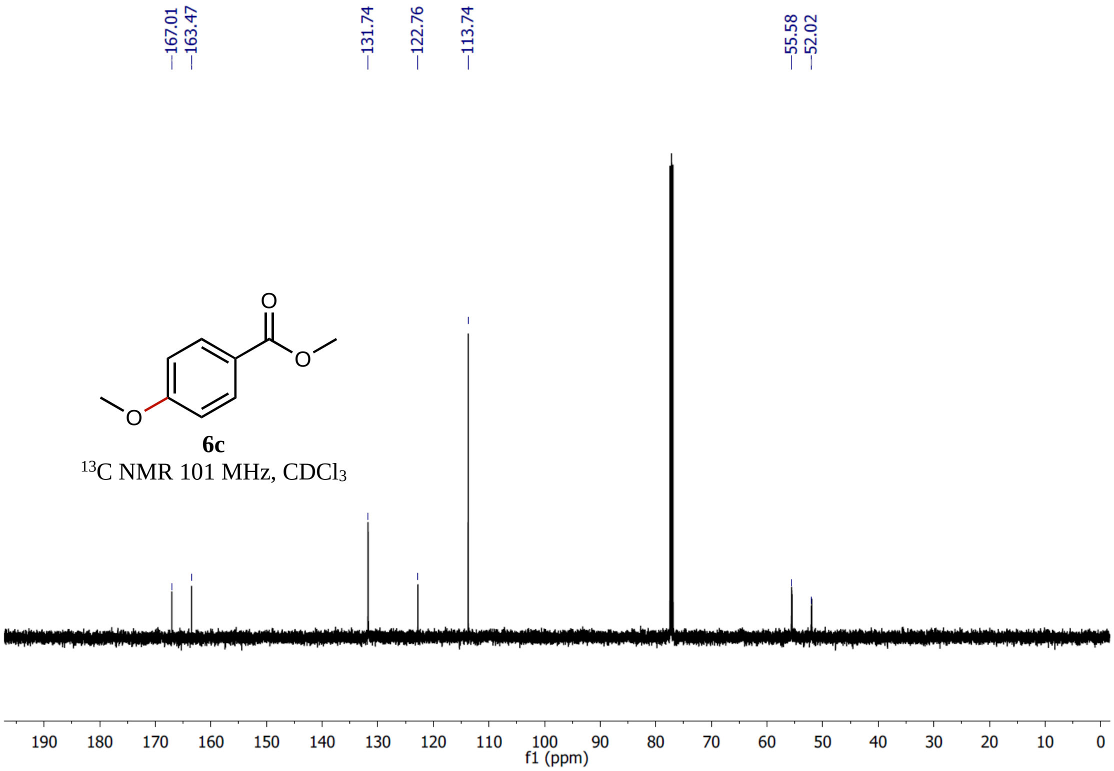
167.01
163.47
131.74
122.76
113.74
55.58
52.02
O
O
O
6c
13C NMR 101 MHz, CDCl3
190 180 170 160 150 140 130 120 110 100 90 80 70 60 50 40 30 20 10 0
f1 (ppm)

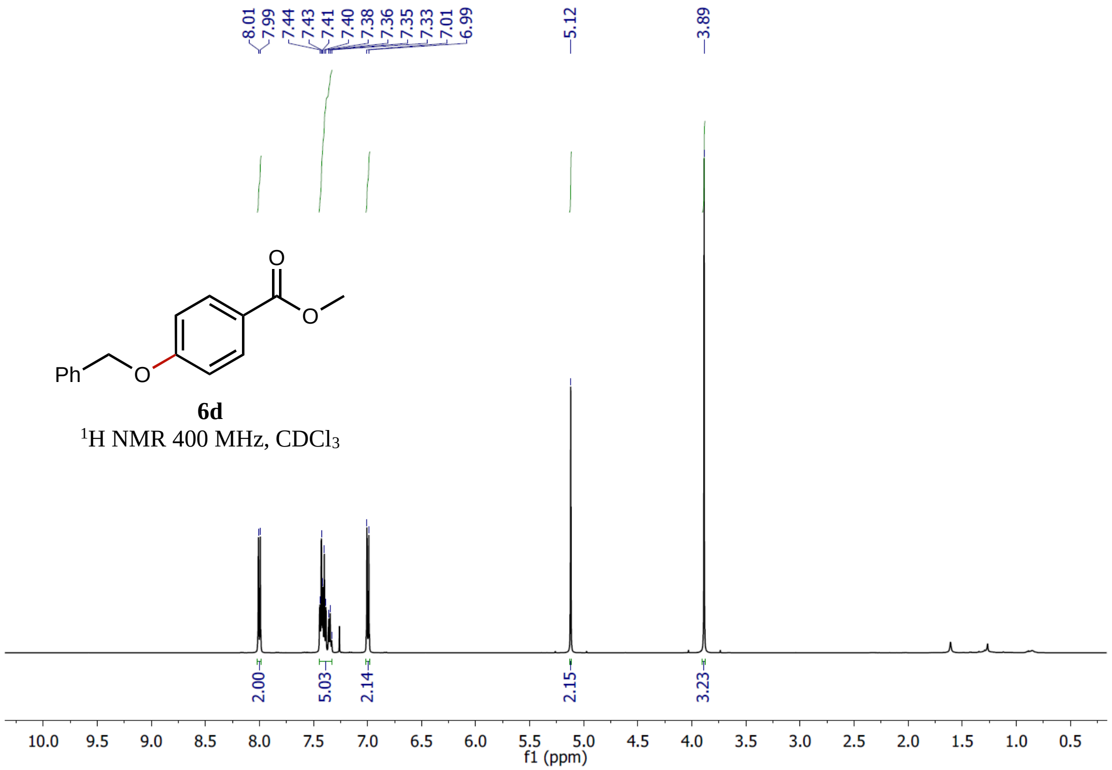
8.01
7.99
7.44
7.43
7.41
7.40
7.38
7.36
7.35
7.33
7.01
6.99
5.12
3.89
O
O
Ph
O
6d
1H NMR 400 MHz, CDCl3
2.00
5.03
2.14
2.15
3.23
10.0 9.5 9.0 8.5 8.0 7.5 7.0 6.5 6.0 5.5 5.0 4.5 4.0 3.5 3.0 2.5 2.0 1.5 1.0 0.5
f1 (ppm)

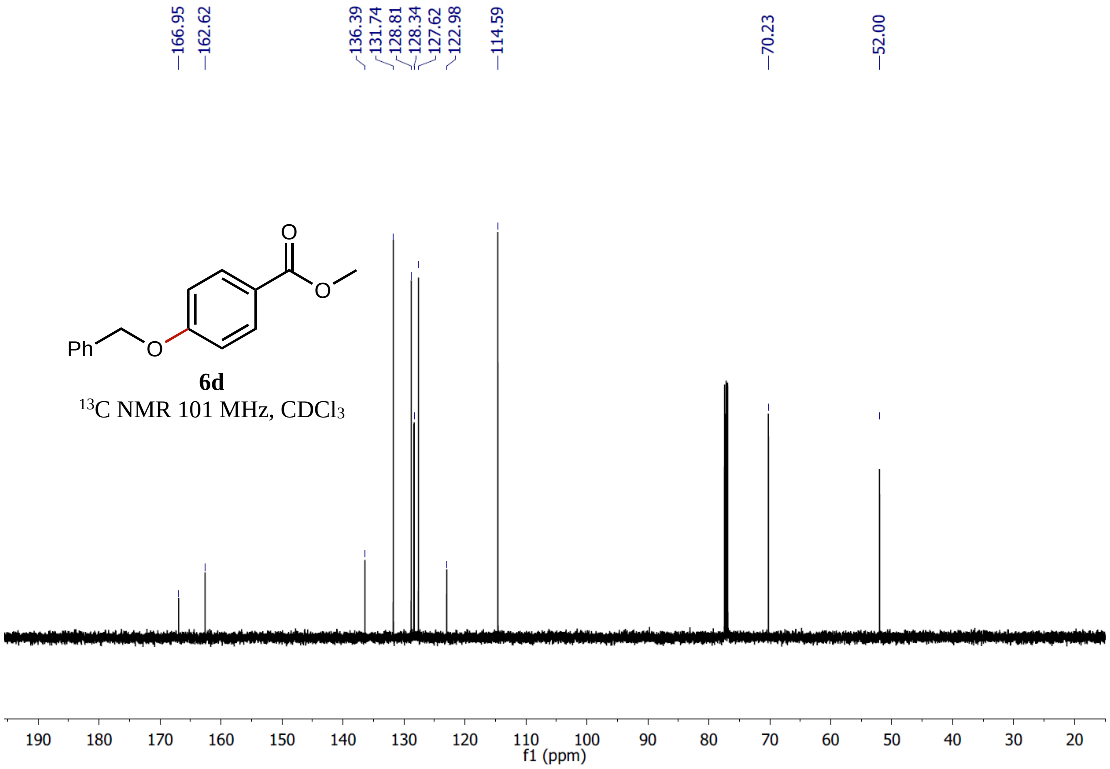

166.95
162.62
136.39
131.74
128.81
128.34
127.62
122.98
114.59
70.23
52.00
Ph
O
O
O
6d
13C NMR 101 MHz, CDCl3
f1 (ppm)

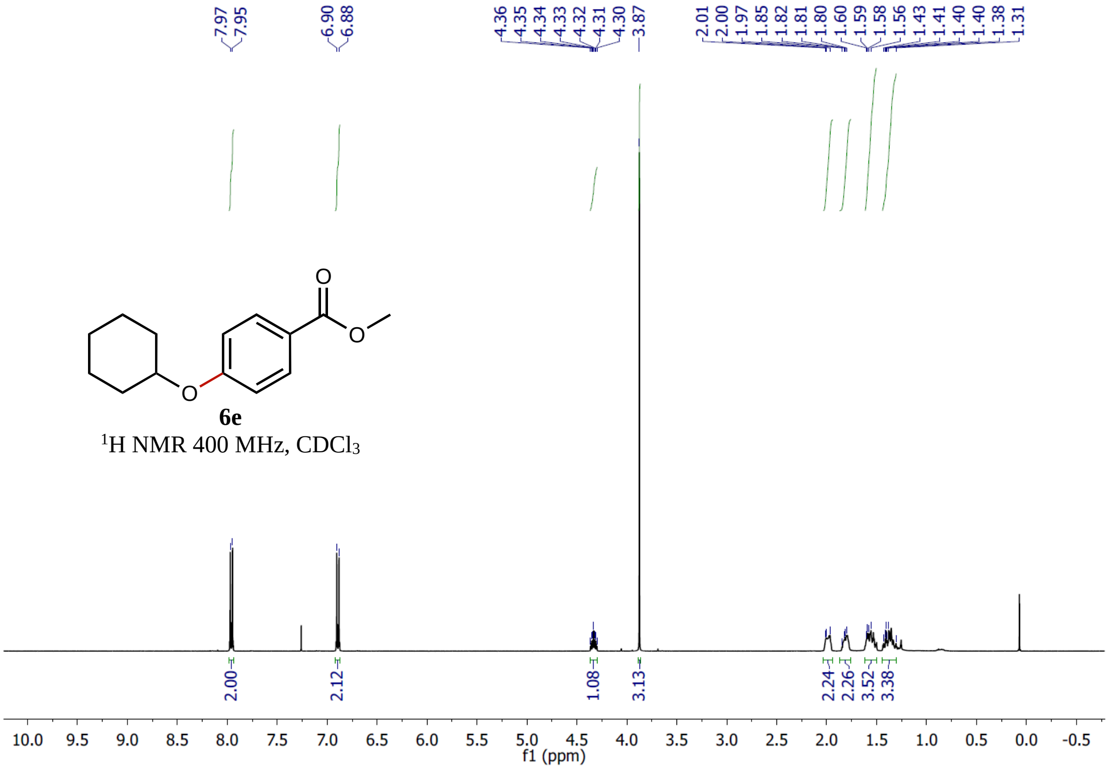

7.97
7.95
6.90
6.88
4.36
4.35
4.34
4.33
4.32
4.31
4.30
3.87
2.01
2.00
1.97
1.85
1.82
1.81
1.80
1.60
1.59
1.58
1.56
1.43
1.41
1.40
1.40
1.38
1.31
O
O
O
6e
1H NMR 400 MHz, CDCl3
2.00
2.12
1.08
3.13
2.24
2.26
3.52
3.38
f1 (ppm)

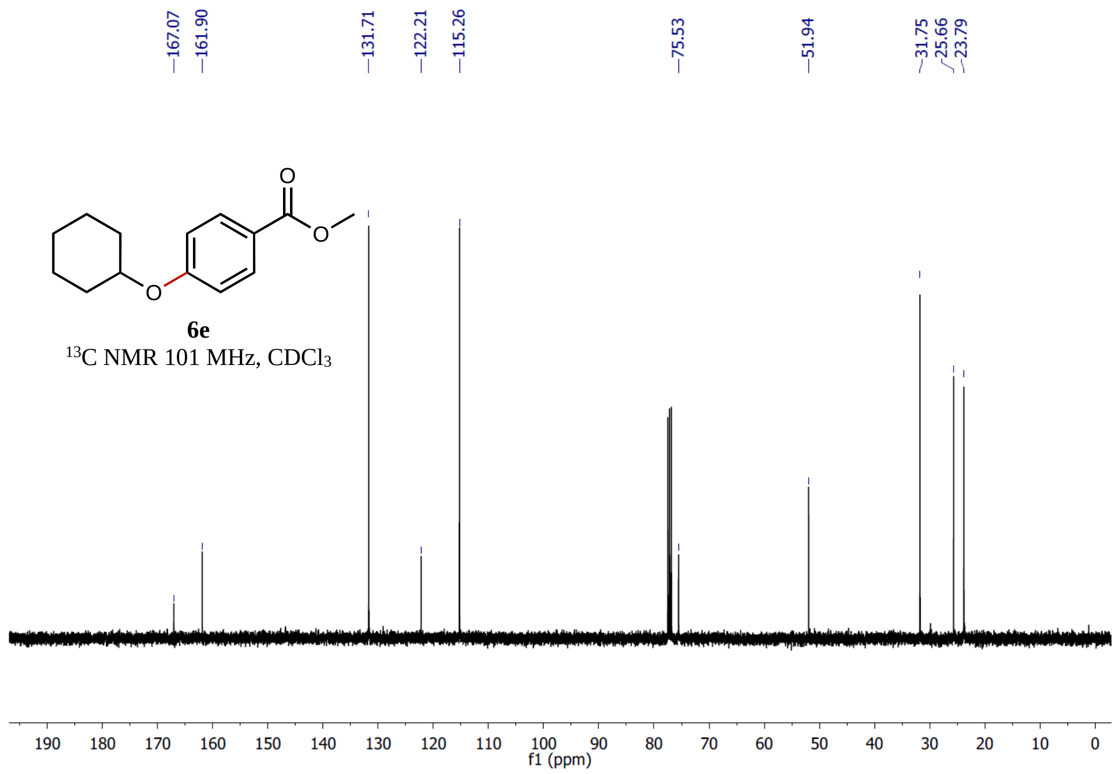

167.07
161.90
131.71
122.21
115.26
75.53
51.94
31.75
25.66
23.79
O
O
O
6e
13C NMR 101 MHz, CDCl3
190 180 170 160 150 140 130 120 110 100 90 80 70 60 50 40 30 20 10 0
f1 (ppm)

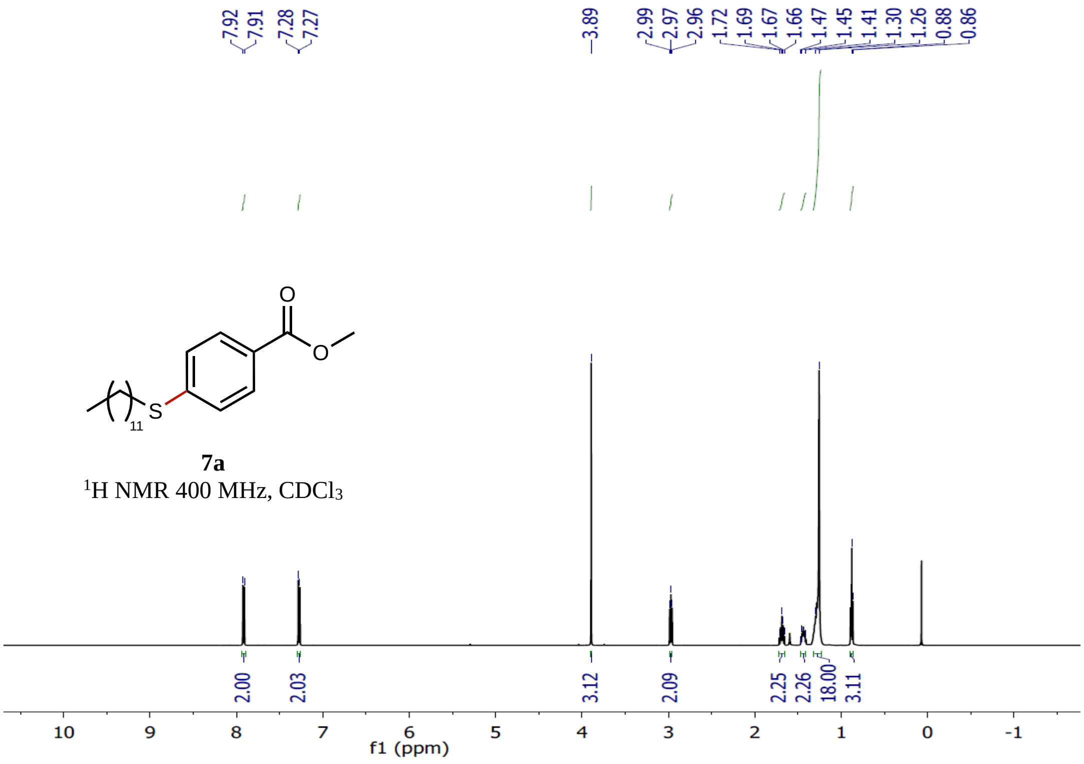

7.92
7.91
7.28
7.27
3.89
2.99
2.97
2.96
1.72
1.69
1.67
1.66
1.47
1.45
1.41
1.30
1.26
0.88
0.86
O
O
S
11
7a
1H NMR 400 MHz, CDCl3
2.00
2.03
3.12
2.09
2.25
2.26
18.00
3.11
10 9 8 7 6 5 4 3 2 1 0 -1
f1 (ppm)

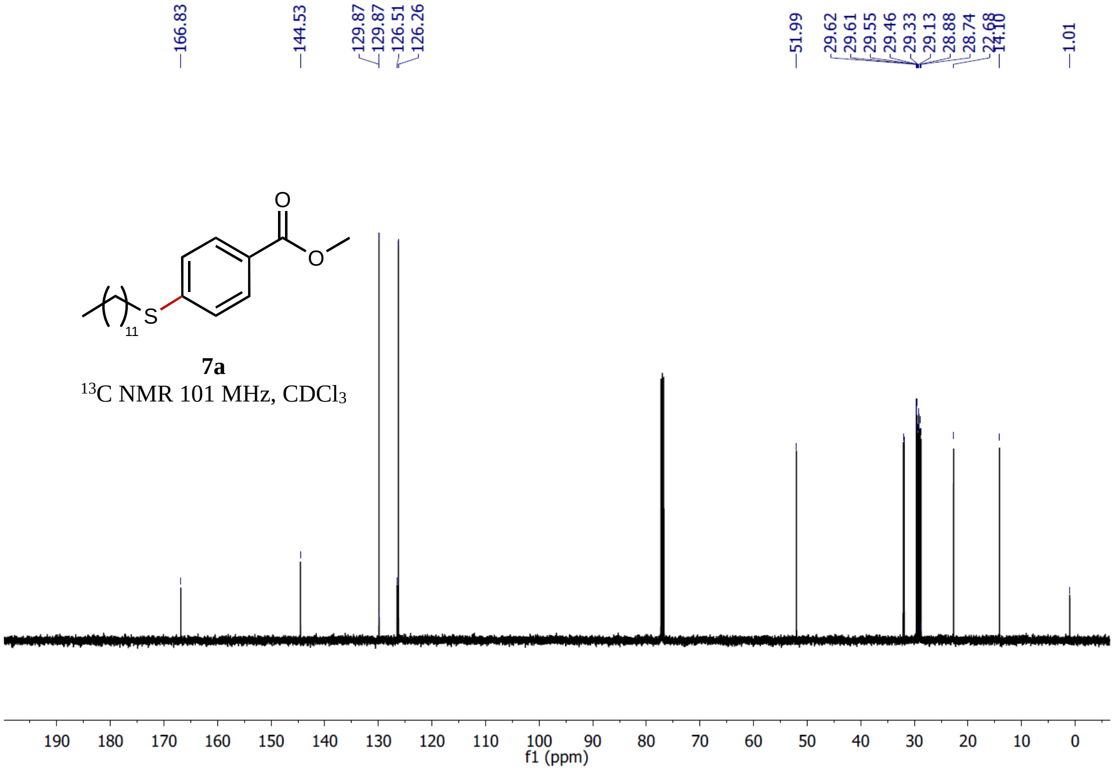

7a

^{13}C NMR 101 MHz, $CDCl_3$

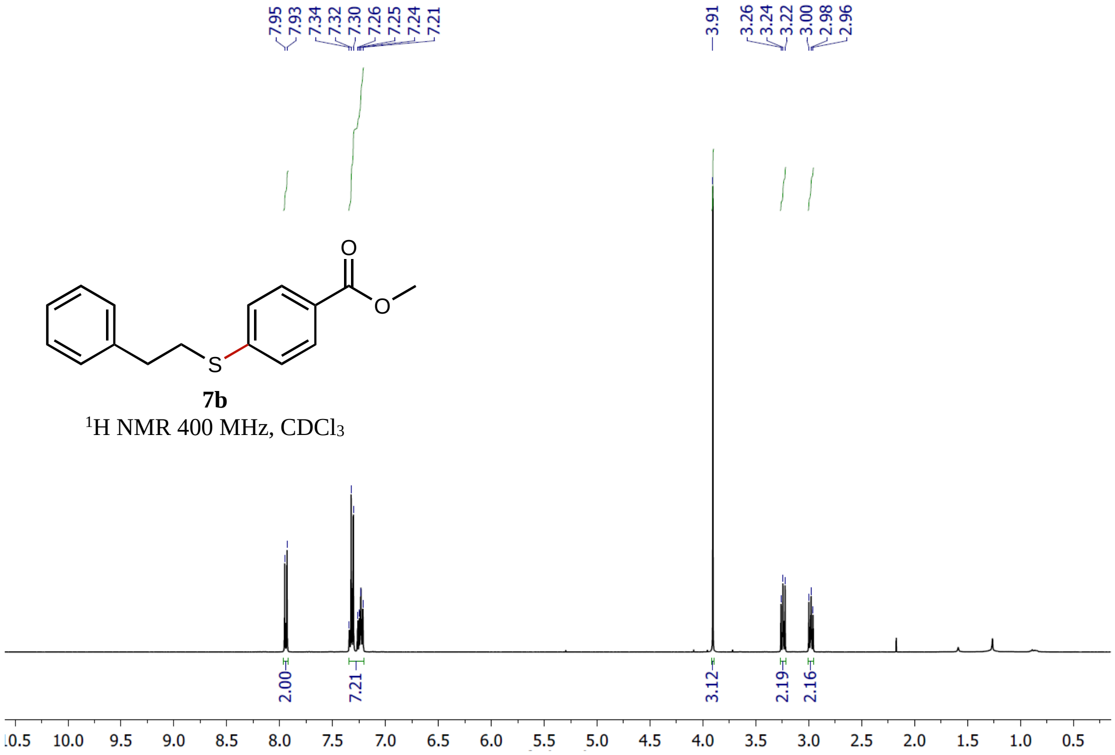

7b

^{1}H NMR 400 MHz, $CDCl_3$

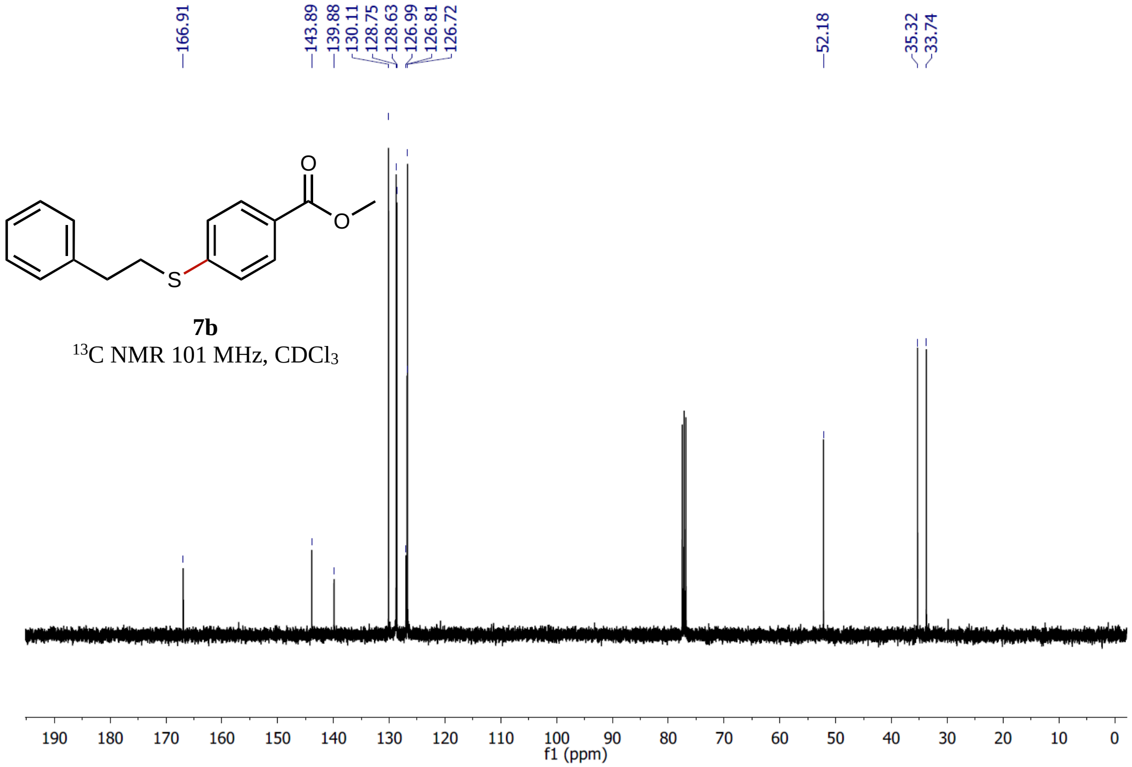

7b
^{13}C NMR 101 MHz, $CDCl_3$

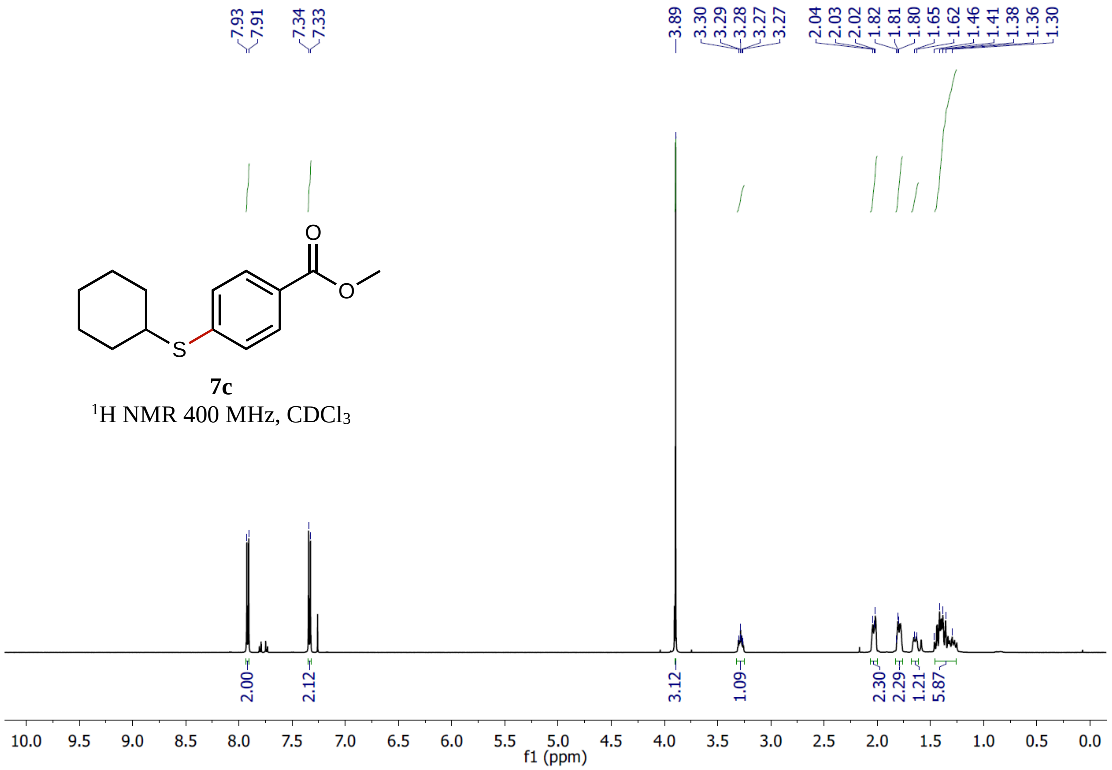

7c
^{1}H NMR 400 MHz, $CDCl_3$

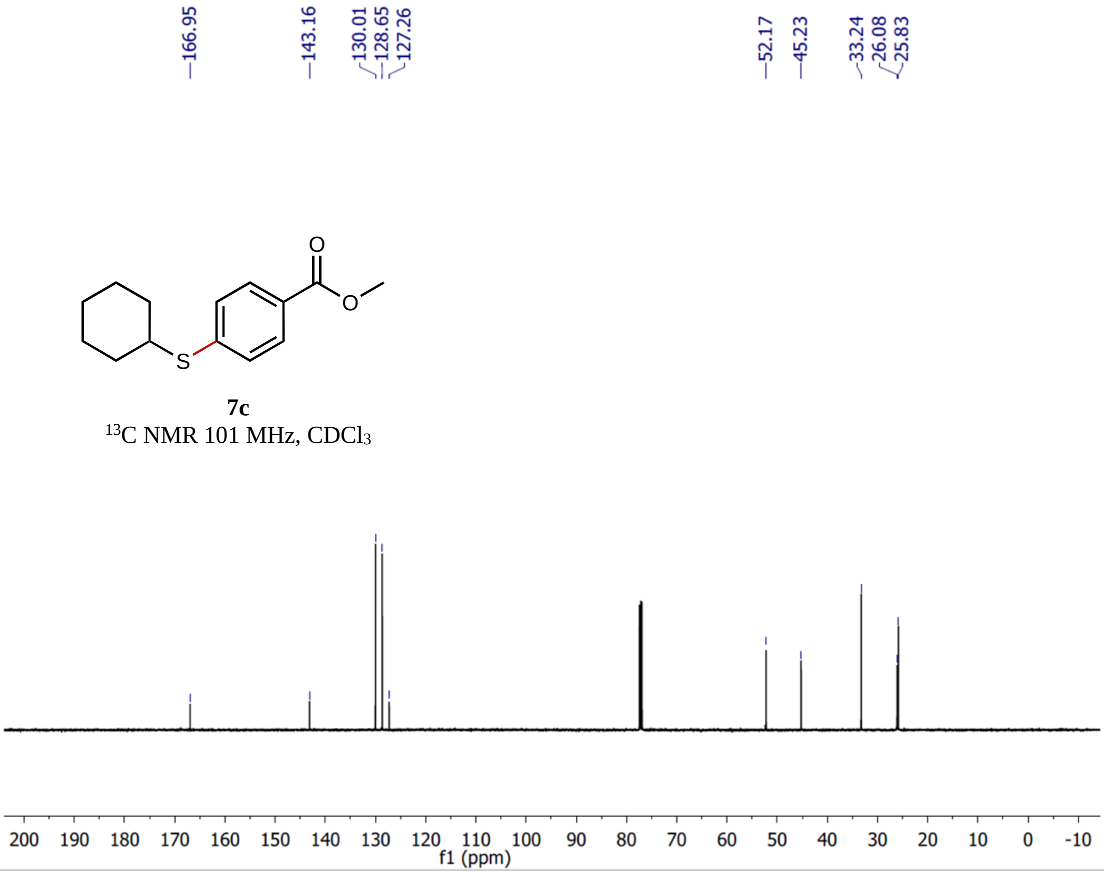
166.95
143.16
130.01
128.65
127.26
52.17
45.23
33.24
26.08
25.83
O
O
S
7c
^{13}C NMR 101 MHz, CDCl$_3$
200 190 180 170 160 150 140 130 120 110 100 90 80 70 60 50 40 30 20 10 0 -10
f1 (ppm)

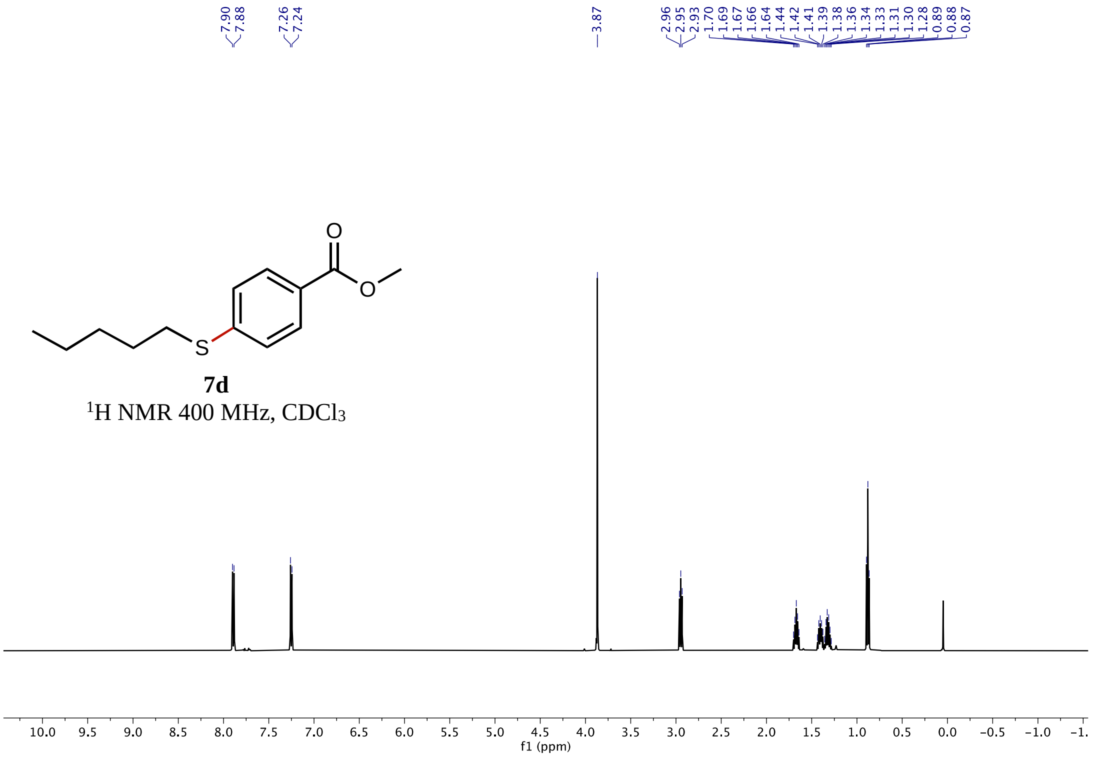
7.90
7.88
7.26
7.24
3.87
2.96
2.95
2.93
1.70
1.69
1.67
1.66
1.64
1.44
1.42
1.41
1.39
1.38
1.36
1.34
1.33
1.31
1.30
1.28
0.89
0.88
0.87
O
O
S
7d
^{1}H NMR 400 MHz, CDCl$_3$
10.0 9.5 9.0 8.5 8.0 7.5 7.0 6.5 6.0 5.5 5.0 4.5 4.0 3.5 3.0 2.5 2.0 1.5 1.0 0.5 0.0 −0.5 −1.0 −1.
f1 (ppm)

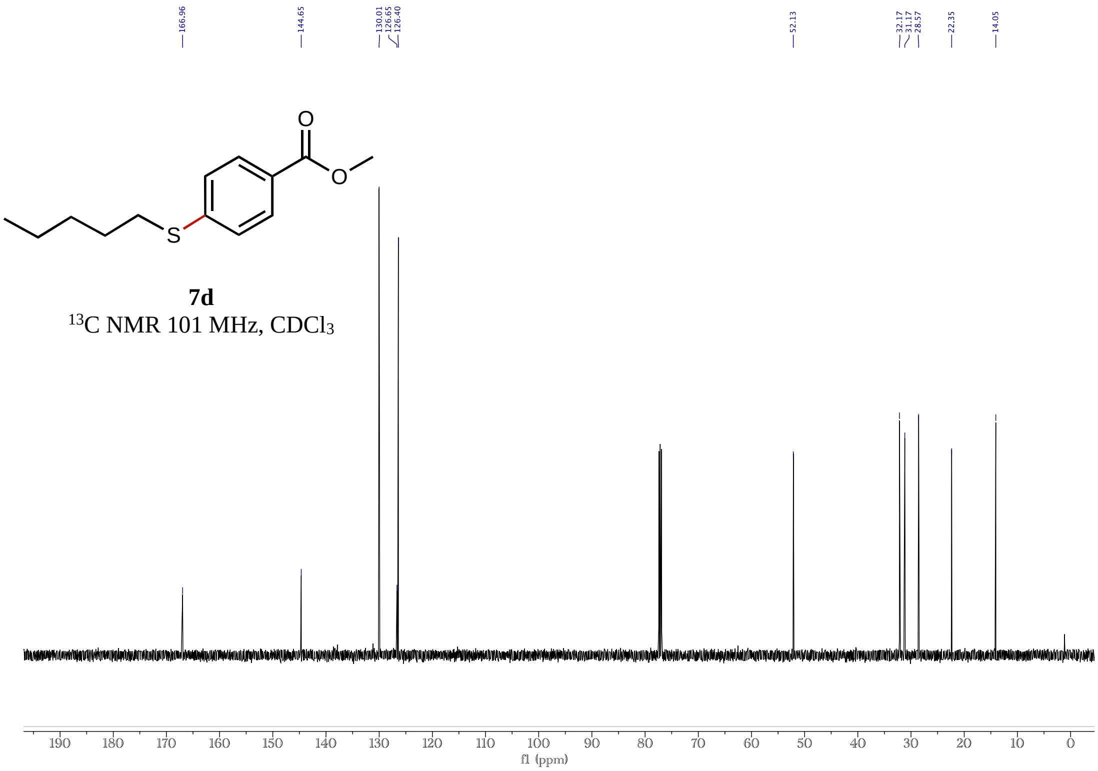

Scanning transmission electron microscopy and electron energy loss spectroscopy

Representative HAADF-STEM images of *g*-CN, *mw*-CN and Ni-complexes, as shown in Figure S3 (a-d)), exhibit random sheet-like interconnected porous structures. *g*-CN and *mw*-CN have retained the structural morphologies after Ni complexation in the dark. The crystallinity and electronic state of CN samples were investigated using STEM-EELS. The core loss EELS spectra show the absence of any substantial changes in the position and peak shape of C and N. Near edge features of C and N edges show similar features (Figure S3 (e)), indicating the same level of crystallinity in all catalysts. The signal intensity maps of C and N edges reveal a homogeneous distribution of C and N across the catalysts (Figure S3 (f-i)). However, quantifying EELS spectra acquired stochastically from various samples reveal a more uniform ratio of C/N in mw-CN compared to *g*-CN. No substantial Ni signals were detected through EDS due to the small atomic concentration of Ni in Ni-complexed CN catalysts. The structural stability of the catalysts was also investigated and shown in Figure S4. The catalysts have retained the porous morphology (Figure S4 (a,b) and crystallinity (EELS spectra shown in Figure S4(c). The quantification of

EELS spectra shows similar behaviour towards C/N ratio, indicating the better structural stability of mw-CN during catalysis. However, EDS spectrum maps of the recovered catalysts show the aggregation of Ni over the period and subsequent oxidation of Ni to form NiO_x particles (Figure S4 (d and e)).

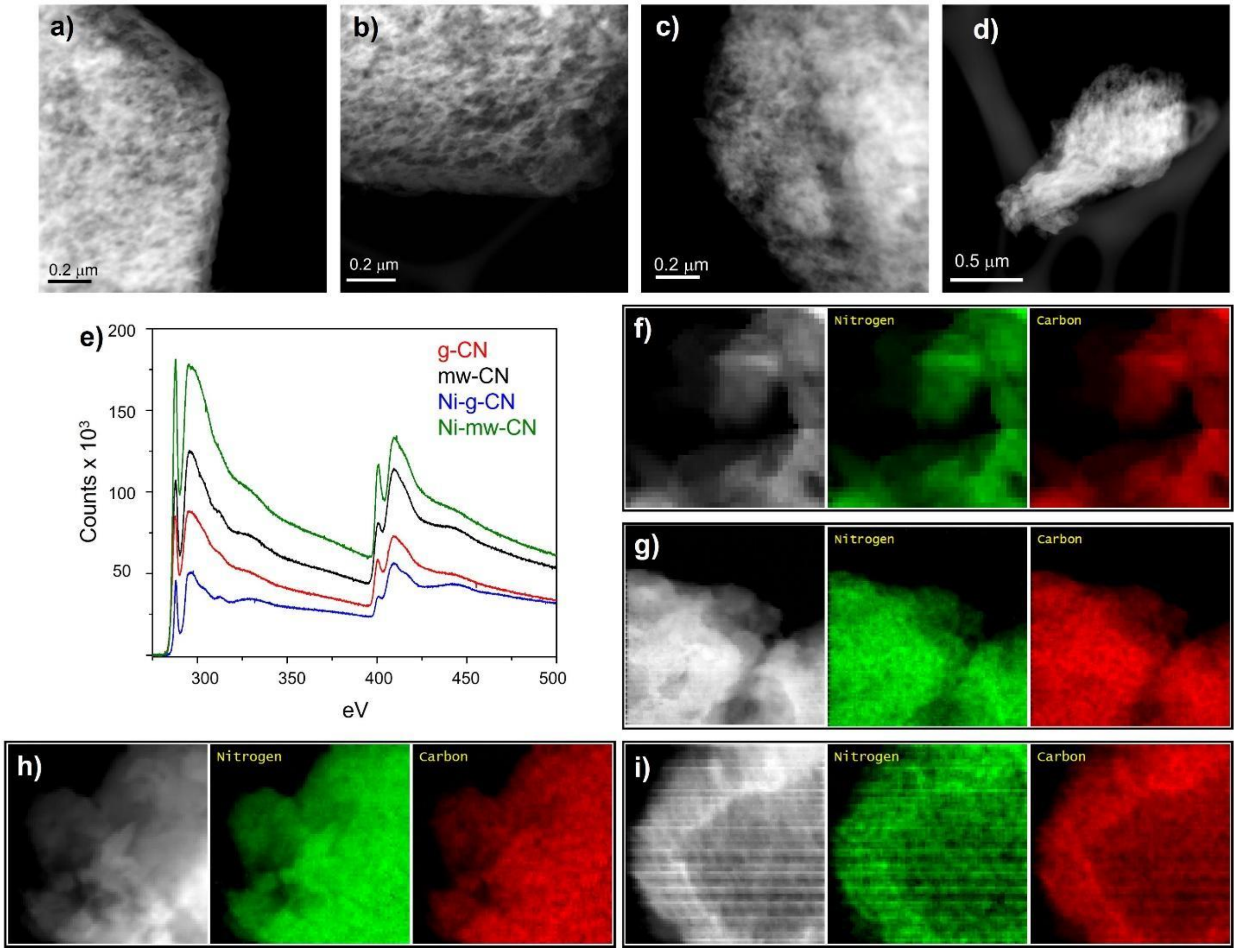

Figure S3. Representative low-magnification high angle annular dark field scanning transmission electron microscopy (HAADF-STEM) images showing the morphology of (a) *g*-CN, (b) *mw*-CN, (c) Ni-*g*-CN (d) Ni-*mw*-CN respectively, and (e) Exponential background fitted EELS spectra of *g*-CN, *mw*-CN, Ni-*g*-CN and Ni-*mw*-CN showing C K and N K edge, (f, g, h, and i) EELS map from C K and N K edges of *g*-CN, mw-CN, Ni-*g*-CN and Ni-*mw*-CN, respectively, showing the uniform distribution of C and N.

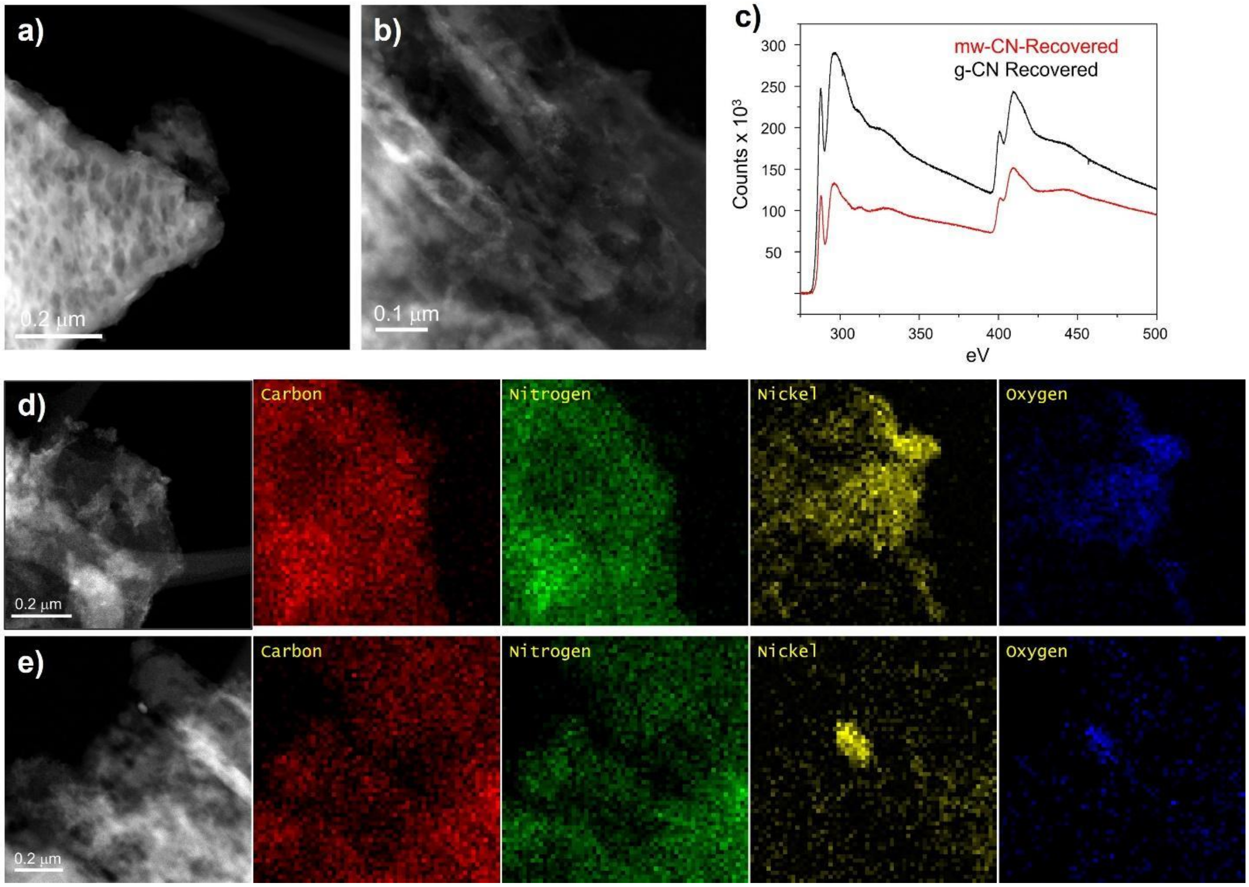

Figure S4. Representative low-magnification HAADF-STEM image of (a) *g*-CN recovered (b) *mw*-CN recovered; (c) EELS spectra of *g*-CN recovered, and *mw*-CN recovered showing C K and N K edge, (d and e) HAADF-STEM image and EDS map from an ROI of the image showing the EDS signal map of C, N, Ni and O, of *g*-CN recovered and *mw*-CN recovered, respectively.

Solution NMR sample preparation

To prepare solid samples for the solution NMR experiments, the photocatalyst solid particles were soaked in the liquid under investigation (dimethylformamide, DMF or *n*-octane) for 48 hours to ensure full saturation of the solid. The saturated solid samples were then transferred to 5 mm NMR tubes. To minimize errors due to evaporation of the liquid, a small amount of pure liquid was dropped onto a filter paper, which was placed under the cap of the NMR tube. The NMR tube was then placed into the magnet and left for approximately 10 min to achieve thermal equilibrium before measurements started.

Low-field NMR relaxation measurements

Low-field NMR relaxation and diffusion experiments were performed using a ^{1}H Magritek SpinSolve 43-MHz benchtop NMR spectrometer. NMR diffusion experiments were carried out using a diffusion probe capable of producing magnetic field gradient pulses up to 163 mT m^{-1}. All low-field NMR measurements were performed at atmospheric pressure and 25 °C.

T_1 was measured using the inversion recovery pulse sequence, (Eq. S1) with a repetition time of 5 × T_1, acquiring 16-time delay steps logarithmically spaced with 4 scans per step. T_1 is determined by plotting the acquired signal intensities as a function of the time delay, τ in accordance with the following equation:

$$\frac{M_z(t)}{M_O} = 1 - 2\,e^{-\frac{\tau}{T_1}} \qquad (Eq.S1)$$

Where M_z *(t)* is the signal intensity at the respective time delay, τ, M_O is the signal intensity at equilibrium and τ is the time delay.

T_2 was measured using the CPMG pulse sequence (Figure S5) using an echo time of 1 ms with 16 steps, using two echoes per step and 4 scans per step. The typical error on all T_1 and T_2 measurements was less than 5 %. The spin-lattice relaxation time constant is determined by plotting the acquired signal intensities as a function of the time delay, τ in accordance with the following equation:

$$M_{xy} = M_{xy}(0) \cdot \exp\left(-\frac{t}{T_2}\right) \qquad (Eq.S2)$$

Schematic representations of the pulse sequences used can be seen in Figure S5.

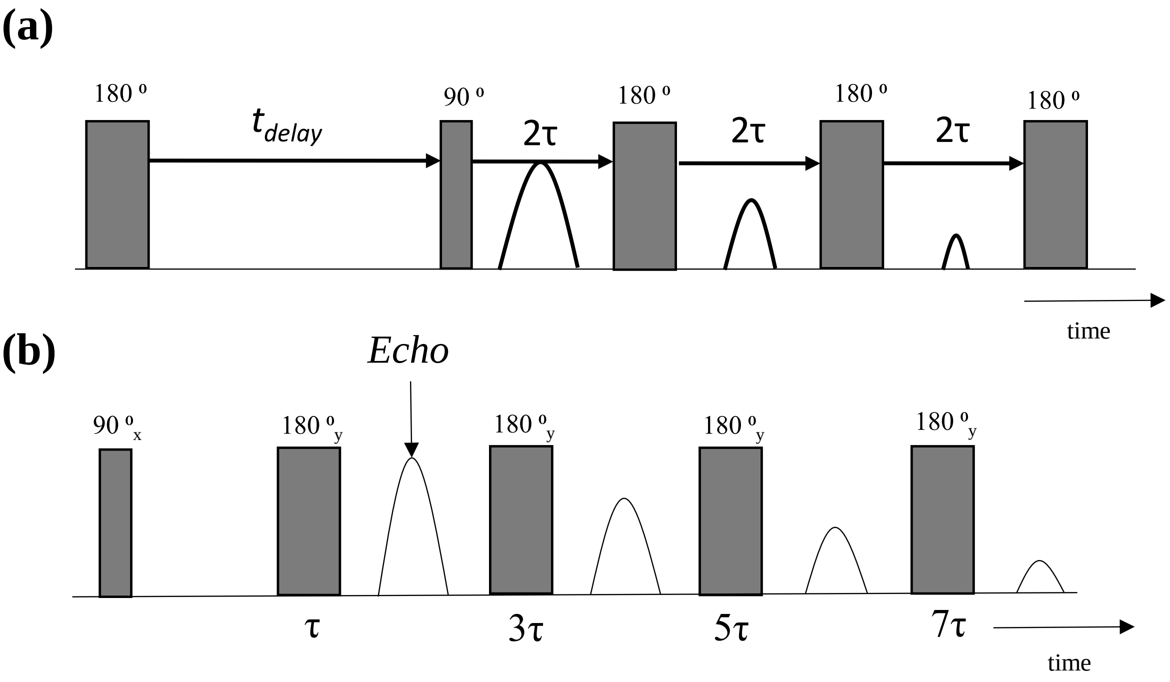

Figure S5. (a) The inversion recovery pulse sequence and (b) the CPMG echo pulse sequence used to obtain the NMR relaxation times T_1 and T_2 respectively.

For all measurements, a pulse length of 18 μs was used, with a pulse amplitude of −6 dB for the 90° pulse and 0 dB for the 180° pulse, a receiver gain of 40, and acquiring 16834 points in the time domain with a dwell time of 20 μs.

The ranges of parameters varied for the inversion recovery and CPMG experiments (maximum inversion delays and echoes per step respectively) are listed in **Error! Reference source not found.**

Table S3. Parameter ranges used in inversion recovery and CPMG experiments for the measurement of the NMR relaxation times T_1 and T_2 of substrates imbibed within the pores of the photocatalysts studied.

**Substrate**	**Max inversion delay range (ms)**	**Number of echoes per step**
DMF	5000 – 15000	120 – 1500
n-Octane	10000	3300 – 3600

The plots and relaxation time values determined from ^{1}H NMR relaxation measurements of DMF and *n*-octane imbibed within the pores of the photocatalyst samples can be seen in Figure S6 –S7 and Table S4 –S5.

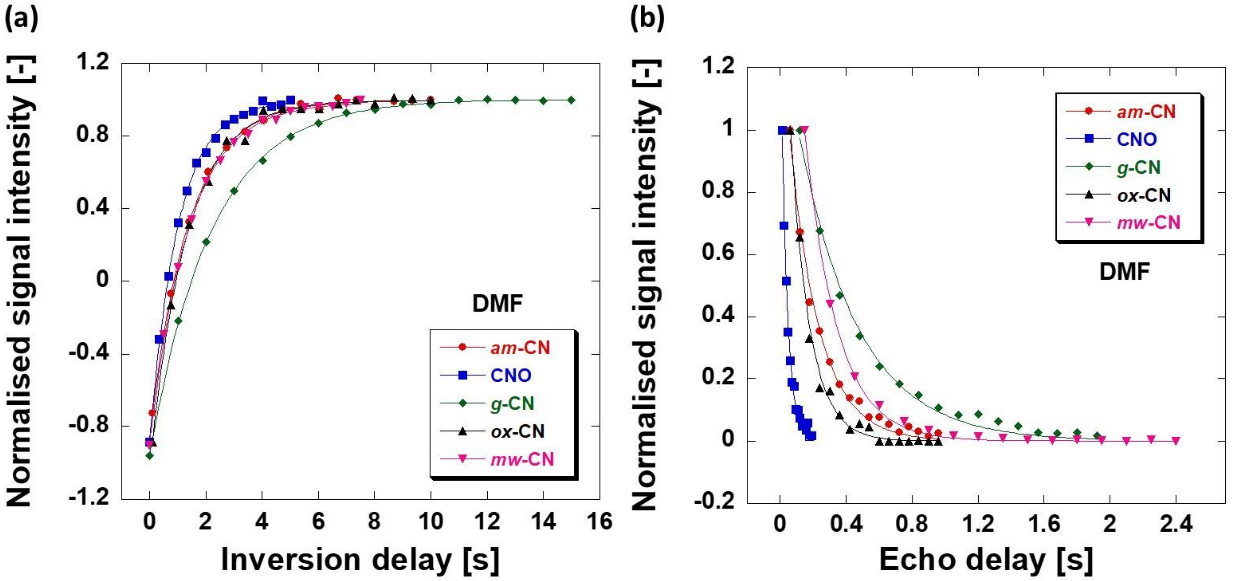

Figure S6. (a) T_1 inversion recovery and (b) T_2 CPMG echo plots obtained using DMF confined within the pores of the various photocatalysts.

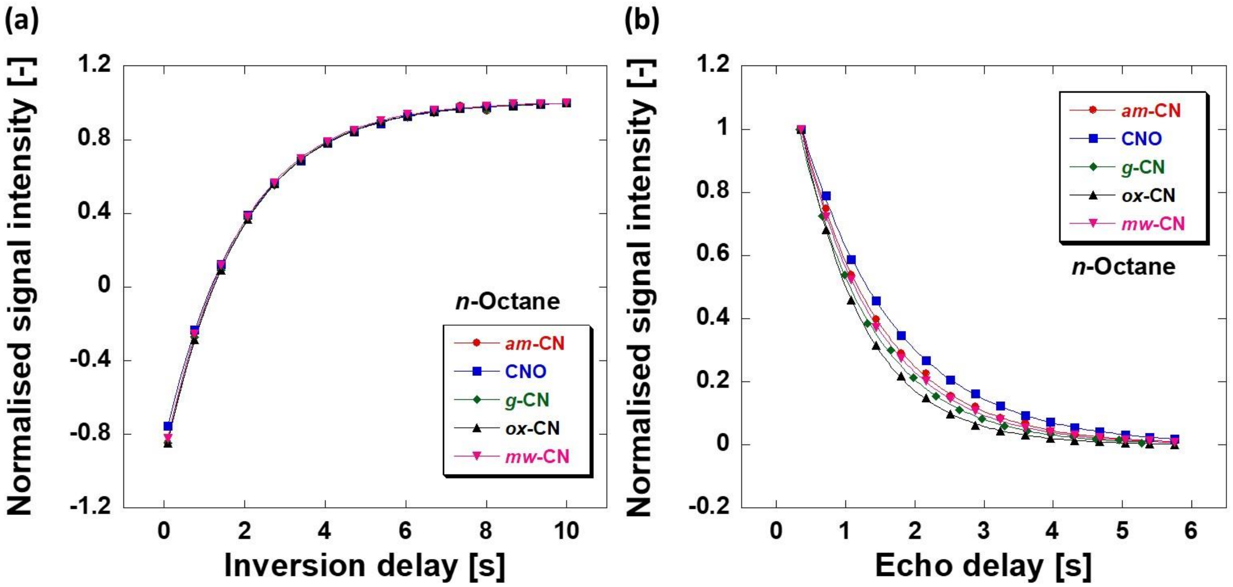

Figure S7. (a) T_1 inversion recovery and (b) T_2 CPMG echo plots obtained using *n*-octane confined within the pores of the various photocatalysts.

Table S4. T_1, T_2 and calculated T_1/T_2 values of DMF imbibed within the pores of the various photocatalysts obtained using low-field NMR relaxation measurements. The error of all T_1 and T_2 values is 3 % and the error of all T_1/T_2 values is 5%.

Dopant	T_1 (ms)	T_2 (ms)	T_1/T_2 (-)
am-CN	1429 ± 43	175 ± 5	8.1 ± 0.4
CNO	1010 ± 30	37 ± 1	27.6 ± 1.4
g-CN	2222 ± 67	357 ± 11	6.2 ± 0.3
ox-CN	1333 ± 40	119 ± 4	11.2 ± 0.6
mw-CN	1389 ± 42	192 ± 6	7.2 ± 0.4

Table S5. T_1, T_2 and calculated T_1/T_2 values of *n*-octane imbibed within the pores of the various photocatalysts obtained using low-field NMR relaxation measurements. The error of all T_1 and T_2 values is 3 % and the error of all T_1/T_2 values is 5%.

**Dopant**	T_1 **(ms)**	T_2 **(ms)**	T_1/T_2 **(-)**
am-CN	1855 ± 56	1176 ± 35	1.6 ± 0.1
CNO	1913 ± 57	1363 ± 41	1.4 ± 0.1
g-CN	1855 ± 56	1064 ± 32	1.7 ± 0.1
ox-CN	1864 ± 56	935 ± 28	2.0 ± 0.1
mw-CN	1849 ± 55	1119 ± 34	1.7 ± 0.1

In brief, if a fluid is confined in a porous medium, the measured relaxation times (T_1, T_2) are given by:[1]

$$\frac{1}{T_{1,\text{pore}}} = \frac{1}{T_{1,\text{bulk}}} + \rho_1 \frac{S}{V} \qquad (Eq. S3)$$

$$\frac{1}{T_{2,\text{pore}}} = \frac{1}{T_{2,\text{bulk}}} + \rho_2 \frac{S}{V} \qquad (Eq. S4)$$

The first terms of these equations represent the relaxation time, T_x, of the bulk solvent; in the second term, attributed to water close to or in contact with the surface, ρ_x is the surface relaxivity, which depends on the intrinsic surface chemistry of the material and is effectively a measure of how strongly the guest molecule interacts with the surface and $\frac{S}{V}$ is the surface-to-volume ratio. When T_2 values are considered, there is also an additional term dependent upon the diffusivity of the molecule under background gradients. However, this diffusivity term can be neglected in the case of sufficiently small echo times and low magnetic fields, which is the case in this study.

Generally, for high $\frac{S}{V}$ materials, the bulk liquid term is negligible as the T_x of a bulk liquid is usually much greater than that of the same liquid confined inside pore structure (for example, for bulk liquid water $T_1 \approx 2.9$ s), hence one can write:[2]

$$\frac{1}{T_{x,\text{pore}}} \approx \rho_x \frac{S}{V} \qquad (Eq. S5)$$

A change of T_x in pores relative to bulk can therefore be due to either a variation of adsorbate/adsorbent surface interaction, reflected in ρ_x, or in a change in internal surface area and pore dimension, reflected in $\frac{S}{V}$.

It follows that when T_1 and T_2 are determined for the same guest molecule within the same porous media, then the surface-area-to-volume ratio term is constant and the dependence of the relaxation times upon the pore structures can be removed by taking T_1/T_2. This ratio is therefore a measurement of only the surface interactions occurring between the guest molecule and the pore walls/surface and is defined as:

$$\frac{T_1}{T_2} \approx \frac{\rho_2}{\rho_1} \qquad (Eq. S6)$$

Previous work has shown that this ratio is related to surface adsorption and can be directly correlated to adsorption energies determined using temperature programmed desorption (TPD).[3] As such, T_1/T_2 can find great use in the field of catalysis and surface science to quantify the strength of surface interactions taking place and explain phenomena such as solvent effects.[4]

In the catalysts for which lower reaction yields were obtained, there is a marked difference between the T_1/T_2 values obtained using DMF and *n*-octane. That is, DMF interacts strongly with the catalyst surface (high T_1/T_2) whereas *n*-octane interacts very weakly with the catalyst surface (low T_1/T_2). This suggests that in the lower yield samples, hydrogen bonding between the solvent molecules and hydroxyl groups located on the catalyst surface is significant. Therefore, inhibition of the catalytic reaction may occur due to blockage of catalytically active sites by DMF molecules.

Pulsed field gradient (PFG)-NMR diffusion measurements

PFG NMR experiments were carried out using a diffusion probe capable of producing magnetic field gradient pulses up to 163 mT m^{-1}. Diffusion measurements were performed using the pulsed-field gradient stimulated echo sequence (PGSTE sequence).[5] The sequence consists of a series of radiofrequency pulses (RF) with magnetic field gradients(g), according to Figure S8.

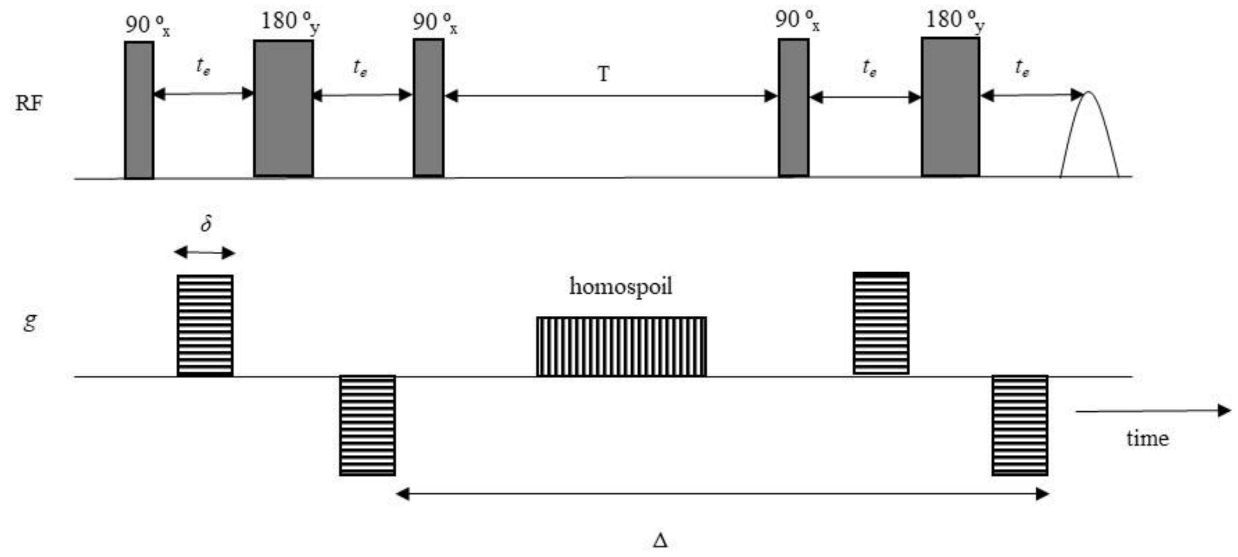

Figure S8. PGSTE pulse sequence showing gradient pulse duration δ, echo time t_e, storage interval T, homospoil gradient and diffusion time Δ.

The NMR signal attenuation of a PFG NMR experiment as a function of the gradient strength, $E(g)$, is related to the experimental variables and the diffusion coefficient (D) by:[6]

$$\frac{E(g)}{E_0} = \exp\left[-D \cdot \gamma_H^2 \cdot g^2 \cdot \delta^2 \cdot \left(\Delta - \frac{\delta}{3}\right)\right] \qquad (Eq. S7)$$

where E_0 is the NMR signal in the absence of gradient, γ_H is the gyromagnetic ratio of the nuclei being studied (i.e., ^{1}H in this case), g is the strength of the gradient pulse of duration δ, and Δ is the observation time (i.e., the time interval between the leading edges of the gradient pulses). The term $\gamma_H^2 \cdot g^2 \cdot \delta^2 \cdot \left(\Delta - \frac{\delta}{3}\right)$ is often referred to as the *b*-factor. Eq. S7 assumes a Gaussian distribution of the diffusing spins and it generally applies to free diffusion, such as the case of bulk liquids. However, this equation can also be applied for diffusion in porous materials with a quasi-homogeneous behavior, that is, with a macroscopically homogeneous pore structure, that shows a linear behavior of the PFG log plot of the signal attenuation.[7]

The measurements were performed by fixing Δ = 200 ms and δ = 4 ms. The magnitude of g was varied linearly with sixteen spaced increments. To achieve maximum signal attenuation, maximum values of g = 163 mT m^{-1} were necessary. All NMR measurements were performed at atmospheric

pressure and 25 °C. The diffusion coefficients D were calculated by fitting Eq. S7 to the experimental data.

The tortuosity is a structural property of a porous matrix, used to define and quantify the pore connectivity of a pore structure. Tortuosity values find much use as input parameters for modelling and molecular simulations of mass transport within porous materials.[8]

Tortuosity values are easily determined by the use of PFG-NMR experiments although consideration must be taken with regards to the identity of the probe molecule use. By taking the ratio of the free bulk liquid diffusivity, D_0, to the effective diffusivity of the liquid imbibed within the porous structure of the porous material of interest, D_{eff}, a dimensionless parameter, ξ, known as the "PFG interaction parameter" is obtained.[9] This relation is shown in Eq. S8.

$$\frac{D_0}{D_{\mathrm{eff}}} = \xi \qquad (Eq. S8)$$

Although this ratio has commonly been referred to as the tortuosity of a porous medium, this is not strictly true. The tortuosity (τ) of a porous medium determined using PFG-NMR is found using the following equation:

$$\frac{D_0}{D_{\mathrm{eff}}} = \tau \qquad (Eq. S9)$$

The key difference between Eq. S8 and S9 is that whilst Eq. S8 refers to the diffusivity of any molecule, Equation S9 is valid only for molecules that weakly interact with the surface of the porous material of interest. Therefore, it should be clear that the selection of an appropriate guest molecule for the system under study is vital to determining an accurate value for the actual tortuosity of a porous medium. D'Agostino et al. demonstrated that liquid alkanes are the most appropriate choice for determining the tortuosity of porous materials using PFG-NMR due to their lack of chemical functionality rendering them incapable of interaction with the catalyst surface.[7] This ensures that the obtained value of tortuosity is dependent only on the pore connectivity and is unaffected by molecular interactions that could otherwise alter the diffusivity of the guest molecule. Because of this, *n*-octane was chosen as the guest molecule for determining the tortuosity of the Al_2O_3 carriers. Previous work has shown that short chain liquid alkanes give reliable values of τ regardless of molecular dimension size. Log attenuation plots obtained by

fitting Equation S7 to the data obtained are shown below in Figure S9a. Obtained diffusion coefficients and tortuosities are shown in Table S6. Obtained tortuosities are expressed graphically in Figure S9b.

Table S6. Self-diffusion coefficients and calculated tortuosity values of the various photocatalysts determined using PFG NMR diffusometry of imbibed *n*-octane (free bulk self-diffusivity also reported). The error of all self-diffusion coefficients and calculated tortuosity values is 3%.

**Dopant**	**D (m^2s^{-1}) · 10^{10}**	**τ (-)**
am-CN	6.3 ± 0.2	3.8 ± 0.1
CNO	10.3 ± 0.3	2.3 ± 0.1
g-CN	6.0 ± 0.2	4.0 ± 0.1
ox-CN	10.4 ± 0.3	2.3 ± 0.1
mw-CN	8.6 ± 0.3	2.8 ± 0.1
Bulk *n*-octane	24.1 ± 0.7	-

There is no evident curvature present in the log attenuation plots, which would suggest that the diffusion is quasi-homogeneous.[10] Such behaviour is observed for porous materials with a macroscopically homogeneous root mean squared displacement (RMSD) that is much larger than the average pore size of the sample. That is, the probe molecule will collide with the pore walls many times. Consequently, the measured D_{eff} will be representative of the liquid confined within the porous medium and reduced by the tortuosity factor relative to the bulk free liquid Eq. S9.[10] Obtained tortuosity values are typical of mesoporous materials and Figure S9 suggests that oxidative and microwave treatments result in less tortuous pore structures, potentially with increased mass transport properties when compared to the pristine *g*-CN. Conversely, *am*-CN shows no significant change in pore tortuosity when compared to the pristine *g*-CN.

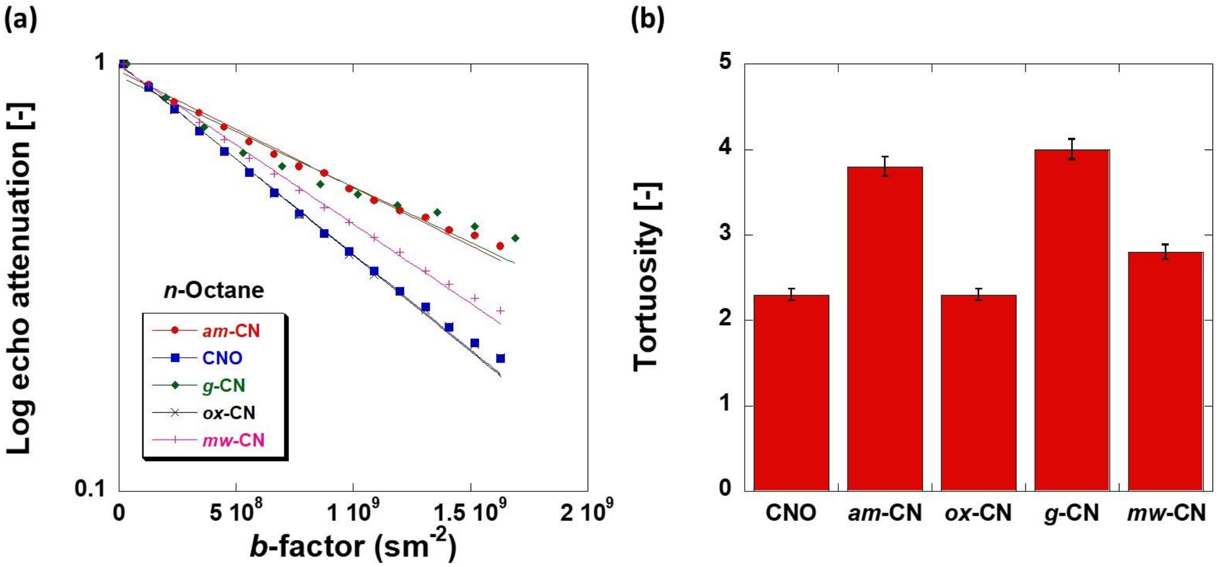

Figure S9. (a) Log attenuation plots obtained using n-octane imbibed within the pores of the various photocatalysts and (b) the calculated tortuosity values. The error of all tortuosity values is approximately 3%.

Nanoresolved IR measurements

The spectra, shown at Figure S10, closely resemble the ones acquired with FTIR, with direct correspondence of main peaks present. An overall enhancement of the absolute absorption is observed for *mw*-CN, if compared to *g*-CN, similarly to what observed with FTIR. In addition, *mw*-CN spectra showed a higher standard deviation (shown as shadow in Figure S10) compared to *g*-CN, suggesting that functional groups of *mw*-CN are less homogenous at the nanoscale, Interestingly, the peak at 1648 cm^{-1} (associated to C=N asymmetric stretching) is shifted to 1648 cm^{-1}. This shift was also observed in FTIR spectra but with minor extent. In regard to relative absorption, the peaks in the spectral region between 1400-1600 cm^{-1}, mainly due to C-N stretching, show enhanced intensity.

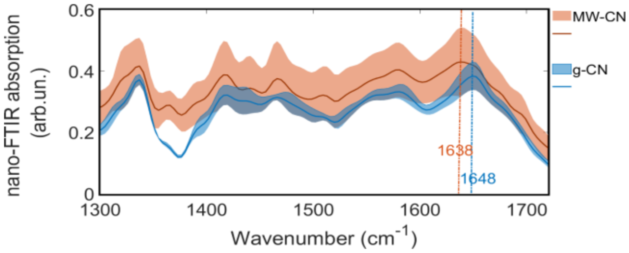

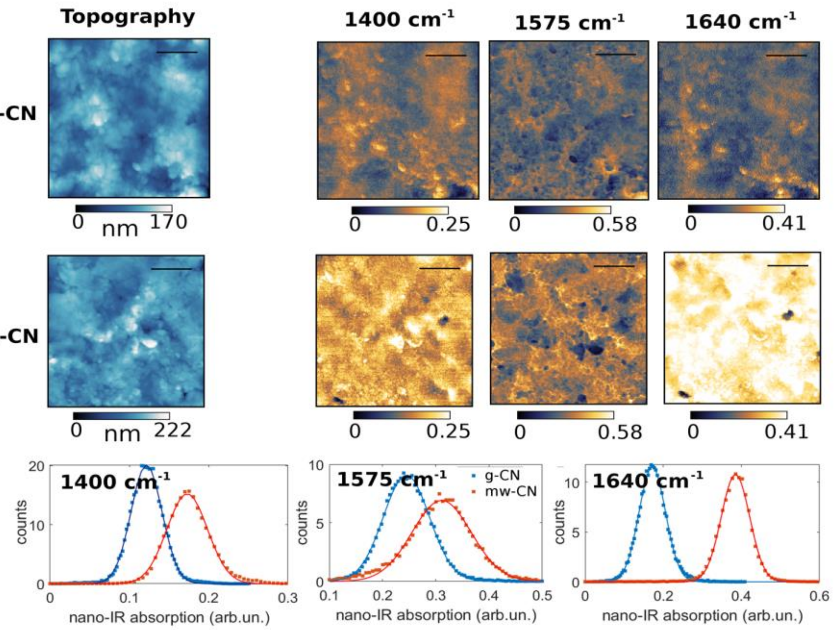

Figure S10. (up) s-SNIM spectroscopy and imaging. Average (n = 6) nano IR absorption spectra of *g*-CN and *mw*-CN acquired on areas 20 nm wide. (down) Topography maps (AFM) and one-colour absorption maps of *g*-CN and *mw*-CN acquired at three different wavenumbers, from left to right respectively at 1400, 1575 and 1640 cm^{-1}. Phase distribution plots, with *g*-CN (red) and *mw*-CN (blue) at the three selected wavenumbers are shown.

On the basis of s-SNOM and FTIR results we selected 1400 and 1575 related to C-N modes and 1640 cm^{-1} due to C=N vibrations for imaging analysis. AFM maps, showing the samples morphology and s-SNOM maps, proportional to sample absorption, are shown in the lower panel of Figure S10. As a first remark, nanoresolved imaging revealed the enhancement of the signal for *mw*-CN, similarly to what observed with spectroscopy. Moreover, the presence of high-contrast spots in imaging maps reflects possible subsurface porosity of the materials, apparently more visible for *mw*-CN.

Statistical analysis performed by considering phase distribution on the whole areas imaged suggests that *mw*-CN absorptions are slightly less homogeneously distributed at the nanoscale than *g*-CN.

EPR sample preparation

EPR measurements were performed on bare *g*-CN and *mw*-CN powders as well as on Ni/*mw*-CN samples. The Ni/*mw*-CN sample (≈ 20 mg) was outgassed and dehydrated under dynamic vacuum at 393 K for 30 min in a quartz cell designed for *in situ* EPR experiments (Figure S11).

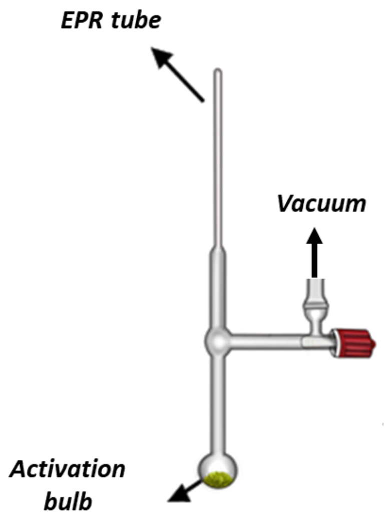

Figure S11. EPR cell for in situ thermal and gas adsorption experiments.

The Ni reducibility was tested by heating the dehydrated sample *in situ* under H_2 atmosphere (30 mbar nominal pressure) for 30 minutes at 573 K. NO adsorption experiments were performed in a similar way by contacting the dehydrated sample with 10 mbar of NO at RT. Experiments on bare *g*-CN and *mw*-CN were performed on as synthesized samples, without dehydration (Figure S12).

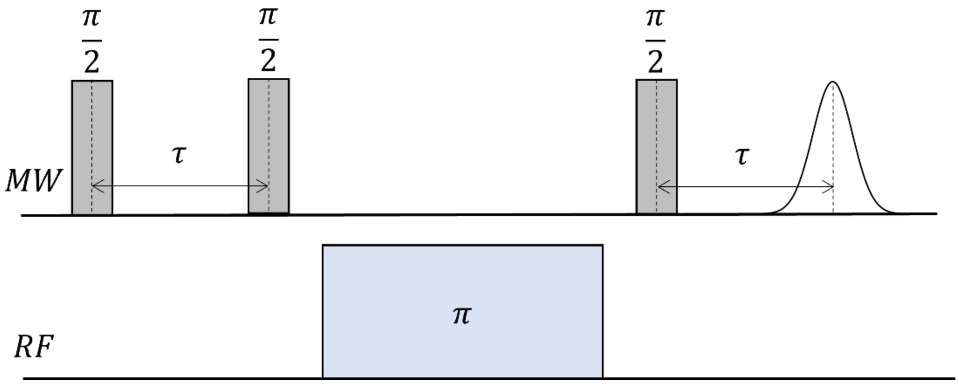

Figure S12. Mims-ENDOR pulse sequence used to record the ENDOR spectra on *g*-CN and *mw*-CN.

EPR measurements on *g*-CN and *mw*-CN

In order to compare the defect structure of *g*-CN and *mw*-CN, Q-band CW-EPR and ENDOR experiments were performed. The Q-band CW-EPR spectra of the two samples are shown in Figure S13a. The two spectra are practically superimposable and can be rationalized considering two main species (Table S7) as recently reported by some of us.[11] This result demonstrates that the paramagnetic defect structure of the two samples is similar.

To further investigate the local structure of the paramagnetic species Mims-ENDOR experiments were carried out. The ENDOR spectrum reports the relative intensity change of the EPR signal as a function of the applied RF frequency (Figure S13). ENDOR lines appear when the resonance condition $\nu^{\pm}_{ENDOR} = |\nu_I \pm A/2|$ is met, where ν_I is the Larmor frequency of the nucleus under investigation (given by $\nu_I = g_n\mu_n B_0/h$) and A is the orientation dependent hyperfine coupling containing both the isotropic and anisotropic contributions to the hyperfine tensor. The spectra reported in Figure S13b are centered at the proton Larmor frequency and display a dipolar hyperfine coupling (A^H) of the order of 0.8 MHz. The absence of clearly resolved components suggests that in both cases the paramagnetic species share a relatively heterogeneous local environment, with a minimum electron spin-proton distance of the order of 4.3 Å as derived from a point-dipolar approximation.[11]

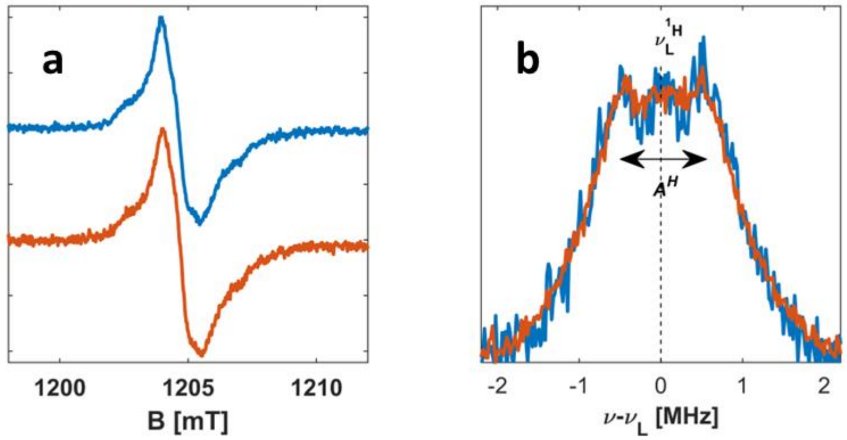

Figure S13. a) Q band CW EPR spectra recorded at room temperature. b) Mims ENDOR spectra recorded at the field position correspondent to the maximum echo intensity (1204.6 mT); spectra are centred at the proton Larmor frequency. Spectra corresponding to *g*-CN and *mw*-CN are

displayed in blue and red respectively. A mean 1H hyperfine coupling (AH) of ≈ 1 MHz can be derived from the ENDOR spectra recorded on both *g*-CN and *mw*-CN samples.

EPR measurements on Ni/*mw*-CN

Ni^{2+} ($3d^8$) is characterized by spin states which depend on the local ligand field symmetry. In the case of tetrahedral or octahedral symmetries Ni^{2+} features a triplet state $S = 1$, while in the case of square planar coordination a diamagnetic S=0 state is expected. In both cases, Ni^{2+} centers cannot be observed by means of X-band CW-EPR because of the large zero-field splitting in the case of the S=1 triplet states or because the diamagnetic nature of S=0 states. However, Ni^+ ($3d^9$, $S = 1/2$) sites can be probed directly with EPR, as they display a characteristic pseudo-axial signal with $g_3>g_{1,2}>g_e$, where g_e is the free electron g value (g_e=2.0023) As expected, the Ni/*mw*-CN sample shows a CW-EPR spectrum at 77 K characterized by the presence of the weak radical signal previously discussed (labelled R in Figure S14a). Upon reduction with H_2 however, a new spectrum is observed characterized by a **g** matrix (Table S7) consistent with a $3d^9$ electron configuration and a semi occupied molecular orbital (SOMO) with dominant Ni $3d_{x2-y2}$ character. While this experiment proves the presence of reducible Ni^{2+} species under relatively harsh conditions, the adsorption of NO molecules provides a simple and effective means to reveal the presence of coordinatively unsaturated and chemically accessible surface Ni^{2+} species. NO is a 11e $^2\Pi$ molecular radical (S=1/2), which is well known to coordinate to Ni^{2+} forming a paramagnetic S=1/2 $[Ni\text{-}NO]^{2+}$ adduct.[12]

The EPR spectrum of Ni/*mw*-CN recorded at 77K under 10 mbar NO is reported in Figure S14 (see also main text) and consists of two clearly separated signals. The one at high field, indicated with an asterisk, is due to monomeric NO molecules weakly adsorbed on the *mw*-CN surface, it has been widely discussed elsewhere[13] and we will not comment on it further. The second signal, on the low field is due to the $[Ni\text{-}NO]^{2+}$ adduct and display a pseudo-axial symmetry with g_1= 2.140 ± 0.002 g_2= 2.174 ± 0.002 and g_3= 2.290 ± 0.004 in line with previous reports for mononytrosyl Ni^{2+} adducts on solid state systems (see Table S7). The interaction is reversible and upon NO evacuation both signals disappear after pumping off at RT and can be restored by a new NO adsorption. The formation of the $[Ni\text{-}NO]^{2+}$ adduct provides direct evidence for the presence of chemically accessible and reactive Ni^{2+} species at the surface of Ni/*mw*-CN.

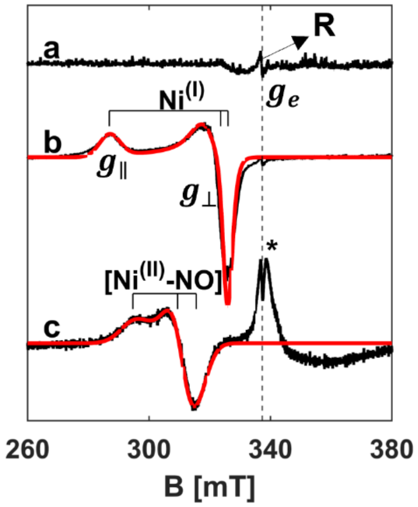

Figure S14. Normalised CW EPR spectra of a) dehydrated Ni/*mw*-CN b) H_2 reduced Ni/*mw*-CN and c) dehydrated Ni/*mw*-CN under 10 mbar NO. All spectra were recorded at 77 K. Computer simulation of the spectra is reported in red, the corresponding spin-Hamiltonian parameters are listed in Table S7. The asterisk indicates the signal due to physisorbed molecular NO.

Table S7. Spin-Hamiltonian parameters derived from computer simulation of the paramagnetic species described in the text.

**Sample**	g_1	g_2	g_3	***REF***
g-CN / *mw*-CN	$2.0063 \pm 0,0002$	$2.0049 \pm 0,0003$	$2.0036 \pm 0,0004$	
	$2.0080 \pm 0,0002$	$2.0050 \pm 0,0002$	$2.0023 \pm 0,0004$	
Ni/*mw*-CN reduced in H_2	2.075 ± 0.002	2.101 ± 0.002	2.353 ± 0.002	
Ni/*mw*-CN-NO adduct	2.140 ± 0.002	2.174 ± 0.002	2.290 ± 0.004	
Ni/MgO-NO adduct	2.131	2.131	2.274	[12a]
Ni/ZSM5-NO adduct	2.154	2.196	2.347	[12b]

Computational methodology

All structures were optimized at the B3LYP level of theory in the ground state, with non-metal atoms treated with the Ahlrichs basis-set Def2SVP, whereas the Los-Alamos Effective-Core Potential (ECP) basis-set LanL2DZ was used for the nickel atom. A solvent cavity of *N*,*N*-

dimethylformamide (DMF, ε ≈ 37) was added in the ground-state optimizations as a Polarizable Continuum Model (PCM). The theoretical models were built and visualized by use of the GaussView 6.0 software as square-planar starting geometries presenting a Ni(II) metallic center with a d^8 valence electronic configuration. The excited-state calculations were performed on the previously optimized structures by use of Time-Dependent DFT (TD-DFT) at the HSE06 level of theory. The excited-state electronic structures were visualized with the VESTA software. Molecular models for the carbon nitride binding sites coordinated to nickel were used for the TD-DFT calculations, because this opens up a wide range of hybrid functionals employable in the evaluation of the electronic properties, which would be computationally prohibitive for periodic equivalents.[14]

Reference complexes

To assess the capacity of pyrrolidine to displace DMF around nickel, we considered a series of Ni(II) complexes with gradual replacement of DMF molecules by pyrrolidine molecules (Figure S15). The complexation energies (Table S8) were estimated as the difference between the total energy of the complex and those of the separate components.

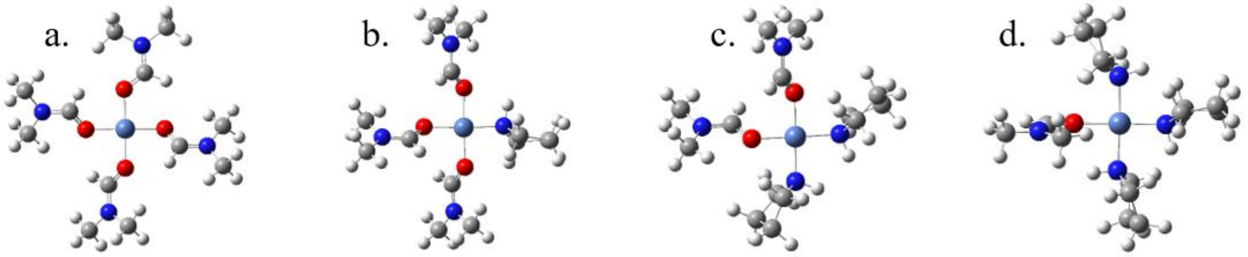

Figure S15. The reference complexes a. Ni_4_DMF; b. Ni_3_DMF_1pyrr; c. Ni_2_DMF_2pyrr; d. Ni_1_DMF_3pyrr.

Table S8. Complexation energies of the Ni(II) complexes corresponding to the sequential displacement of DMF by pyrrolidine.

Complexes	Complexation energies (kcal/mol)

Ni_4_DMF	–97.0
Ni_3_DMF_1pyrr	–106.3
Ni_2_DMF_2pyrr	–112.3
Ni_1_DMF_3pyrr	–111.8

(Defective) carbon nitride complexing nickel

We selected three molecular models to represent sites at surface of pristine and mw-CN where complexation with Ni could take place. Pristine *g*-CN is represented by an amino-substituted heptazine molecule and we considered complexation either with the NH_2 group or with one peripheral nitrogen atom of the heptazine unit (see structure a in Figure S16). For *mw*-CN, we considered: (*i*) a triazole-defective heptazine moiety, with Ni complexation either at the secondary or tertiary nitrogen of the triazole ring (see structure b in Figure S16, and (*ii*) an open defective moiety having one amine and one imine group (see structure c in Figure S16).

In the lower part of Figure S16, the corresponding Ni(II) complexes are shown, with each CN ligand substituting a second DMF molecule in the reference complexes. Table S9 and S10 reports the complexation energies in kcal/mol. Figure S17 shows the HOMOs and LUMOs of those complexes.

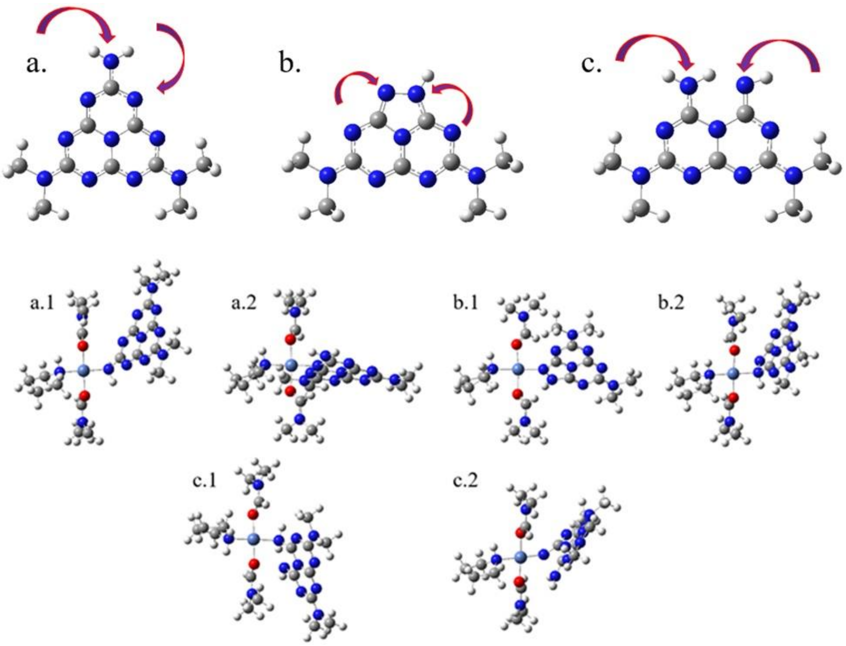

Figure S16. Models of pristine *g*-CN and *mw*–CN, as a. pristine b. triazole-defective heptazine and c. defective amine/imine moiety, with the indication of the two possible binding sites in each of them: the $–NH_2$ group and one peripheral N atom in a.; the peripheral N and NH in b.; the amine/imine groups in c. Entries from a.1 to c.2 show the corresponding Ni^{2+} complexes with CN ligands in the coordinative sites indicated above.

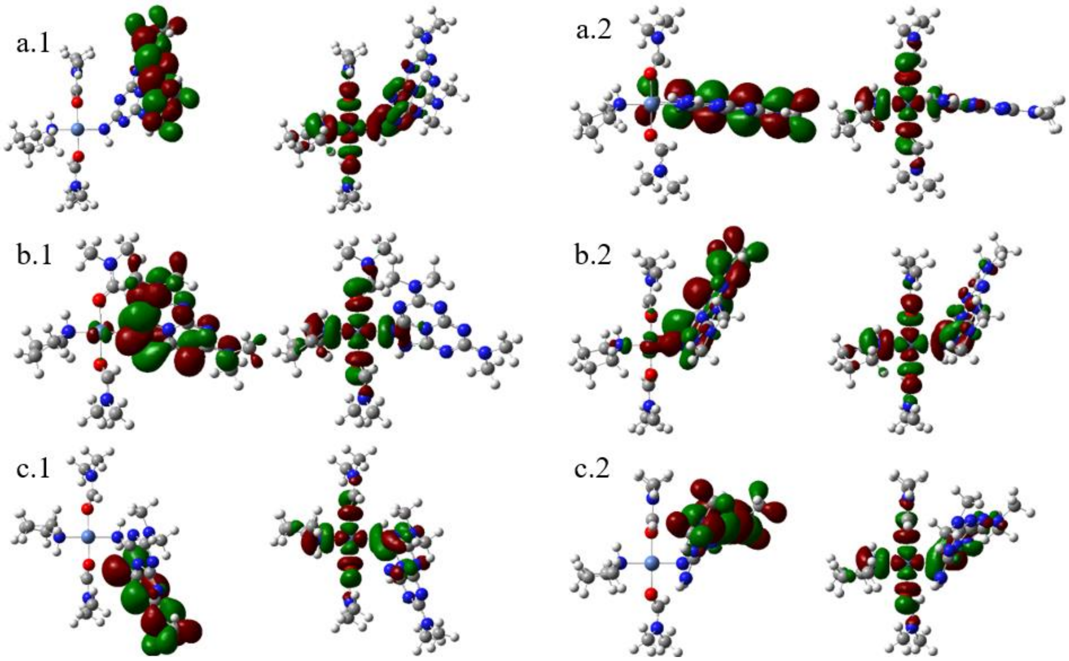

Figure S17. Electronic structures of the Ni(II) complexes with CN ligands. For each set, the left pictures are the HOMOs, whereas the right pictures are the LUMOs.

Table S9. Complexation energies of the Ni(II) complexes containing the CN models as ligands.

Complexes	Complexation energies (kcal/mol)
–NH_2-coordinating Pristine	–86.6
N-coordinating Pristine	–96.6
N-coordinating Triazole	–94.6
NH-coordinating Triazole	–79.5
Defective Imine	–101.0
Defective Amine	–90.5

Table S10. Complexation energies of the Ni(I) complexes containing the CN models as ligands.

Complexes	Complexation energies (kcal/mol)
–NH_2-coordinating Pristine	+23.0

N-coordinating Pristine	+11.0
N-coordinating Triazole	+10.0
NH-coordinating Triazole	+30.5
Defective Imine	+13.8
Defective Amine	+25.6

Excited-state calculations

Below the Attachment/Detachment Densities for the three most stable Ni(II) complexes with pristine/defective CN ligands are reported, along with the wavelengths and oscillator strengths related to each state (Table S11, S12 and S13). The hole and electron densities are represented in light blue and yellow, respectively (Figure S18, S19 and S20).

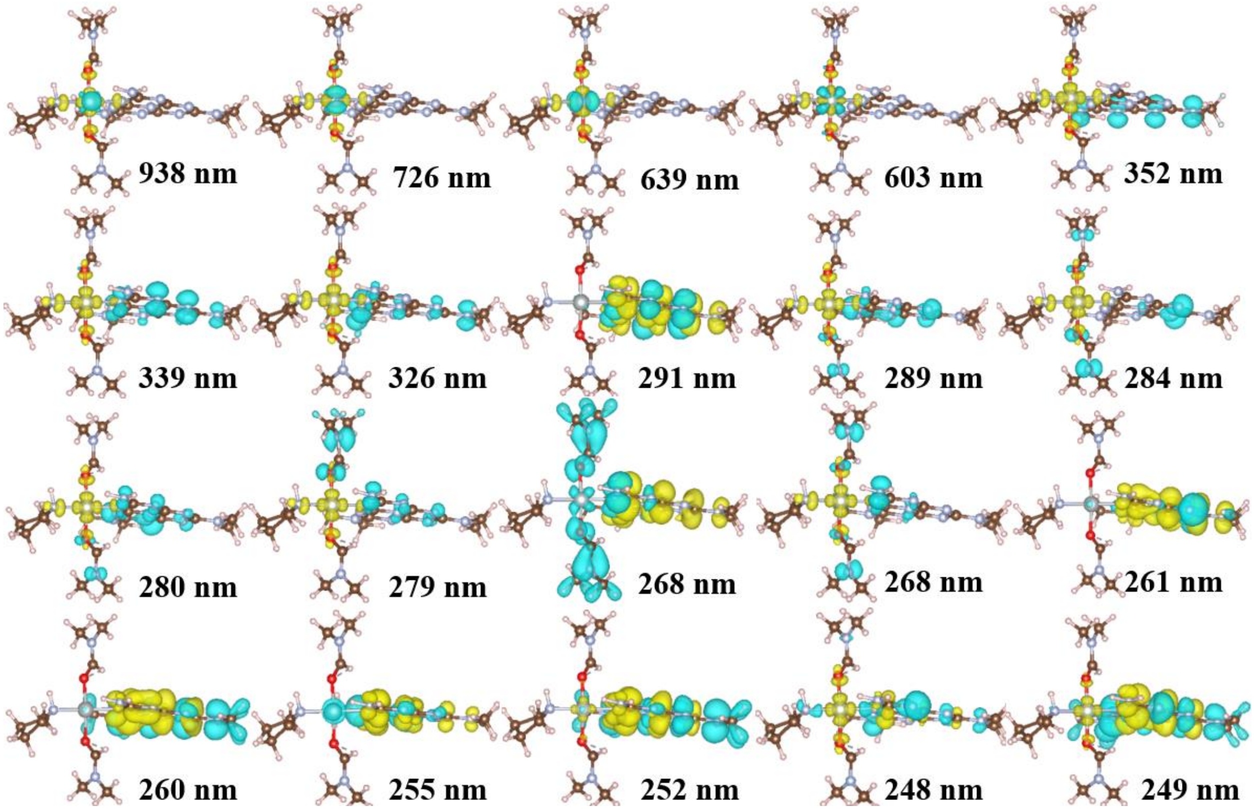

Figure S18. Computed Attachment/Detachment Densities of the Pristine N-coordinated Ni complex, listed from 1 to 20 from top-left to bottom-right, depicting the hole (h^+ in light-blue) and the electron (e^- in yellow) of each transition.

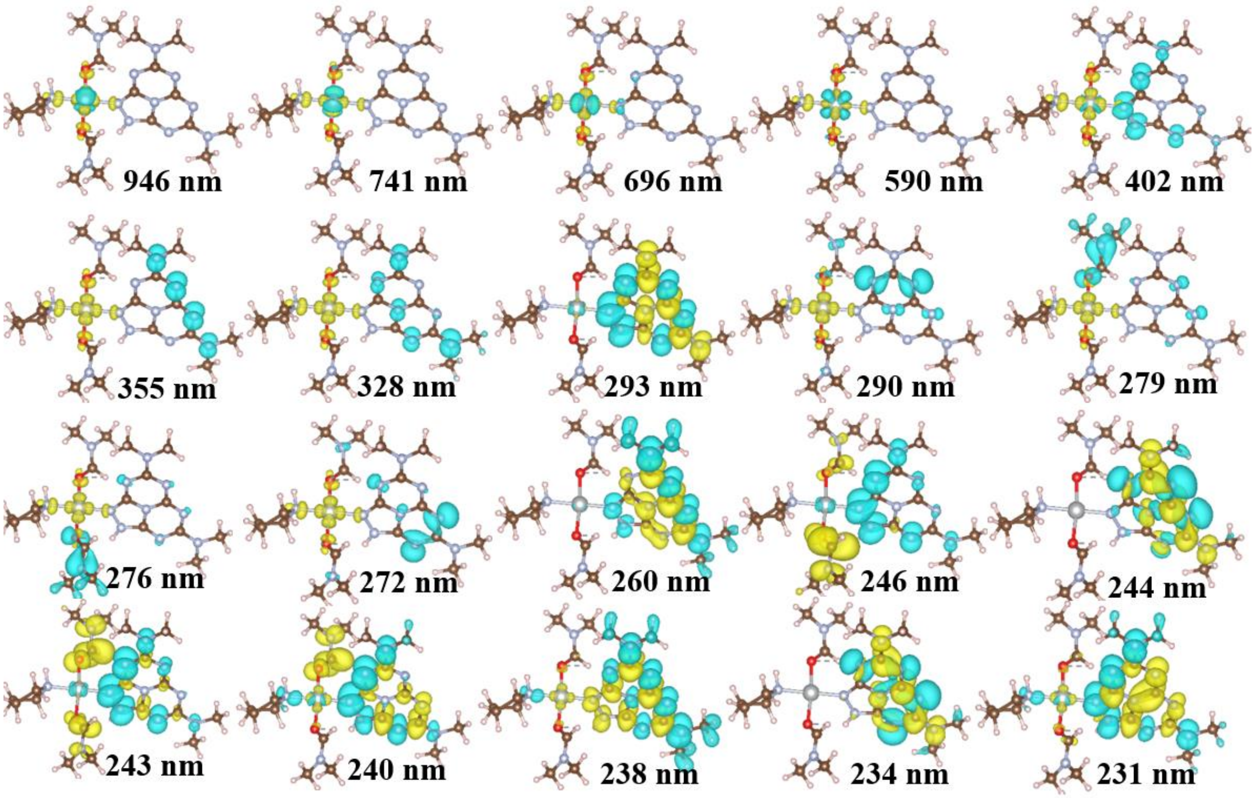

Figure S19. Computed Attachment/Detachment Densities of the Triazole N-coordinated Ni complex, listed from 1 to 20 from top-left to bottom-right, depicting the hole (h^+ in light-blue) and the electron (e^- in yellow) of each transition.

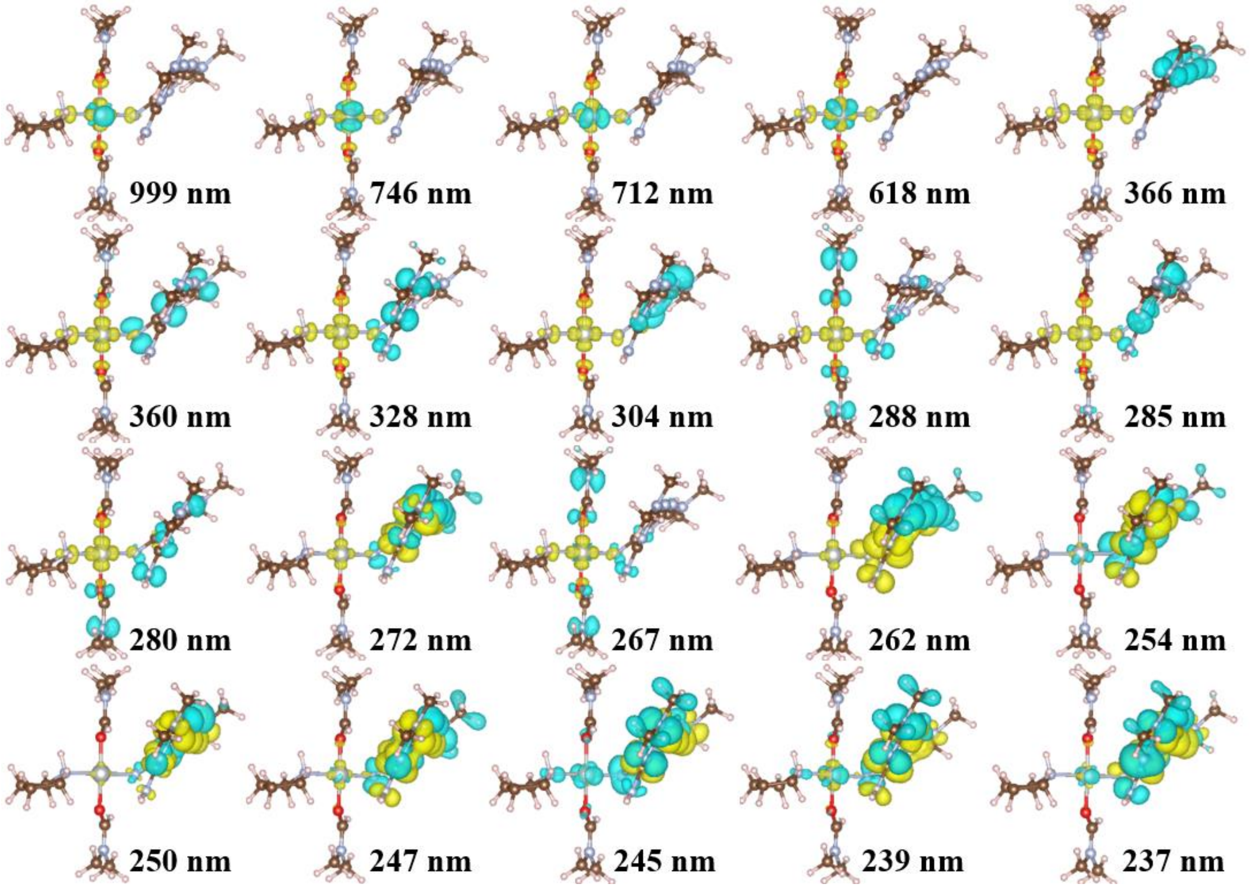

Figure S20. Computed Attachment/Detachment Densities of the Defective imine-coordinated Ni complex, listed from 1 to 20 from top-left to bottom-right, depicting the hole (h^+ in light-blue) and the electron (e^- in yellow) of each transition.

Table S11. Excitation's wavelengths (λ) and Oscillator Strengths computed for the Pristine N-coordinated Ni complex.

**N-coordinating Pristine**	
**Wavelength (nm)**	**Oscillator Strength (arb. units)**
938	0.0001
726	0
639	0
603	0.0001
352	0.0001
339	0.0049
326	0.0014
291	0.0358
289	0.0006
284	0.0362
280	0.0084
279	0.0244
268	0.3443
268	0.0092
261	0.0005
260	0.0106
255	0.0008
252	0.0247
249	0.5607
248	0.0551

Table S12. Excitations' wavelengths (λ) and Oscillator Strengths computed for the Triazole N-coordinated Ni complex.

N-coordinating Triazole	
**Wavelength (nm)**	**Oscillator Strength (arb.units)**
943	0.0001
741	0
696	0.0001
590	0.0002
402	0.0037
355	0
328	0.0002
293	0.0063
290	0.0328
279	0.0030
276	0.0075
272	0.0011
260	0.4804
246	0.0393
244	0.0028
243	0.1409
240	0.3389
238	0.3552
234	0.0004
231	0.3323

Table S13. Excitations' wavelengths (λ) and Oscillator Strengths computed for the defective imine-coordinated Ni complex.

**Defective imine**	
**Wavelength (nm)**	**Oscillator Strength (arb. units)**
999	0
746	0
712	0.0020
618	0.0007
366	0.0024
360	0.0879
328	0.0377
304	0.0381
288	0.0062
285	0.0037
280	0.0113
272	0.2170
267	0.0116
262	0.0145
254	0.0378
250	0.0868
247	0.3751
245	0.4130
239	0.5246
237	0.1501

References

[1] aK. R. Brownstein, C. E. Tarr, *J. Magn. Reson.* **1977**, *26*, 17-24; bJ. H. Strange, J. Mitchell, J. B. W. Webber, *Magn. Reson. Imaging* **2003**, *21*, 221-226.

[2] N. Robinson, L. F. Gladden, C. D'Agostino, *Faraday Discuss.* **2017**, *204*, 439-452.

[3] C. D'Agostino, J. Mitchell, M. D. Mantle, L. F. Gladden, *Chem. Eur. J.* **2014**, *20*, 13009-13015.

[4] aC. D'Agostino, G. L. Brett, P. J. Miedziak, D. W. Knight, G. J. Hutchings, L. F. Gladden, M. D. Mantle, *Chem. Eur. J.* **2012**, *18*, 14426-14433; bC. D'Agostino, M. R. Feaviour, G. L. Brett, J. Mitchell, A. P. E. York, G. J. Hutchings, M. D. Mantle, L. F. Gladden, *Catal. Sci Technol.* **2016**, *6*, 7896-7901; cC. D'Agostino, T. Kotionova, J. Mitchell, P. J. Miedziak, D. W. Knight, S. H. Taylor, G. J. Hutchings, L. F. Gladden, M. D. Mantle, *Chem. Eur. J.* **2013**, *19*, 11725-11732.

[5] E. O. Stejskal, J. E. Tanner, *J. Chem. Phys.* **1965**, *42*, 288-292.

[6] E. O. Stejskal, *J. Chem. Phys.* **1965**, *43*, 3597-3603.

[7] C. D'Agostino, J. Mitchell, L. F. Gladden, M. D. Mantle, *J. Phys. Chem. C* **2012**, *116*, 8975-8982.

[8] S. Ferreira, J. J. Verstraete, E. Jolimaitre, D. Leinekugel-le-Cocq, C. Jallut, in *Comput.-Aided Chem. Eng, Vol. 40* (Eds.: A. Espuña, M. Graells, L. Puigjaner), Elsevier, **2017**, pp. 91-96.

[9] M. D. Mantle, D. I. Enache, E. Nowicka, S. P. Davies, J. K. Edwards, C. D'Agostino, D. P. Mascarenhas, L. Durham, M. Sankar, D. W. Knight, L. F. Gladden, S. H. Taylor, G. J. Hutchings, *J. Phys. Chem. C* **2011**, *115*, 1073-1079.

[10] M. Dvoyashkin, R. Valiullin, J. Kärger, *Phys. Rev. E* **2007**, *75*, 041202.

[11] A. Actis, M. Melchionna, G. Filippini, P. Fornasiero, M. Prato, E. Salvadori, M. Chiesa, *Angew. Chem. Int. Ed.* **2022**, *61*, e202210640.

[12] aM. Chiesa, M. C. Paganini, E. Giamello, C. D. Valentin, G. Pacchioni, *J. Mol. Catal. A Chem.* **2003**, *204-205*, 779-786; bP. Pietrzyk, K. Góra-Marek, T. Mazur, B. Mozgawa, M. Radoń, M. Chiesa, Z. Zhao, Z. Sojka, *J. Catal.* **2021**, *394*, 206-219.

[13] M. Chiesa, E. Giamello, M. Che, *Chem. Rev.* **2010**, *110*, 1320-1347.

[14] aT. Suter, V. Brázdová, K. McColl, T. S. Miller, H. Nagashima, E. Salvadori, A. Sella, C. A. Howard, C. W. M. Kay, F. Corà, P. F. McMillan, *J. Phys. Chem. C* **2018**, *122*, 25183-25194; bX. Song, X. Li, X. Zhang, Y. Wu, C. Ma, P. Huo, Y. Yan, *Appl. Catal. B* **2020**, *268*, 118736.
